# Selective Activation of Peptide-Thioester Precursors for Templated Native Chemical Ligations

**DOI:** 10.1002/anie.202413644

**Published:** 2024-10-25

**Authors:** Paul Spaltenstein, Riley J. Giesler, Samuel R. Scherer, Patrick W. Erickson, Michael S. Kay

**Affiliations:** Department of Biochemistry, University of Utah, 15 North Medical Drive East, Room 4100 Salt Lake City, UT, 84112, United States; Department of Biochemistry, University of Utah, 15 North Medical Drive East, Room 4100 Salt Lake City, UT, 84112, United States; Department of Biochemistry, University of Utah, 15 North Medical Drive East, Room 4100 Salt Lake City, UT, 84112, United States; Department of Biochemistry, University of Utah, 15 North Medical Drive East, Room 4100 Salt Lake City, UT, 84112, United States; Aizen Therapeutics, 1927 Pasco Rancho Castilla, Los Angeles, CA, 90032, United States; Department of Biochemistry, University of Utah, 15 North Medical Drive East, Room 4100 Salt Lake City, UT, 84112, United States

**Keywords:** native chemical ligation, chemical protein synthesis, templated peptide ligation, peptide-thioester precursor, peptide conjugation

## Abstract

Chemical protein synthesis enables access to proteins that would otherwise be difficult or impossible to obtain with traditional means such as recombinant expression. Chemoselective ligations provide the ability to join peptide segments prepared by solid-phase peptide synthesis. While native chemical ligation (NCL) is widely used, it is limited by the need for C-terminal thioesters with suitable reaction kinetics, properly placed native Cys or thiolated derivatives, and peptide segment solubility at low mM concentrations. Moreover, repetitive purifications to isolate ligated products are often yield-sapping, hampering efficiency and progress. In this work, we demonstrate the use of Controlled Activation of Peptides for Templated NCL (CAPTN). This traceless multi-segment templated NCL approach permits the one-pot synthesis of proteins by harnessing selective thioester activation and orthogonal conjugation chemistries to favor formation of the full-length ligated product while minimizing side reactions. Importantly, CAPTN provides kinetic enhancements allowing ligations at sterically hindered junctions and low peptide concentrations. Additionally, this one-pot approach removes the need for intermediate purification. We report the synthesis of two *E. coli* ribosomal subunits S16 and S17 enabled by the chemical tools described herein. We anticipate that CAPTN will expedite the synthesis of valuable proteins and expand on templated approaches for chemical protein synthesis.

## Introduction

Peptide ligation methods have expanded the scope of chemical protein synthesis (CPS) and semi-synthetic approaches enabling the study of various proteins that would otherwise be difficult or impossible to access using traditional biochemical and recombinant means.^[1]^ Applications of CPS include functional enzymes for mirror-image biology,^[2]^ targets for mirror-image phage display to identify D-peptide inhibitors of protein-protein interactions,^[3]^ and homogeneous site-specific post-transitionally modified proteins,^[4]^ among others. These endeavors rely on the ability to chemically prepare proteins which typically includes ligation of synthetic peptides prepared by solid-phase peptide synthesis (SPPS).^[5]^ Chemoselective ligation techniques are required as they enable site-specific extension of peptide segments into full-length proteins. Native chemical ligation (NCL)^[6]^ has proven to be the most widely used approach although the restriction imposed on the amino acid sequence (i.e., suitable thioester residue and Cys at the ligation junction) has prompted the development of complementary and alternative techniques. Desulfurization, first applied to Ala ligation junctions, greatly extends the concept of NCL beyond Cys and is now ubiquitous.^[7]^ Encouraged by the rapid success of this methodology, improved desulfurization methods^[8]^ and other thiolated residues (e.g., Val derivatives) have been reported.^[9]^ These thiolated surrogates provide coverage of a wider range of protein sequences, but have failed to become standard practice due to additional handling steps, slower kinetics, or the lack of commercial availability. Efforts to develop alternative ligation chemistries besides NCL include Ser/Thr ligation (STL),^[10]^ Cys/Pen ligation (CPL),^[11]^ α-keto acid-hydroxylamine (KAHA),^[12]^ and reductive diselenide-selenoester ligation (rDSL).^[13]^ These strategies have been successfully demonstrated on a number of proteins, but remain less common. In addition to altering the reactive species involved in NCL (thiol/thioester), methodologies to streamline the overall CPS process have become essential such as reliable SPPS of peptide-thioester precursors compatible with in situ activation. The peptide hydrazide (-NHNH_2_)^[14]^ and peptide N-acylurea (*N*-acyl-benzimidazolinone; -Nbz)^[15]^ approaches are very popular and were critical for this work. Moreover, one-pot systems,^[16]^ solubilizing traceless tags,^[17]^ design of organocatalysts,^[18]^ auxiliary mediated ligations,^[19]^ and templated methods^[20]^ have contributed to the overall chemical CPS toolbox (see suggested reviews).^[21]^

**Figure 1.**
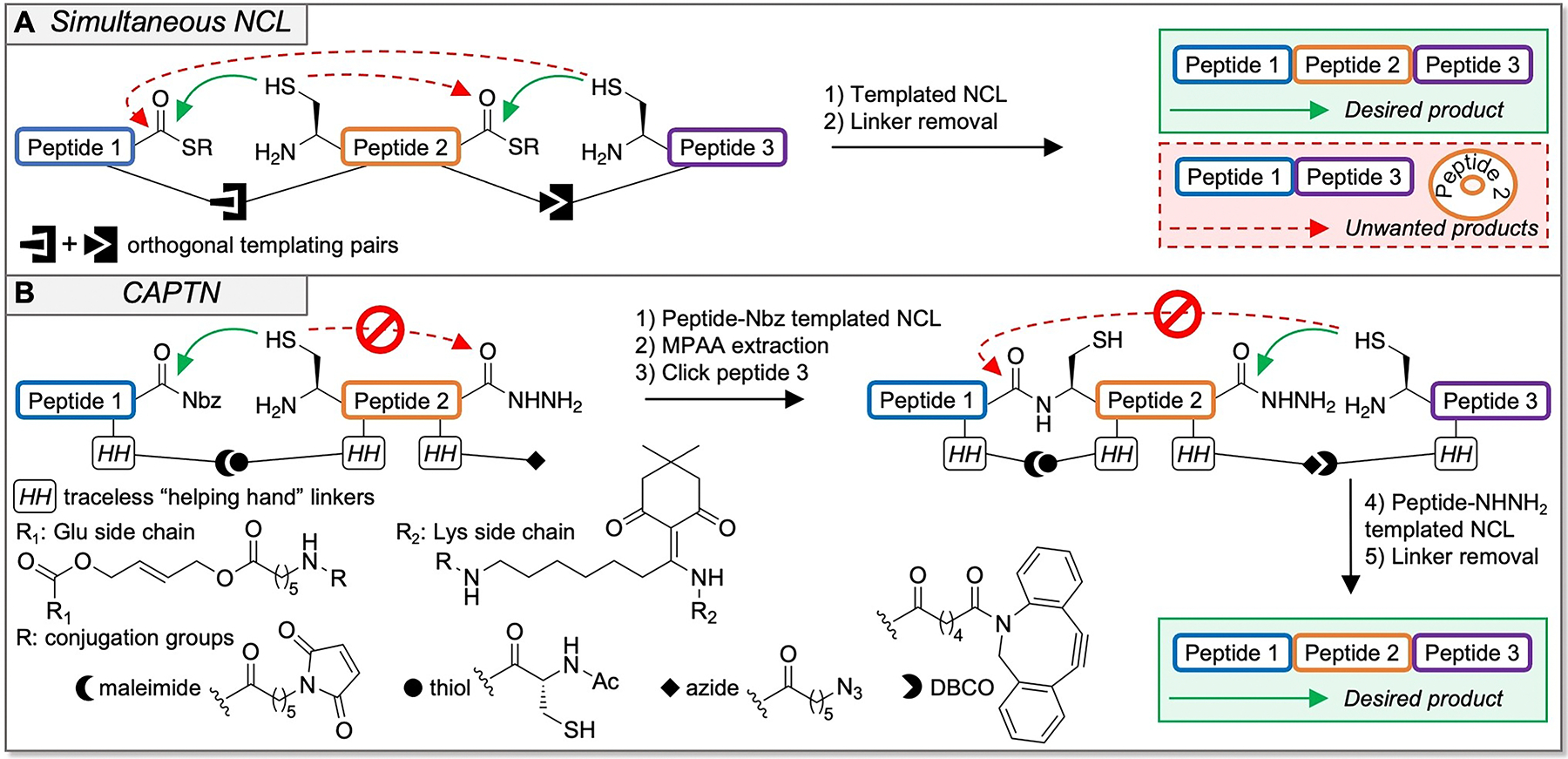
Multi-segment templated peptide ligations. (A) Simultaneous templated ligation of three peptide segments. Peptides functionalized with traceless tethers are conjugated via orthogonal templating pairs (e.g., short complementary DNA strands or clickable moieties). This templated approach improves reaction efficiency, but because of simultaneous ligations at both junctions, unwanted cross-ligation and internal peptide cyclization occur (red dotted arrows) competing with the desired reactions (green arrows). (B) Controlled Activation of Peptides for Templated NCL (CAPTN). Peptide 1 and 2 are tethered together via maleimide/thiol conjugation and selective peptide-Nbz activation favors ligation between peptide 1 and 2 (green arrow) while preventing cyclization of peptide 2 (red dotted arrow). Clicking of peptide 3 then enables templated ligation to peptide 2 via peptide-NHNH_2_ activation (green arrow) without cross-ligations (red dotted arrow). This one-pot approach is compatible with our Lys and Glu “helping hand” traceless linkers. Peptide modifications are indicated by symbols, with corresponding chemical groups detailed in the figure legend.

We, among others, have reported a templated method for amide bond forming reactions.^[20e]^ Templating greatly enhances reaction rates (intramolecular, unimolecular kinetics), reducing unwanted side reactions such as thioester hydrolysis and allowing efficient ligations at low peptide concentrations (μM), thereby reducing the issues commonly associated with segment insolubility. Many templated approaches find applications outside of CPS, such as self-replicating peptides,^[22]^ labeling of proteins,^[23]^ detection and analysis,^[24]^ and drug development^[25]^ (see suggested reviews).^[26]^ Templated strategies for synthesis of native proteins require complete removal of the template tether to avoid affecting folding or activity due to unnatural chemical scars. Traceless photocleavable methods have been developed by Diederichsen and colleagues using peptide nucleic acids (PNA),^[20a]^ the Okamoto lab via peptide-DNA conjugates,^[20b]^ and the Devaraj group with micelle-mediated ligation of lipid-modified peptides.^[20d]^ Alternatively, Bode and co-workers demonstrated an elegant streptavidin-templated system with self-cleavage upon amide bond formation between hydroxylamine and acylboronate derivatives.^[20c]^ More recently, a split intein driven approach was reported by the Liu group.^[20f]^ While these approaches highlight the power of templated reactions (i.e., rapid and low concentrations), their applicability for total CPS is limited due to various constraints including the need for complex linker syntheses and incorporation of templating partners, additional handling steps, potential oxidation due to UV irradiation and copper-catalyzed click reactions, incorporation of highly hydrophobic moieties, and/or non-chemoselective ligation reactions. We previously introduced Click-Assisted NCL (CAN) to address these concerns.^[20e]^ CAN utilizes our traceless Lys(Ddap) “Helping Hand” (HH) linker,^[17a,d]^ functionalized on resin with a strained cyclooctyne (DBCO) or an azide. After peptide cleavage, in which the DBCO is protected from acid-mediated rearrangement with Cu(I), and purification, the peptides are clicked together via strain-promoted alkyne-azide cycloaddition (SPAAC), which is compatible with denaturants needed to handle poorly soluble peptides and efficient at low concentrations (sub-mM).^[20e,27]^ Activation of the peptide-NHNH_2_ is achieved by oxidation with NaNO_2_ (pH 3) and thiolysis with exogenous thiol (pH 5–6). The templated NCL is then initiated by carefully adjusting the pH to 6.8. A mild hydroxylamine treatment cleaves the linkers post-NCL and restores the native sequence. Notably, clicking of peptides, NCL, desulfurization, and linker removal are compatible with a one-pot approach. Except for the Okamoto method,^[20b]^ the major limitation for CPS of the technologies listed above (including CAN) is their constraint to templated reaction of two peptides. A multi-segment, one-pot templated system is highly desirable as it would remove yield-sapping purifications of assembly intermediates. Additionally, the kinetic enhancement from templating would allow access to unfavorable junctions that are generally avoided with standard intermolecular ligations. However, the formidable challenge of directing ligations at the desired junctions, all while preventing cross-ligations and peptide cyclization, remains to be addressed (Figure 1A). For instance, Okamoto and co-workers’ study on simultaneous templated ligation of three segments highlights the need to minimize unwanted ligations yielding significant side products.^[20b]^ To help address this problem, we report Controlled Activation of Peptides for Templated NCL (CAPTN); a one-pot three-segment templated NCL approach that yields full-length product with minimized unwanted side reactions (Figure 1B). CAPTN takes advantage of selective in situ activation of peptide-thioester surrogates and orthogonal conjugation chemistries to favor ligations at the desired junctions.

In this article, we aimed to extend our CAN methodology from a two-peptide segment system to multi-segment templated NCL. Moreover, even though CAN enhances our ability to synthesize proteins, we acknowledge that templating imposes additional restrictions as it involves the semi-permanent attachment of linkers. Although most often mitigated by the ability to use a sterically hindered ligation junction, CAN requires the presence of native Lys for HH attachments. We anticipated that our templated system could be performed using our recently reported Glu(AlHx) traceless HH linker^[17f]^ and access ligations beyond Cys or Ala due to the increased kinetics. We envisioned that the method must address the following considerations:

1. Enable one-pot traceless templated ligation of three segments with minimal cross-ligation and cyclization of the internal peptide.
2. Use orthogonal conjugation chemistries to selectively tether peptides together while ensuring stability in common CPS conditions.
3. Minimize handling steps and employ widely used SPPS, conjugation, and NCL chemistries.
4. Extend beyond Lys for the incorporation of traceless linkers needed for templated NCL.
5. Enable ligations at junctions other than Cys and Ala.

We demonstrate the use of CAPTN for CPS that meets these requirements and provides a robust templated system for peptide ligations. Importantly, CAPTN minimizes cross-ligation and cyclization that are expected when ligating three segments in one pot. This method, in conjunction with SPAAC used in CAN, employs maleimide/thiol conjugation to tether three peptides together in a stepwise manner. Selective thioester activation of peptide 1 is achieved via peptide-Nbz conversion while maintaining peptide 2 as an unreactive peptide-NHNH_2_, which diminishes unwanted peptide 2 cyclization. To address cross-ligation between peptide 1 and peptide 3, we use sequential conjugation reactions and template peptide 3 after the 1^st^ NCL. Peptide 2 is then converted to a peptide thioester by peptide-NHNH_2_ oxidation and thiolysis to initiate the 2^nd^ NCL (Figure 1B). In addition, we demonstrate successful NCL at Val junctions via kinetically enhanced penicillamine (Pen, thiolated Val) and establish templating compatibility of our Glu(AlHx) traceless HH linker with the templated NCL of the otherwise unfavorable intermolecular ligation of the *E. coli* ribosomal subunit S16 (82 residues); improving overall protein sequence coverage. Finally, we highlight the utility of CAPTN with the challenging chemical synthesis of the *E. coli* ribosomal subunit S17 (90 residues).

## Results and Discussion

### Maleimide/Thiol Conjugation for Templated NCL

**Figure 2.**
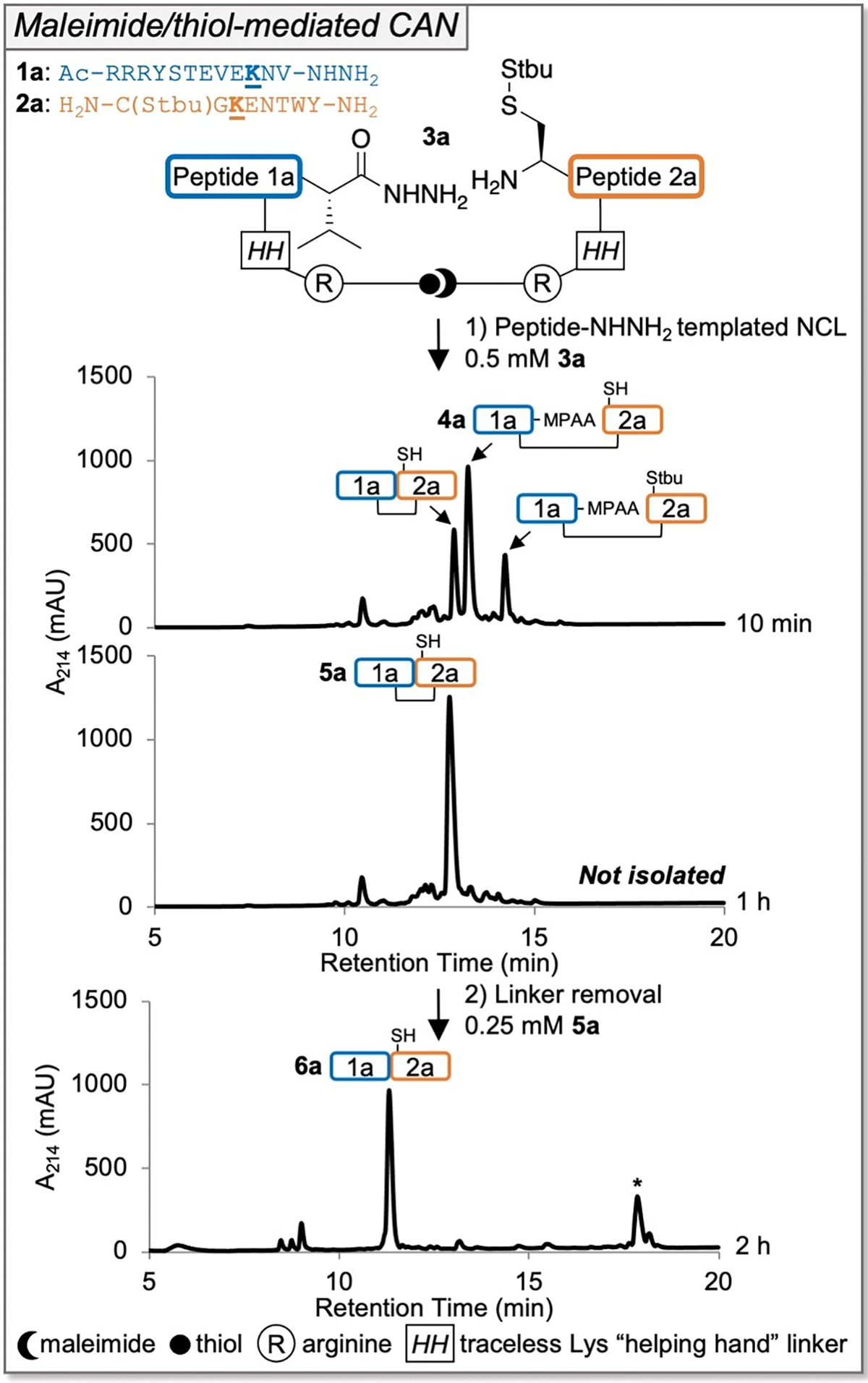
Maleimide/thiol-mediated CAN. Peptide **1a**-NHNH2 was functionalized with a thiol-containing HH linker and peptide **2a** with a maleimide-HH linker. Peptides **1a** and **2a** were conjugated to yield **3a** (2.0 mM **1a** and **2a**, 6 M GnHCl, pH 6.8, r.t. 10 min). CAN was initiated with the conversion to **4a** with subsequent ligation resulting in **5a** (0.5 mM **3a**, 15 eq NaNO_2_, 100 mM MPAA and TCEP, 6 M GnHCl, pH 6.8, r.t. 1 h). HH linker removal (0.25 mM **5a**, 1 M NH_2_OH, 6 M GnHCl, pH 6.8, r.t. 2 h) yielded the desired peptide **6a** demonstrating the utility of this second conjugation reaction needed for a three-segment system. The (*) indicates the cleaved HH linkers. Underlined and bolded Lys (**K**) indicate placements of HH linkers. Peptide modifications are indicated by symbols, with corresponding chemical groups detailed in the figure legend.

To unlock a three-segment templated NCL, a second conjugation reaction that is orthogonal to SPAAC used in CAN and compatible with SPPS/NCL needed to be identified. Although maleimide/thiol conjugation is not fully orthogonal to SPAAC (reactivity of thiols towards DBCO),^[28]^ we envisioned that sequential conjugation of peptides would overcome this drawback. From a SPPS perspective, maleimide/thiol seemed promising due to facile maleimide^[29]^ and thiol incorporation into peptides. Although encouraged by reports of maleimide use for CPS applications,^[30]^ we were concerned about possible conjugate instability during NCL as high concentrations (100 mM) of exogenous thiol (e.g., 4-mercaptophenylacetic acid; MPAA (pKa ~6.6)) and thiol at the ligation site could force conjugate dissociation at the thiosuccinimide via retro-Michael addition.^[31]^ Model peptides were synthesized with HH linkers functionalized with maleimide and thiol. To direct peptide conjugation to the HH linkers, the N-terminal Cys (required for NCL) was protected with Stbu, which is then easily removed by reducing agents in the ligation reaction. Following peptide cleavage from resin and RP-HPLC purification, peptides **1a** and **2a** were tethered together via maleimide/thiol conjugation in 10 min to yield **3a** (Figure S1–S2). Using analytical RP-HPLC and LC–MS, we monitored conjugate stability under NCL conditions (100 mM MPAA, 100 mM TCEP, 6 M GnHCl, pH 6.8) for 24 h. No detectable thiol exchange was identified over the time course of the experiment, suggesting that maleimide/thiol conjugation is well suited for templated NCL as reactions (including conjugation and ligation) typically do not exceed 4 h (Figure S3). To ensure compatibility with our CAN methodology, conjugate **3a** was activated in situ to **4a** by peptide-NHNH_2_ conversion to the thioester. CAN was completed in 1 h at 0.5 mM conjugate with efficient conversion to **5a**. One-pot traceless removal of the HH linkers was achieved in 2 h with hydroxylamine to afford the desired ligated peptide **6a**. Importantly, no complications with maleimide/thiol conjugation were identified (Figure 2 and S4).

### Three-Segment Templated NCL with Simultaneous Peptide-NHNH_2_ Activation

With a second conjugation method in hand, we set out to establish a reliable approach for modifying peptides with two distinct functionalized HH linkers to achieve selective conjugation of peptides for a three-segment templated NCL system. The internal peptide **2**-NHNH_2_ was synthesized using standard SPPS on hydrazone resin.^[32]^ This approach facilitated the strategy involving orthogonal 1-(4,4-dimethyl-2,6-dioxocyclohex-1-ylidene)-3-ethyl (Dde) and 4-methyltrityl (Mtt) amine protecting groups for the selective functionalization of HH linkers. To briefly outline the synthesis process, the initial step after peptide extension involved the removal of the Dde group using 5% hydrazine, allowing coupling of the 1^st^ HH linker, followed by the addition of Arg (for added solubility) and azide as previously described.^[20e]^ Subsequently, the Mtt group was removed with 2% TFA, enabling coupling of the 2^nd^ HH linker, which was then functionalized with Arg and thiol (Figure S5).

**Figure 3.**
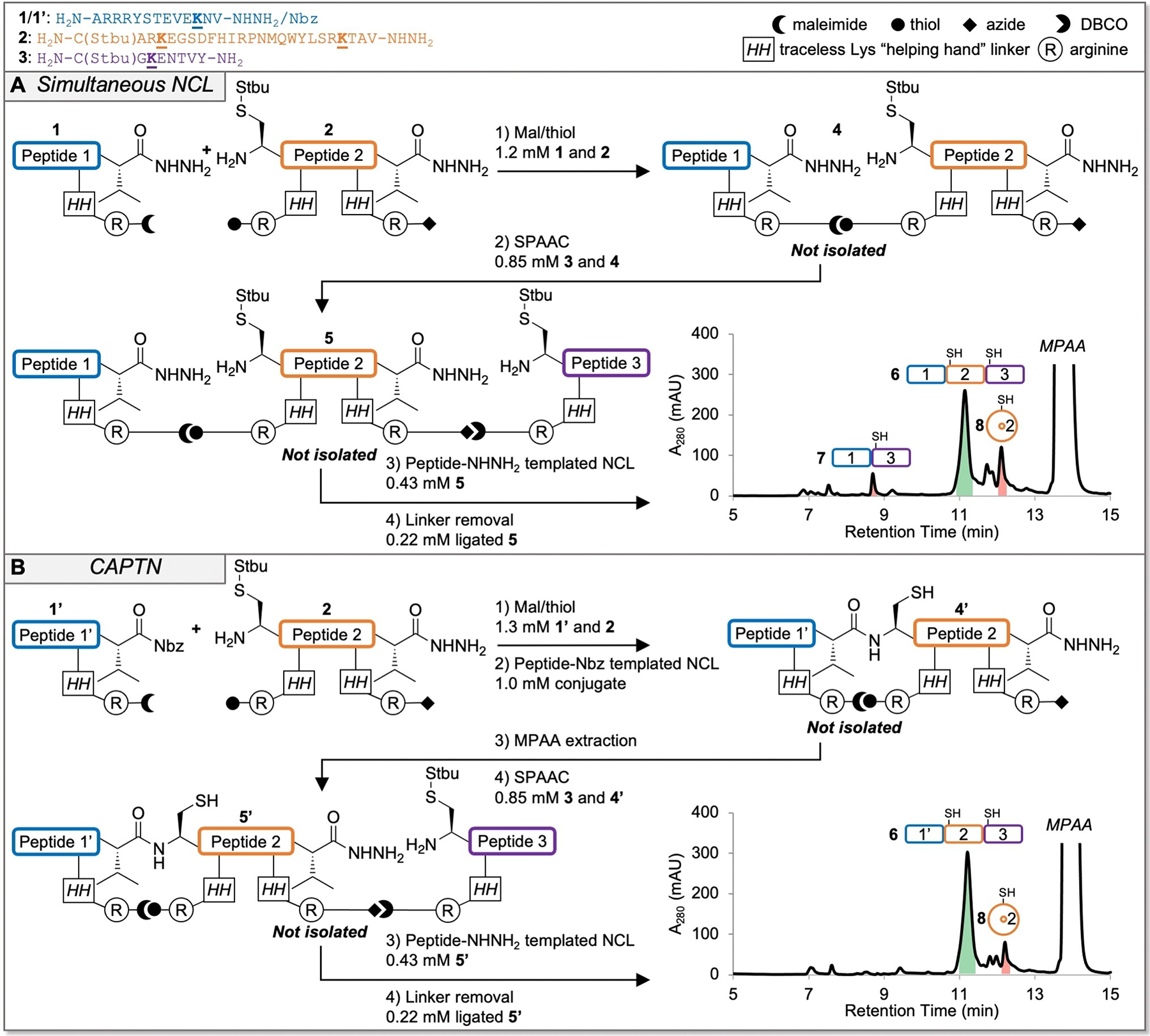
Three-segment templated NCL. (A) Three-segment templated NCL with simultaneous peptide-NHNH_2_ activation. Peptide **1**-NHNH_2_ was conjugated to peptide **2**-NHNH_2_ by maleimide/thiol-functionalized HH linkers to generate conjugate **4** (1.2 mM **1** and **2**, 6 M GnHCl, pH 6.8, r.t. 10 min). Peptide **3** was functionalized with a DBCO-containing HH linker and clicked to **4** via the azide-functionalized HH linker on peptide **2** (0.85 mM **3** and **4**, 6 M GnHCl, pH 6.8, r.t. 2 h). The resulting conjugate **5** underwent simultaneous templated NCL by peptide-NHNH_2_ **1** and **2** activation (0.43 mM **5**, 15 eq NaNO_2_, 100 mM MPAA and TCEP, 6 M GnHCl, pH 6.8, r.t. 3 h). Following removal of HH linkers (0.22 mM ligated **5**, 1 M NH_2_OH, 6 M GnHCl, pH 6.8, r.t. o/n), the desired product was obtained (**6**, green, 70% RP-HPLC yield), but cross-ligation between peptide **1** and **3** as well as cyclization of peptide **2** were observed (**7**, red, 6.5% and **8**, red, 18% RP-HPLC yield respectively). (B) Controlled Activation of Peptides for Templated NCL (CAPTN). Peptide **1’**-Nbz was functionalized with a maleimide-containing HH linker and conjugated to peptide **2-**NHNH_2_ via its thiol HH linker (1.3 mM **1’** and **2**, 6 M GnHCl, pH 6.8, r.t. 10 min). Peptide-Nbz templated NCL was performed to yield **4’** by selectively activating peptide **1’** (1.0 mM conjugate, 100 mM MPAA, 6 M GnHCl, pH 6.8, 37°C, 3 h). Following MPAA extraction by Et_2_O, conjugate **5’** was obtained by clicking peptide **3** to **4’** via the azide-functionalized HH linker on peptide **2** (0.85 mM **3** and **4’**, 6 M GnHCl, pH 3, r.t. 2 h). Peptide-NHNH_2_ templated NCL was performed by peptide **2** activation (0.43 mM **5’**, 15 eq NaNO_2_, 100 mM MPAA and TCEP, 6 M GnHCl, pH 6.8, r.t. 3 h). HH linker removal (0.22 mM ligated **5’**, 1 M NH_2_OH, 6 M GnHCl, pH 6.8, r.t. o/n) yielded the desired product (**6**, green, 87% RP-HPLC yield) with less peptide **2** cyclization (**8**, red, 6.6% RP-HPLC yield) and negligible cross-ligation between peptide **1’** and **3**. For (A) and (B), peptides **1** and **1’** have the same primary sequence but differ in their respective crypto-thioesters. Peptide **2** contains all canonical amino acids except for Met (substituted with Nle). Underlined and bolded Lys (**K**) indicate placements of HH linkers. Peptide modifications are indicated by symbols, with corresponding chemical groups detailed in the figure legend.

We first attempted a three-segment templated NCL with simultaneous peptide-NHNH_2_ activation. The internal peptide **2**-NHNH_2_ (containing thiol and azide HH linkers) was conjugated to peptide **1**-NHNH_2_ via maleimide/thiol (**4**) and then clicked to peptide **3** via SPAAC (**5**). The peptides of conjugate **5** were then ligated by simultaneous in situ thioester conversion of peptide **1**-NHNH_2_ and peptide **2-**NHNH_2_ via NaNO_2_ treatment followed by thiolysis with MPAA. TCEP was added to the reaction to remove the Stbu groups and the NCL ran for 3 h to guarantee completion. HH linkers were then cleaved by addition of hydroxylamine, all in one pot. The reaction resulted in the desired peptide **1**–**2**–**3** (**6**, 70% RP-HPLC yield), but cross-ligations occurred, yielding side products peptide **1**–**3** (**7**, 6.5% RP-HPLC yield) and cyclic peptide **2** (**8**, 18% RP-HPLC yield) (Figure 3A and S6–S9). These side reactions were anticipated and highlight the need for selective activation of peptide crypto-thioesters to drive formation of the full-length product.

### Three-Segment Templated NCL with Sequential Peptide Conjugation and Selective Thioester Activation

Inspired by kinetically controlled ligations introduced by the Kent group,^[33]^ we predicted that ligations could be similarly directed to the desired junctions using selective activation of peptide-thioester precursors. The Dawson method for preparing peptide thioesters via peptide-Nbz surrogates is attractive for several reasons, including robust SPPS, commercial availability of resins, and an established history in CPS.^[15,34]^ Because peptide-Nbz can be converted to a thioester in situ by MPAA addition alone, we predicted that selective activation would be achieved in the presence of a peptide-NHNH_2_, which requires oxidation by NaNO_2_, thus minimizing cyclization of the internal peptide. To completely avoid cross-ligation between peptide 1 and peptide 3, we expected that peptide 3 could be conjugated to the system after the 1^st^ ligation. To achieve this, three parameters needed to be considered:

1. Compatibility of the Dawson method with our templating strategies.
2. Survival of a click partner (i.e. thiol, maleimide, DBCO, or azide) during the 1^st^ NCL and one-pot conjugation of peptide 3.
3. One-pot peptide-NHNH_2_ activation following the 1^st^ NCL and subsequent conjugation reaction.

We first established that the peptide-Nbz approach was compatible with all steps involved in our two-segment CAN system, comprising functionalization of HH linkers with click groups by SPPS, on-resin conversion of Dbz linker (3,4-diaminobenzoyl) to Nbz, one-pot conjugation, ligation at fast and slow thioester junctions, and HH removal (Figure S10–S15).

When considering what click group would survive the 1^st^ NCL, we decided on using an alkyl azide for the following reasons:

1. An alkyl azide has been previously utilized as an N-terminal masking group during NCL.^[16b]^
2. Unconjugated maleimide would not survive NCL as it would react with MPAA.
3. Selective conjugation at the thiol HH linker would be problematic post-NCL since the now exposed Cys at the ligation junction would interfere with the conjugation.
4. DBCO reacts with thiols (especially at high MPAA concentrations).

A model peptide bearing an alkyl azide was treated with 100 mM MPAA to assess its stability under NCL conditions. No azide reduction was observed over 24 h, highlighting the suitability of this click group for one-pot NCL and SPAAC (Figure S16–S17). We typically rely on TCEP to remove the Stbu protecting group from the Cys at the ligation site, but no strong reducing agents can be used during the 1^st^ NCL as azide reduction would occur.^[16b]^ We anticipated that the mild reductive properties of MPAA would suffice to displace Stbu. A model peptide with an N-terminal Cys(Stbu) was treated with 100 mM MPAA to evaluate Stbu removal without TCEP and reached completion in 10 min (Figure S18). To confirm successful NCL in the absence of TCEP, we performed CAN (mediated by peptide-Nbz conversion) with and without TCEP. Although a slight decrease in initial ligation kinetics was observed in the reaction without TCEP (due to slower Stbu removal), both reactions reached completion in 2 h. Importantly, the mild reducing ability of MPAA (100 mM) was sufficient to completely remove Stbu within the first 30 min of the reaction suggesting that the 1^st^ NCL in the three-segment CAPTN system could be performed in the absence of TCEP to ensure azide survival (Figure S13–S14).

Since MPAA has an inhibitory effect on peptide-NHNH_2_ activation at high (100 mM) concentrations (Figure S20) and would also interfere with the SPAAC reaction, it must be removed prior to the 2^nd^ conjugation and subsequent NCL. Based on previous studies describing MPAA extraction with organic solvents,^[8c,35]^ we attempted peptide-NHNH_2_ activation following MPAA removal. Successful MPAA removal was achieved using both EtOAc and Et_2_O, but incomplete activation from the peptide-NHNH_2_ to the thioester was observed when using EtOAc while the Et_2_O resulted in complete conversion. We are uncertain why repetitive EtOAc extractions prevent activation. To our knowledge, this is the first demonstration of MPAA extraction for activation of peptide-NHNH_2_ thioester surrogates (Figure S21–S23). With a method established for selective activation of thioester precursors and one-pot sequential conjugation of peptides post-NCL, we then tested the CAPTN system. We conjugated peptide **1’**-Nbz (peptides **1** and **1’** differ solely in their respective crypto-thioesters) to peptide **2-**NHNH_2_ via maleimide and thiol HH linkers and performed the peptide-Nbz NCL by adding 100 mM MPAA to generate the ligated conjugate **4’**. The reaction was left for 3 h for comparison to the three-segment templated NCL experiment with simultaneous peptide-NHNH_2_ activation. Notably, no reduction of the azide-functionalized HH linker on peptide **2** was identified by LC–MS analysis. We then removed the MPAA using Et_2_O extractions without loss of **4’** and clicked peptide **3** via SPAAC (**5’**). The 2^nd^ ligation was initiated by NaNO_2_ oxidation and MPAA thiolysis of peptide **2**-NHNH_2_. The reaction ran for 3 h (again, for comparison) after which the HH linkers were removed, all in one pot. LC–MS analysis revealed conversion to the full-length ligated product peptide **1’**–**2**–**3** (**6**, 87% RP-HPLC yield) with minimal cyclization of the internal peptide **2** (**8**, 6.6% RP-HPLC yield) and negligible cross-ligation between peptide **1’** and **3** (1.0% RP-HPLC yield) (Figure 3B and S24–S30). CAPTN addresses the challenging side reactions in a three-segment one-pot templated NCL system by taking advantage of selective crypto-thioester activations and sequential conjugation of peptide segments.

### Accessing the E. coli 30S Ribosomal Subunits S16 and S17 by Templated NCL

**Figure 4.**
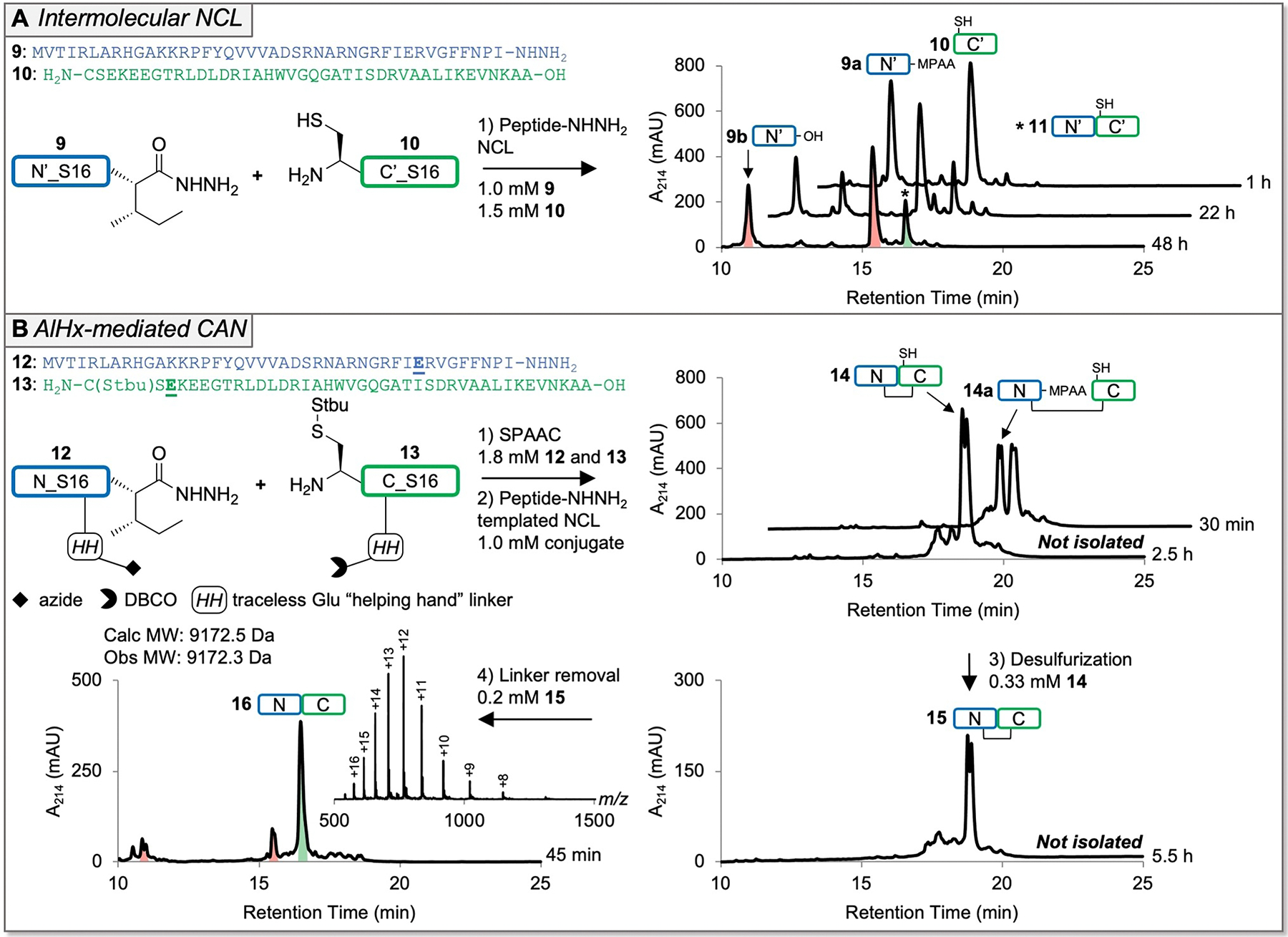
AlHx-mediated CAN of S16. (A) Intermolecular peptide-NHNH_2_ NCL of **9** with **10** (1.0 mM **9**, 1.5 mM **10**, 15 eq NaNO_2_, 100 mM MPAA, 20 mM TCEP, 6 M GnHCl, pH 6.8, r.t. 48 h). The ligation at the sluggish Ile thioester performed poorly, resulting in significant thioester hydrolysis **9b** (red) and unreacted **10** (red) as well as minimal product formation **11** (green, 20% RP-HPLC yield). (B) AlHx-mediated CAN of S16. Peptides **12** and **13** were clicked together via the azide-functionalized AlHx linker on **12** and the DBCO-functionalized AlHx linker on **13** (1.8 mM **12** and **13**, 6 M GnHCl, pH 6.8, r.t. 1 h). Two peaks are expected due to the formation of regioisomers.^[20e]^ Peptide-NHNH_2_ templated NCL was then accomplished by in situ thioester activation (**14a**) yielding ligated conjugate **14** (1.0 mM conjugate, 15 eq NaNO_2_, 100 mM MPAA, 100 mM TCEP, 6 M GnHCl, pH 6.8, r.t. 2.5 h). Importantly, minimal thioester hydrolysis was observed. Desulfurization to **15** was done post-dialysis and completed in 5.5 h (0.33 mM **14**, 60 mM VA-044, 120 mM GSH, 150 mM TCEP, 6 M GnHCl, pH 6.5, r.t. 5.5 h). Traceless cleavage of the AlHx linkers (0.2 mM **15**, 25 mM [Pd(allyl)Cl]_2_, 25 mM GSH, 6 M GnHCl, pH 8, 37°C, 45 min) followed by DTT quenching of the Pd (40 mM DTT, 6 M GnHCl, pH 7, r.t., 10 min) revealed full-length linear S16 (**16**, green, 81% RP-HPLC yield) in much cleaner and efficient one-pot manner, outperforming the intermolecular approach. Met in **9** and **12** was substituted with Nle. Underlined and bolded Glu (**E**) indicate placements of HH linkers. Peptide modifications are indicated by symbols, with corresponding chemical groups detailed in the figure legend.

The synthesis of a mirror-image ribosome capable of in vitro mirror-image translation is a long-standing goal in synthetic biology with exciting advancements in recent years including the synthesis of D-T7 RNA polymerase for the preparation of L-rRNAs.^[2g]^ The *E. coli* ribosome is comprised of 65 proteins (30S/50S subunits and accessory factors) varying in length and chemical complexity (e.g., occurrence of suitable NCL junctions) making it a compelling model system for assessing CPS tools. The S16 and S17 proteins caught our attention for templated NCL approaches as both proteins lack suitable ligation junctions with desirable expected kinetics. S16 contains an I42-A43 ligation site, dividing it into two peptide segments of favorable lengths for SPPS. Ile thioesters often suffer from slow ligation kinetics resulting in reduced product formation, but this can be overcome with templating. Although this retrosynthesis plan is only a two-segment approach, it provides an ideal system to test the use of our Glu(AlHx) traceless HH linker for templating as Glu residues flanking the ligation junctions are better positioned (closer to NCL junction) than Lys residues. We anticipated that this extra attachment point would alleviate the Lys residue restriction imposed by CAN. In addition, this demonstration ensured that no unexpected reactions are observed with a two-segment templated approach, suggesting translatability to the CAPTN method. We first attempted the synthesis of S16 using traditional intermolecular NCL via peptide-NHNH_2_ activation. The N-terminal half of S16 (42 amino acids) was prepared by SPPS on CTC-hydrazide resin to yield peptide **9**-NHNH_2_ (N’_S16,’ indicating peptides for an intermolecular NCL approach). The C-terminal half (40 amino acids) was prepared by SPPS as an acid peptide on CTC resin yielding peptide **10** (C’_S16). Both peptides were purified by RP-HPLC and the NCL was conducted at 1.0 mM **9** and 1.5 mM **10** following peptide-NHNH_2_ activation to **9a**. Significant thioester hydrolysis (**9b**) was observed over 48 h, resulting in unreacted **10** and minimal ligated product **11** (20% RP-HPLC yield) (Figure 4A and S31–S32). To overcome this poor conversion, we templated the ligation using Glu(AlHx) linkers at positions E34 on peptide **12**-NHNH_2_ (N_S16, functionalized with azide) and E45 on peptide **13** (C_S16, functionalized with DBCO). After preparative RP-HPLC, the peptides were clicked together in 1 h at 1.8 mM. CAN was initiated in one pot post-SPAAC by peptide **12**-NHNH_2_ activation (**14a**), and the ligation reached completion in 2.5 h yielding conjugate **14**. Similar conversion was observed at 1.0 mM and 0.1 mM peptide conjugate, again demonstrating the ability of templated reactions to perform at sub-mM concentrations.^[20e]^ Importantly, product formation was much greater than the intermolecular NCL approach with a minor amount of thioester hydrolysis. MPAA was removed by dialysis to facilitate desulfurization of C43 to A43 (**15**) followed by removal of the AlHx linkers to yield native linear S16 (**16**, 81% RP-HPLC yield) (Figure 4B and S33–S36).

**Figure 5.**
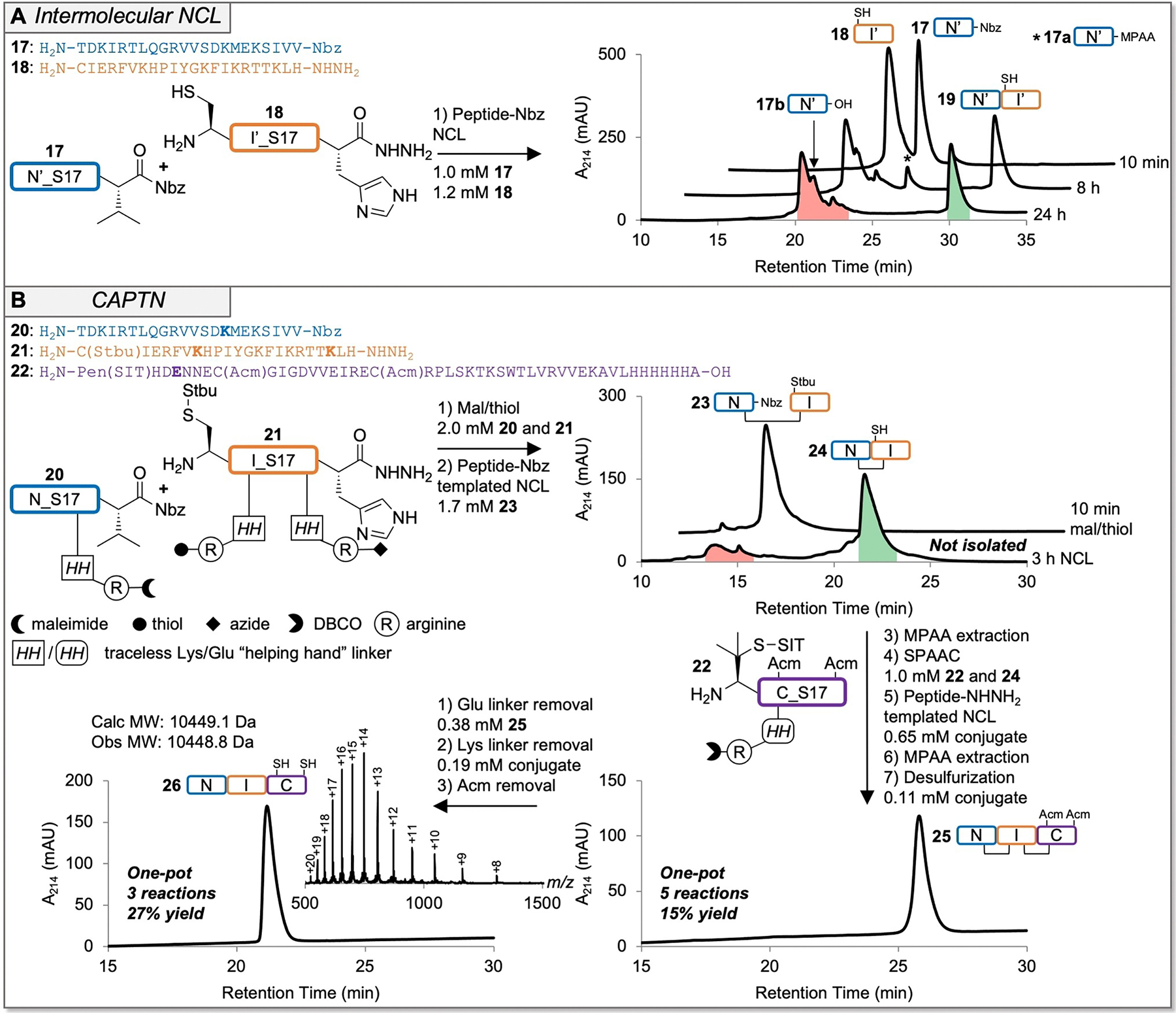
CAPTN-mediated synthesis of S17. (A) Intermolecular peptide-Nbz NCL of **17** with **18** (1.0 mM **17**, 1.2 mM **18**, 100 mM MPAA, 20 mM TCEP, 6 M GnHCl, pH 6.8, 37°C, 24 h). The ligation at the sluggish Val thioester resulted in thioester hydrolysis **17b** (red) and unreacted **18** (red) with 50% product formation **19** (green, RP-HPLC yield). (B) CAPTN-mediated synthesis of S17. Peptide **20**-Nbz was functionalized with a maleimide-containing HH linker and conjugated to peptide **21**-NHNH_2_ via its thiol HH linker (2.0 mM **20** and **21**, 6 M GnHCl, pH 6.8, r.t. 10 min). Peptide-Nbz templated NCL was performed on conjugate **23** by selective peptide-Nbz activation (1.7 mM **23**, 100 mM MPAA, 6 M GnHCl, pH 6.8, 37°C, 3 h) and yielding the desired ligated conjugate **24** (green). Following MPAA extraction by Et_2_O, **22** was clicked to **24** via the azide-functionalized HH linker on **21** (1.0 mM **22** and **24**, 6 M GnHCl, pH 3, r.t. 2 h). Peptide-NHNH_2_ templated NCL was performed resulting in full-length ligated S17 conjugate (0.65 mM conjugate, 15 eq NaNO_2_, 100 mM MPAA and TCEP, 6 M GnHCl, pH 6.8, r.t. 4 h). Desulfurization was conducted in one pot by first extracting MPAA with Et_2_O, then initiating with VA-044 (0.11 mM conjugate, 60 mM VA-044, 120 mM GSH, 300 mM TCEP, 6 M GnHCl, pH 6.5, 60°C, 4 h). The reaction was then purified by RP-HPLC to isolate the desulfurized S17 conjugate (**25**) with 15% isolated yield over five reactions done in one pot. AlHx removal was achieved in 4 h (0.38 mM **25**, 20 mM Pd/TPPTS, 10 mM GSH, 6 M GnHCl, pH 8, 37°C, 4 h) followed by Ddap removal in 2 h (0.19 mM conjugate, 1 M NH_2_OH, 6 M GnHCl, pH 6.8, r.t. 2 h). Dialysis and PdCl_2_ treatment (5.0 mM PdCl_2_, 6 M GnHCl, pH 7, r.t., 30 min) with subsequent DTT quenching (250 mM DTT, 6 M GnHCl, pH 7, r.t., 10 min) removed remaining Acm yielding linear S17 (**26**) that was purified by RP-HPLC with 27% isolated yield over the last three reactions. Met in **17** and **20** was substituted with Nle. Underlined and bolded Lys (**K**) and Glu (**E**) indicate placements of HH linkers. Peptide modifications are indicated by symbols, with corresponding chemical groups detailed in the figure legend.

The initial retrosynthesis plan for S17 was to split the protein into two segments at the H45 and V46 junction which we thought could be accessed via kinetically enhanced templated ligation at Pen. As demonstrated with model peptides, a templated Pen ligation outperforms the corresponding intermolecular reaction significantly (Figure S37–S41) and the H45-V46 junction for S17 is surrounded by ideally placed K43 (on the N-terminal peptide) and E49 (on the C-terminal peptide) for linker incorporation. Unfortunately, SPPS failed to produce the N-terminal peptide segment even after extensive troubleshooting including incorporation of pseudoprolines. Alternative synthetic approaches, such as a two-segment system with a V23-A24 ligation junction, also failed to yield appropriate peptides (Figure S42). We therefore redesigned the retrosynthesis plan splitting S17 into three segments with the additional ligation junction V23-A24 and the original H45-V46. This strategy breaks up the problematic N-terminal half at the only suitable site, resulting in two 22-residue peptide segments. We attempted the intermolecular NCL between peptide **17**-Nbz (N’_S17, 1.0 mM) and peptide **18**-NHNH_2_ (I’_S17, internal peptide, 1.2 mM) by selective peptide-Nbz activation (**17a**) which yielded the desired N-terminal half of S17 (**19**, 50% RP-HPLC yield), but with significant thioester hydrolysis (**17b**) (Figure 5A and S43–S44). Aware that the subsequent ligation with Pen would likely prove even more difficult due to the steric bulk surrounding the thiol, we hypothesized that CAPTN would overcome the expected slow ligation kinetics and thioester hydrolysis at both junctions and provide an ideal case study to demonstrate a one-pot three-segment templated NCL system. In addition, this is our first report of using both Ddap (Lys HH) and AlHx (Glu HH) linkers on the same protein, allowing us to assess their compatibility for a one-pot removal. Finally, S17 contains two native Cys residues that are not optimal for NCL, due to their respective Glu thioesters being prone to side reactions that require extensive monitoring,^[36]^ as well as poor positioning of the Cys nearest the C-terminus. We therefore protected both Cys with acetamidomethyl (Acm), enabling us to assess this frequently used protecting group with our chemical tools; specifically simultaneous removal during Pd-mediated AlHx cleavage. All three S17 peptides segments were accessible by SPPS and were prepared as described earlier to yield peptide **20**-Nbz (N_S17) with a maleimide-Ddap linker at K16, the internal peptide as peptide **21**-NHNH_2_ (I_S17) with two differently functionalized Ddap linkers (thiol at K30 and azide at K43), and peptide **22** (C_S17) with a DBCO-modified AlHx linker at E49. While Stbu has been our primary Cys protecting group for our templated methods, we were wary of potential slow removal on Pen of **22** due to the added steric hindrance. Inspired by a recent report of sec-isoamyl mercaptan (SIT) as a thiol protecting group with improved removal kinetics,^[37]^ we prepared model peptides bearing both Pen(Stbu) and Pen(SIT) via the commercially available Pen(3-nitro-2-pyridinesulfenyl; Npys). We then attempted reduction with 100 mM TCEP which quickly yielded the fully unprotected Pen peptide in less than 10 min when protected with SIT while minimal removal was seen with Stbu over the course of 2 h. Peptide **22** was thus synthesized with Pen(SIT) (Figure S45–S48).

The CAPTN assembly of S17 began with the maleimide/thiol conjugation reaction tethering **20** with **21** at 2.0 mM to generate conjugate **23**. Peptide-Nbz templated NCL at 1.7 mM yielded ligated conjugate **24** with minimal thioester hydrolysis. The MPAA was removed with Et_2_O extractions to enable the subsequent SPAAC with **22** at 1.0 mM. The peptide-NHNH_2_ templated NCL was then initiated by NaNO_2_ oxidation and thiolysis with MPAA at 0.65 mM S17 conjugate. Although modest thioester hydrolysis occurred, the major product was full-length ligated S17 conjugate (Figure S49–S53). We continued with an additional Et_2_O-mediated MPAA extraction to enable one-pot desulfurization with VA-044 radical initiation. The initial desulfurization attempts suffered from stalling due to VA-044 adducts (previously reported by others),^[38]^ and while this issue was overcome by elevating the TCEP concentration to 300 mM, the desulfurization accumulated unwanted byproducts over time. We settled on a 4 h reaction at 60°C to aid the sluggish desulfurization at Pen while minimizing side reactions. The desired desulfurized S17 conjugate (**25**) was purified by RP-HPLC with a 15% isolated yield over five reactions performed all in one pot. (**S54**–**S56**). Full-length S17 was attained by removing the AlHx linker with Pd/TPPTS, followed by removal of the three Ddap linkers via hydroxylamine treatment. The reactions proceeded smoothly, but only partial Acm removal was observed. We achieved full Acm deprotection by adding PdCl_2_ following dialysis. DTT was then added to quench the Pd and linear S17 (**26**) was purified with a 27% isolated yield over the last three steps, all done in a one-pot manner (Figure 5B and S57).

### Conclusion

In this work, we report the development of a one-pot three-segment templated ligation system, CAPTN, that takes advantage of selective activation of thioester precursors and sequential peptide conjugation. This approach minimizes undesired cross-ligation and peptide cyclization reactions that are expected when performing one-pot templated ligations. We expanded on our CAN methodology that uses SPAAC for templated NCL by combining it with maleimide/thiol conjugation. We demonstrated that the succinimide linkage is stable and well suited for NCL purposes. This extra conjugation reaction enables the tethering of three peptides together in a sequential fashion without intermediate purifications. CAPTN utilizes robust and well-established chemistries, including peptide-Nbz and peptide-NHNH_2_ crypto-thioesters, to control the NCL at the desired junction with selective activation of peptides in a straightforward manner. Importantly, the one-pot peptide-NHNH_2_ activation following the first NCL was accomplished by MPAA removal; potentially useful for many CPS projects. To further expand our templating capabilities, we determined compatibility of our AlHx traceless linker with the synthesis of the *E. coli* 30S ribosomal subunit S16; providing coverage over a wider range of peptide sequences. While both sluggish thioesters and Pen junctions are accessible with the templating tools described here, which will greatly help CPS endeavors, we expect that templating will also assist in the ligations of peptides at anticipated favorable junctions that underperformed in intermolecular NCL (e.g., due to suboptimal concentrations from poor peptide solubility).

We demonstrated the robustness of CAPTN with the chemical synthesis of the *E. coli* 30S ribosomal subunit S17, which required three segments and ligations at two unfavorable junctions (Val thioester and Pen). Moreover, we established the convenient protection of the Pen thiol using SIT, which is quickly removed with TCEP and greatly outperforms Stbu. Finally, S17 required the use of both our traceless linkers, Ddap and AlHx, which we showed can be detached in one pot in combination with Acm removal.

The efficiency of our demonstrations suggests CAPTN will be useful to access sections of larger proteins that need templated assistance due to poor NCL yields. Once the difficult region is obtained via CAPTN, extension in the N-to-C direction could be achieved using peptide salicylaldehyde *S,S*-propanedithioacetal-ester compatible with sequential ligations.^[39]^ C-to-N extension would be possible with N-terminal masking or thiol protection stable to CAPTN conditions (e.g., Tfa-Thz^[40]^ or Acm). Such efforts are ongoing in our laboratory. Additionally, CAPTN expands on existing templated methodologies and will ideally lead to further developments for the rapid assembly of more complex proteins. Finally, CAPTN provides a blueprint for properly considering chemical compatibilities when using routinely employed conjugation (maleimide/thiol and SPAAC) with NCL methods (via peptide-Nbz and peptide-NHNH_2_) that we expect will have broad applications beyond CPS.

## Supplementary Material

supplemental information

## Data Availability

The data that support the findings of this study are available in the supplementary material of this article.
