# Supplementary material for: Selective Activation of Peptide-Thioester Precursors for Templated Native Chemical Ligations
Source: Angew Chem Int Ed Engl. Author manuscript; Available in PMC 2025 Mar 17. (PMC11913120; doi:10.1002/anie.202413644)
Supplement: supplemental information [file NIHMS2058785-supplement-supplemental_information.pdf]

Angewandte Chemie
Eine Zeitschrift der Gesellschaft Deutscher Chemiker

Supporting Information

Selective Activation of Peptide-Thioester Precursors for Templated Native Chemical Ligations

*P. Spaltenstein, R. J. Giesler, S. R. Scherer, P. W. Erickson, M. S. Kay**

Supporting Information
©Wiley-VCH 2021
69451 Weinheim, Germany

Selective Activation of Peptide-Thioester Precursors for Templated Native Chemical Ligations

Paul Spaltenstein, Riley J. Giesler, Samuel R. Scherer, Patrick W. Erickson, Michael S. Kay

Abstract: Chemical protein synthesis enables access to proteins that would otherwise be difficult or impossible to obtain with traditional means such as recombinant expression. Chemoselective ligations provide the ability to join peptide segments prepared by solid-phase peptide synthesis. While native chemical ligation (NCL) is widely used, it is limited by the need for C-terminal thioesters with suitable reaction kinetics, properly placed native Cys or thiolated derivatives, and peptide segment solubility at low mM concentrations. Moreover, repetitive purifications to isolate ligated products are often yield-sapping, hampering efficiency and progress. In this work, we demonstrate the use of Controlled Activation of Peptides for Templated NCL (CAPTN). This traceless multi-segment templated NCL approach permits the one-pot synthesis of proteins by harnessing selective thioester activation and orthogonal conjugation chemistries to favor formation of full-length ligated product while minimizing side reactions. Importantly, CAPTN provides kinetic enhancements allowing ligations at sterically hindered junctions and low peptide concentrations. Additionally, this one-pot approach removes the need for intermediate purification. We report the synthesis of two *E.coli* ribosomal subunits S16 and S17 enabled by the chemical tools described herein. We anticipate that CAPTN will expedite the synthesis of valuable proteins and expand on templated approaches for chemical protein synthesis.

Table of Contents

Sections	Pages
1) Materials	2-3
2) Experimental Procedures	3-17
3) Supplementary Table	17-18
4) Supplementary Figures and Schemes	18-63
5) References	63-64

SECTION 1: Materials

Solid-Phase Peptide Synthesis (SPPS)

Tentagel R RAM resin (0.19 mmol/g) was purchased from Rapp Polymere. 2-chlorotrityl chloride resin (0.77 mmol/g) was purchased from ChemPep. Fmoc-NHN=Pys resin (>0.3 mmol/g) was purchased from Iris Biotech GmbH. 3-(Fmoc-amino)-4-aminobenzoyl AM resin (Dawson resin, 0.43 mmol/g), triethylamine (TEA), 4-nitrophenyl chloroformate, and 2-methyl-2-propanethiol (t-BuSH, 99%) were purchased from MilliporeSigma, Fmoc-L-Ala-OH, Fmoc-L-Cys(Trt)-OH, Fmoc-L-Asp(OtBu)-OH, Fmoc-L-Glu(OtBu)-OH, Fmoc-L-Phe-OH, Fmoc-Gly-OH, Fmoc-L-His(Trt)-OH, Fmoc-L-Ile-OH, Fmoc-L-Lys(Boc)-OH, Fmoc-L-Leu-OH, Fmoc-L-Asn(Trt)-OH, Fmoc-L-Pro-OH, Fmoc-L-Gln(Trt)-OH, Fmoc-L-Arg(Pbf)-OH, Fmoc-L-Ser(tBu)-OH, Fmoc-L-Thr(tBu)-OH, Fmoc-L-Val-OH, Fmoc-L-Trp(Boc)-OH, and Fmoc-L-Tyr(tBu)-OH were purchased from Gyros Protein Technologies. Fmoc-Gly-Ser($\Psi^{Me,Me}$pro)-OH, Fmoc-Leu-Ser($\Psi^{Me,Me}$pro)-OH, Fmoc-Val-Thr($\Psi^{Me,Me}$pro)-OH, Fmoc-Gly-Thr($\Psi^{Me,Me}$pro)-OH, Fmoc-Ala-Thr($\Psi^{Me,Me}$pro)-OH, Fmoc-Val-Ser($\Psi^{Me,Me}$pro)-OH, Fmoc-Lys(Boc)-Ser($\Psi^{Me,Me}$pro)-OH, Fmoc-Trp(Boc)-Thr($\Psi^{Me,Me}$pro)-OH, Fmoc-L-Lys(Dde)-OH, Ac-L-Cys(Trt)-OH and Fmoc-L-Norleucine-OH were purchased from AAPPTec. Boc-L-Cys(Stbu)-OH, Boc-Pen(Trt)-OH, and 6-azidohexanoic acid (≥98%) were purchased from Chem-Impex International. Fmoc-L-Lys(Mtt)-OH, acetic anhydride (99.5%), di-tert-butyl dicarbonate (ReagentPlus grade), anhydrous hydrazine (98%), *N,N*-diisopropylethylamine (DIPEA, ReagentPlus grade), piperidine (ReagentPlus grade), *N*-methylmorpholine (NMM, ReagentPlus grade), 6-maleimidohexanoic acid, and Boc-Ala-OH were purchased from Sigma Aldrich. Fmoc-hydrazine (98%), dimethylformamide (DMF, ACS grade), dichloromethane (DCM, ACS grade), N-methylpyrrolidone (NMP, ≥99.8%), methanol (MeOH, ACS grade), and 3-methyl-2-butanethiol (SIT, >98%) were purchased from Fisher Scientific. 1-[Bis(dimethylamino)methylene]-1*H*-1,2,3-triazolo[4,5-*b*]pyridinium 3-oxid hexafluorophosphate (HATU, 99%) was purchased from Oakwood Chemical. Ninhydrin (ACS grade) was purchased from Acros Organics. 190 proof ethanol and 200 proof ethanol were purchased from Decon Laboratories. DBCO-C6-NHS ester (>95%) was purchased from Click Chemistry Tools. Boc-Pen(NPys)-OH was purchased from Bachem. The traceless Lys linker *N*-Fmoc-2-(7-amino-1-hydroxyheptylidene)-5,5-dimethylcyclohexane-1,3-dione (Fmoc-Ddap-OH) and Glu linker Fmoc-Glu((*E*)-4-hydroxybut-2-en-1-yl 6-((1-(Dde)amino)hexanoate))-OH (Fmoc-Glu(AlHx)-OH) were synthesized as described in the established protocols.[1]

Peptide Cleavage and Precipitation

Trifluoroacetic acid (TFA, peptide synthesis grade) and anhydrous ethyl ether (ACS grade) were purchased from Fisher Scientific. Tetrakis(acetonitrile)copper(I) tetrafluoroborate ($[MeCN]_4CuBF_4$, 97%) and triisopropylsilane (TIS, 98%) were purchased from Sigma Aldrich. 1,2-ethanedithiol (EDT, 95%) was purchased from Acros Organics. Thioanisole (≥99%) was purchased from Fluka.

RP-HPLC and LC-MS

Trifluoroacetic acid (TFA, HPLC grade) was purchased from Alfa Aesar. Acetonitrile (MeCN, HPLC grade), 0.1% formic acid in water (Optima LC-MS grade), and 0.1% formic acid in acetonitrile (Optima LC-MS grade) were purchased from Fisher Scientific.

Click, Ligation, Desulfurization, Ddap/AlHx Linker Removal, and Acm Removal

Guanidine hydrochloride (GnHCl, ≥99.5%) was purchased from Thermo Scientific. Sodium phosphate dibasic heptahydrate (ACS grade) was purchased from AMRESCO. Sodium phosphate monobasic (ACS grade), hydrochloric acid (HCl, ACS plus grade), 4-mercaptophenylacetic acid (MPAA, 97%), and sodium nitrite ($NaNO_2$, ACS grade) were purchased from Fisher Scientific. Sodium hydroxide (NaOH, ACS grade), hydrochloric acid (HCl, ACS grade), and acetic acid (>99.8%) were purchased from Acros Organics. Tris (2-carboxyethyl) phosphine hydrochloride (TCEP-HCl, ≥99%) was purchased from Hampton Research. VA-044 was purchased from Wako. L-glutathione reduced (GSH, ≥98%), hydroxylamine hydrochloride (NH_2OH, ReagentPlus grade), palladium(II) acetate ($Pd(OAc)_2$, 98%), allylpalladium(II) chloride dimer ($[Pd(allyl)Cl]_2$, 98%), palladium(II) chloride ($PdCl_2$, ≥99.9%), and triphenylphosphine-3,3',3"-trisulfonic acid trisodium salt (TPPTS, ≥95%) were purchased from Sigma Aldrich. Dithiothreitol (DTT, ≥99%) was purchased from Gold Biotechnology.

SECTION 2: Experimental Procedures

Automated Peptide Synthesis

All peptides were synthesized on Prelude X or PurePep Chorus instruments (Gyros Protein Technologies) using Fmoc SPPS. All deprotection and coupling cycles were performed with nitrogen bubbling, and gentle shaking was also used for coupling steps. All cycles were performed at room temperature (r.t.) unless otherwise specified.

For 25 µmol scale syntheses, deprotection cycles consisted of two consecutive 3 min treatments of 2 mL 20% PIP in DMF, followed by three 30 s washes of 2 mL DMF. Coupling cycles consisted of 25 min treatment with a solution of 0.65 mL 200 mM amino acid in DMF, 0.65 mL 195 mM HATU in DMF, and 0.5 mL 600 mM NMM in DMF, followed by three 30 s washes of 2 mL DMF.

For 50 µmol scale syntheses, deprotection cycles consisted of two consecutive 3 min treatments of 4 mL 20% PIP in DMF, followed by three 30 s washes of 4 mL DMF. Coupling cycles consisted of 25 min treatment with a solution of 1.4 mL 200 mM amino acid in DMF, 1.4 mL 195 mM HATU in DMF, and 1 mL 600 mM NMM in DMF, followed by three 30 s washes of 4 mL DMF.

Washing, Swelling, and Mixing of Resins

For 25 µmol scale syntheses, resins were washed and swelled with approximately 3 mL of specified solvent. The wash volumes for all other synthesis scales are indicated in the relevant sections below. All swelling and manual coupling/deprotection steps were mixed on a rotisserie at r.t., unless otherwise specified.

Preparation of Peptide Resins

To synthesize **C-terminal amide** peptides at 25 µmol scale, 132 mg Tentagel R RAM resin (0.19 mmol/g) was weighed into a 6 mL SPPS tube. The resin was washed three times with DMF, followed by three DCM washes. The resin was then allowed to swell in

DMF for ≥ 10 min, and the swelled resin was transferred to the instrument for automated SPPS. The same protocol was followed for 50 μmol scale resin preparation with amounts (mg and mL) adjusted accordingly.

To prepare **C-terminal acid** peptides at 25 μmol scale, 150 mg 2-chlorotrityl chloride resin (0.77 mmol/g) was weighed into a 6 mL SPPS tube. The resin was washed three times with DMF, followed by three DCM washes. In order to load the first amino acid onto the resin, 0.03 mmol of the Fmoc-protected amino acid was dissolved in 1 mL of a 1:1 DMF/DCM mixture. 0.15 mmol DIPEA (26 μL) was then mixed into the amino acid solution. The entire amino acid and DIPEA solution was added to the 2-chlorotrityl chloride resin, and the coupling reaction was rotated for 1 h. The resin was then washed three times with DCM. Unreacted 2-chlorotrityl chloride was capped by repeatedly washing the resin with a 17:2:1 DCM:MeOH:DIPEA mixture (~20 mL total used). Once the capping reaction was complete, the resin was washed three times with DCM, followed by three DMF washes. Finally, the resin was transferred to the instrument for automated SPPS. This procedure generates resin with a loading density similar to Tentagel R RAM resin (approximately 0.2 mmol/g). See "*Determining Resin Loading Density using Fmoc Absorbance"* for more on loading density. The same protocol was followed for 50 μmol scale resin preparation with amounts (mg and mL) adjusted accordingly.

To synthesize **C-terminal peptide-NHNH$_2$** at 25 μmol scale, Fmoc-hydrazine on 2-chlorotrityl chloride resin was prepared based on an established protocol,[2] with several modifications. 150 mg of 2-chlorotrityl chloride resin (0.77 mmol/g) was weighed into a 6 mL SPPS tube. The resin was washed three times with DCM. 1.2 mL DCM was then added to the resin, and the resin was allowed to swell for 10 min at 4 °C. 30.6 μmol Fmoc-hydrazine (7.8 mg) was dissolved in a mixture of 1.5 mL DMF and 0.3 mL DCM, followed by addition of 266 μL DIPEA. Once the 2-chlorotrityl chloride resin was finished swelling, the Fmoc-hydrazine and DIPEA solution was added to the resin slurry at 4 °C. The resin slurry was then placed on a rotisserie at r.t., and the coupling reaction was rotated for 1 h. Once the coupling reaction was finished, 30 μL MeOH was added to the resin (without draining the SPPS tube) in order to cap unreacted 2-chlorotrityl chloride (rotated for 10 min). The resin was then washed three times with DMF, followed by three DCM washes. The resin was then swelled in a 1:1 DMF/DCM mixture for ≥ 10 min and transferred to the instrument for automated SPPS. This procedure generates resin with a loading density similar to Tentagel R RAM resin (approximately 0.2 mmol/g). See "*Determining Resin Loading Density using Fmoc Absorbance"* for more on loading density. The same protocol was followed for 50 μmol scale resin preparation with amounts (mg and mL) adjusted accordingly.

To synthesize **C-terminal peptide-NHNH$_2$** peptides at 25 μmol scale for use in double HH linker functionalization via Lys(Dde) and Lys(Mtt), the resin was prepared based on an established protocol with some modifications.[3] 122 mg of Fmoc-hydrazone resin (Fmoc-NHN=Pys resin, 0.82 mmol/g) was weighed into a 15 mL SPPS tube. The resin was washed three times with DMF. The resin was allowed to swell for 30 min at r.t. in 1:1 DMF/DCM mixture. Fmoc removal was completed with three consecutive 10 min treatments of 6 mL 20% PIP in DMF, with mixing. The resin was then washed three times with DMF. The first Fmoc-AA was manually coupled onto the resin by adding 5.6 mL 300 mM Fmoc-AA in DMF, 2 mL 0.5 M Oxyma in DMF, and 2 mL 1 M DIC in DMF. The coupling reaction ran overnight (~12 hr) at r.t. The resin was then washed three times with DMF and then capped with 4 mL acetic anhydride and 4 mL 0.6 M NMM in DMF for 20 min with mixing. The resin was washed three times with DMF and the resin loading density was determined following the described protocol *"Determining Resin Loading Density using Fmoc Absorbance"* and cut in half if needed following *"Lowering Resin Loading Density with Boc-Ala".* The resin was transferred to the instrument for automated SPPS. The same protocol was followed for 50 μmol scale resin preparation with amounts (mg and mL) adjusted accordingly.

To synthesize **C-terminal peptide-Nbz** at 25 μmol scale, 116 mg of Dawson resin (0.43 mmol/g) was weighed into a 6 mL SPPS tube. The resin was washed three times with DMF. The resin was allowed to swell for 30 min at r.t. in 1:1 DMF/DCM mixture. Fmoc was manually deprotected through two consecutive 3 min treatments of 4 mL 20% PIP in DMF, with mixing. After the second treatment, the resin was washed three times with DMF. The resin loading density was cut in half (~0.2 mmol/g) following the described protocol *"Lowering Resin Loading Density with Boc-Ala"* and transferred to the instrument for automated SPPS. Note that we recommend using the second generation Dawson resin (MeDbz) if synthesizing glycine-rich sequences.[4] The same protocol was followed for 50 μmol scale resin preparation with amounts (mg and mL) adjusted accordingly.

Determining Resin Loading Density using Fmoc Absorbance

The resin loading density was determined by Fmoc removal and Fmoc absorbance. To do so, the resin (25 μmol scale) was treated with 4 mL of 20% PIP in DMF with mixing for 20 min. The PIP-Fmoc solution was collected and diluted 100x with 20% PIP in DMF. Triplicate Fmoc absorbance measurements were taken at 280 nm using a NanoDrop One^{C} instrument (Thermo Scientific) blanked with 20% PIP in DMF. The average A_{280} was used to calculate the Fmoc concentration using an A_{280} extinction coefficient of 5680 $M^{-1}cm^{-1}$. From there, the loading density can be calculated as mmol/g.

Lowering Resin Loading Density with Boc-Ala

In case the resin density was too high, the following AA coupling was conducted in the presence of Boc-Ala to cut the loading density in half. To do so, 200 mM Boc-Ala in DMF was mixed with 200 mM Fmoc-AA in DMF (next residue to be coupled onto the peptide) to a ratio of ¼ Boc-Ala and ¾ Fmoc-AA. Then, following standard Fmoc removal, standard amino acid coupling was performed with the Boc-Ala/Fmoc-AA mixture. While this approach works well to cut the loading density in half with most Fmoc-AA, we recommend checking the loading density and altering the Boc-Ala/Fmoc-AA ratio as needed.

N-terminal Capping Procedures

N-terminal peptide acetylation at 25 μmol scale was achieved through 20 min treatment of 1 mL acetic anhydride combined with 1 mL 600 mM NMM in DMF, with mixing. The resin was then washed three times with DMF, followed by three DCM washes. The same protocol was followed for a 50 μmol scale but amounts (mL) were adjusted accordingly.

N-terminal Boc protection at 25 μmol scale was completed through two consecutive 20 min treatments of 0.7 mL 200 mM di-tert-butyl dicarbonate in DMF mixed with 0.5 mL 600 mM NMM in DMF, with mixing. Following the Boc treatments, the resin was washed three times with DMF, followed by three DCM washes. The same protocol was followed for a 50 μmol scale but amounts (mL) were adjusted accordingly.

Manual Peptide Synthesis of Peptide with One Functionalized Lys HH

In order to couple various modifications on a specific Lys residue (e.g., Ddap linker, DBCO, maleimide, thiol, or azide), manual couplings/deprotections were performed on peptides containing a single Lys(Dde) after automated peptide synthesis and N-terminal capping was completed. Manual peptide syntheses were generally completed at either 25 or 50 μmol scale as previously reported.[5]

Manual Lys(Dde) Deprotection

For 25 μmol scale Lys(Dde) deprotection, peptide resin was treated with 2 mL 5% hydrazine in DMF for three consecutive 5 min treatments, with mixing. Following the hydrazine treatment, the resin was washed six times with DMF.

Manual Fmoc-Ddap-OH Coupling

Coupling of the Lys helping hand linker (Fmoc-Ddap-OH) was completed based on the established protocol[6] at 25 μmol scale. Peptide resin containing a single primary amine on a specific Lys side chain was treated once with 2 mL 100 mM Fmoc-Ddap-OH in NMP or DMF overnight (15-20 h; most Ddap couplings are done in 3 h), with mixing on a rotisserie at 37 °C. After the coupling reaction was complete, the peptide resin was washed three times with DMF. Note that the unreacted Fmoc-Ddap-OH in NMP or DMF can be stored at -20 °C for recycling.

Manual Fmoc Deprotection

For 25 μmol scale syntheses, Fmoc was manually deprotected through two consecutive 3 min treatments of 2 mL 20% PIP in DMF, with mixing. After the second treatment, the resin was washed three times with DMF.

Manual Fmoc-Arg(Pbf)-OH Coupling

For 25 μmol scale syntheses, Fmoc-Arg(Pbf)-OH was manually coupled by adding 700 μL 200 mM Fmoc-Arg(Pbf)-OH in DMF, 700 μL 195 mM HATU in DMF, and 500 μL 600 mM NMM in DMF to the peptide resin. The coupling reaction was rotated for 25 min. The coupling was repeated a second time. The resin was then washed three times with DMF.

Manual 6-azidohexanoic Acid Coupling

Coupling of 6-azidohexanoic acid to 25 μmol scale peptide on resin was achieved through a single 1 h treatment of 700 μL 200 mM 6-azidohexanoic acid in DMF, 700 μL 195 mM HATU in DMF, and 500 μL 600 mM NMM in DMF, with mixing. Resin was washed six times with DMF, followed by three DCM washes.

Manual DBCO-C6-NHS Ester Coupling

Coupling of DBCO to 25 μmol scale peptide on resin was achieved through one 2 h treatment of 1 mL 45 mM DBCO-C6-NHS ester in DMF and 1 mL 300 mM DIPEA in DMF, with mixing of the peptide resin on rotisserie at 37 °C. Once the coupling reaction was complete, the resin was washed six times with DMF, followed by three DCM washes.

Manual 6-maleimidohexanoic acid Coupling

Coupling of 6-maleimidohexanoic acid to 25 μmol scale peptide on resin was achieved through a single 1 h treatment of 700 μL 200 mM 6-maleimidohexanoic acid in DMF, 700 μL 195 mM HATU in DMF, and 500 μL 600 mM NMM in DMF, with mixing. Resin was washed six times with DMF, followed by three DCM washes.

Manual Ac-Cys(Trt)-OH Coupling

For 25 μmol scale syntheses, Ac-Cys(Trt)-OH was manually coupled by adding 700 μL 200 mM Ac-Cys(Trt)-OH in DMF, 700 μL 195 mM HATU in DMF, and 500 μL 600 mM NMM in DMF to the peptide resin. The coupling reaction was rotated for 25 min. The coupling was repeated a second time. The resin was then washed three times with DMF, followed by three DCM washes.

Manual Peptide Synthesis of Peptide with One Functionalized Glu HH

In order to couple various modifications on Glu(AlHx) helping hand linker (e.g., DBCO, maleimide, thiol, or azide), manual couplings/deprotections were performed on peptides containing a single Glu(AlHx) residue after automated peptide synthesis and N-terminal capping was completed. Manual peptide syntheses were generally completed at either 25 or 50 μmol scale as previously reported.[7]

Manual Glu(AlHx-Dde) Deprotection

For 25 μmol scale Glu(AlHx-Dde) deprotection, peptide resin was treated with 300 μL allyl alcohol and 1.7 mL 5% hydrazine in DMF for three consecutive 5 min treatments, with mixing. Following the hydrazine treatment, the resin was washed six times with DMF. Follow "*Manual Peptide Synthesis of Peptide with One Functionalized Lys HH"* for functionalizing the Glu(AlHx) HH linker.

Manual Peptide Synthesis of Peptide with Two Functionalized Lys HHs

In order to couple various modifications on two specific Lys residues (e.g., Ddap linker, thiol, and azide), manual couplings/deprotections were performed on peptides containing a single Lys(Dde) and a single Lys(Mtt) residue after automated peptide synthesis and N-terminal capping was completed. For C-terminal peptide-$NHNH_2$, hydrazone resin was used (stable to Mtt removal).[3] See section on "*Preparation of Peptide Resins"* for more on hydrazone resin. Manual peptide syntheses were generally completed at either 25 or 50 μmol scale as previously reported.

Manual Lys(Dde) Deprotection, Coupling and Functionalization of the first HH with Azide

For 25 μmol scale, follow "*Manual Peptide Synthesis of Peptide with One Functionalized Lys HH"* to couple and functionalize the first HH linker with azide.

Manual Lys(Mtt) Deprotection

Following the first HH functionalization, the resin was washed six times with DMF, six times with DCM, and swelled in DCM for 30 min. For Lys(Mtt) deprotection, peptide resin was treated with 2 mL 2% TFA, 2% TIS for five consecutive 5 min treatments, with mixing, and single DCM wash between treatments. Following the TFA treatments, the resin was washed six times with DCM, three times with DMF, six times with 20% PIP in DMF (to quench residual TFA), six times DMF, and swelled in DMF for 15 min.

Coupling and Functionalization of the second HH with Cys

For 25 μmol scale, follow "*Manual Peptide Synthesis of Peptide with One Functionalized Lys HH"* to couple and functionalize the second HH linker with Cys.

Kaiser Test

To monitor the progress of manual deprotections and couplings, the Kaiser test[8] was used to observe the presence of primary amines on peptide resin. 6% ninhydrin in ethanol (either 190 or 200 proof) was used as the Kaiser reagent. To complete the Kaiser test, a few beads of peptide resin were aliquoted into a 1.5 mL Eppendorf tube. The resin aliquot was then mixed with 50 μL Kaiser reagent and vortexed for 3 s. The mixture was spun down for 3 s on a tabletop centrifuge and incubated at 90 °C for 5 min. If the solution and/or beads were blue/purple, then the resin was assumed to contain unreacted primary amines. If the Kaiser test gave an unexpected result, another round of deprotection or coupling was performed.

Peptide Resin Storage and Handling

Once all desired deprotections and couplings were completed, peptide resin was washed ≥ 3 times with DMF, followed by three washes with DCM. The resin was dried under vacuum for ≥ 30 min before being stored in a 4 °C desiccator. If additional deprotections and couplings needed to be performed on dried resin, it was equilibrated to r.t. and swelled in DMF for ≥ 30 min.

Dbz to Nbz Conversion

Following SPPS of peptide on Dawson resin, the Dbz to Nbz conversion was typically completed at 25 μmol scale based on the established protocol.[9] Peptide resin was washed three times with DCM, and swelled in DCM for 30 min. 30 mg of 4-nitrophenylchloroformate was dissolved in 4 mL of DCM and added to the resin while flowing Argon through the SPPS tube. The reaction was mixed for 45 min at r.t. The resin was then washed three times with DCM and treated twice with 5 mL of 0.5 M DIPEA in

DMF for 15 min at r.t. with mixing. The resin was washed six time with DMF then DCM, dried under vacuum for ≥ 30 min, and cleaved following *"Cleavage and Peptide Precipitation".*

Pen(Npys) to Stbu/SIT Conversion

Peptides containing Pen(Npys) were converted to Pen(Stbu) or Pen(SIT) as previously reported with minor alterations.[10] 25 μmol scale on-resin conversion was accomplished by treating the resin with 2 mL 200 mM SIT or t-BuSH in DMF with 200 mM TEA with mixing at 37 °C for 1 h. Following the conversion, the resin was washed six times with DMF. Conversion was performed prior to any additional on-resin modification such as HH functionalization.

Cleavage and Peptide Precipitation

25 μmol scale peptide cleavage was typically accomplished with a 3 h treatment of 3 mL TFA containing 2.5% water and 2.5% TIS, with mixing on a rotisserie at r.t. For peptides containing Cys(Trt)/Pen(Trt), 2.5% EDT was added to the TFA cocktail, unless DBCO, azide, or maleimide was also in the peptide. For peptides containing DBCO, the cleavage was conducted with Cu protection of the DBCO by adding 5 eq $(MeCN)_4CuBF_4$ (125 μmol, or 39.3 mg) to the dried peptide resin before addition of the standard TFA cocktail (95% TFA, 2.5% water, and 2.5% TIS) as previously reported.[5] For peptides containing both DBCO and Cys/Pen, the thiol was protected with an acid-stable group (Stbu or SIT) during cleavage with Cu protection. After cleavage, the TFA solution was added to ~30 mL ice-cold ethyl ether, shaken thoroughly, and placed at -20 °C for ≥ 30 min, in order to precipitate the crude peptide. The solution was then centrifuged at 4,700 *g*, 4 °C for ≥ 10 min. The resulting supernatant was decanted, and crude peptide pellets were washed twice with ~20 mL ice-cold ethyl ether. The crude peptide pellets were dried in a vacuum desiccator overnight.

Preparation of Crude Peptides for Analytical RP-HPLC, LC-MS, and Preparative RP-HPLC

25 μmol scale crude peptides were dissolved in 10-20% MeCN 0.1% TFA (~20-40 mL total volume) and were vortexed and sonicated to dissolve as much material as possible, followed by centrifugation at 4,700 *g*, 4 °C for 10 min prior to analytical RP-HPLC, preparative RP-HPLC, and/or LC-MS. Smaller and larger scale sample preparations were conducted by adjusting the amount of crude peptide and solvent adequately.

Analytical LC-MS Methods

0.1% formic acid in water (Buffer A) and 0.1% formic acid in MeCN (Buffer B) were used as mobile phases for LC-MS analyses. Mass spectra were obtained on an Agilent 6120 single-quadrupole mass spectrometer in fast scan/positive ion mode with an Agilent 1260 Infinity II front-end. UV data were collected using the Agilent 1260 Infinity II diode array detector (200-600 nm). Unless otherwise noted, observed masses were calculated using the charge states from averaged scans across the major ion signal and corresponding UV peak. Calculated and observed masses are presented as average mass. LC-MS methods are described below:

- **LC-MS Method A:** Agilent Poroshell 2.7 μm EC-C18 (120 Å, 4.6 x 50 mm); 50 °C; gradient: 0-1 min 5% B, 1-8 min 5-90% B, 8-8.1 min 90-5% B, 8.1-10 min 5% B; flow rate: 0-8 min 0.75 mL/min, 8-8.1 min 0.75-1.0 mL/min, 8.1-10 min 1.0 mL/min; scan range: 400-2,000 *m/z*; voltage: 50 V
- **LC-MS Method B:** Phenomenex Aeris Widepore 3.6 μm C4 (200 Å, 2.1 x 50 mm) 50 °C; gradient: 0-1 min 5% B, 1-8 min 5-90% B, 8-8.1 min 90-5% B, 8.1-10 min 5% B; flow rate: 0-10 min 0.5 mL/min; scan range: 100-2,000 *m/z*; voltage: 50 V

- **LC-MS Method C:** Agilent Poroshell 2.7 µm EC-C18 (120 Å, 4.6 x 50 mm); 50 °C; gradient: 0-1 min 3% B, 1-11 min 3-26% B, 11-11.1 min 26-3% B, 11.1-13 min 3% B; flow rate: 0-14 min 0.75 mL/min, 14-14.1 min 0.75-1.0 mL/min, 14.1-16 min 1.0 mL/min; scan range: 400-2,000 *m/z*; voltage: 50 V
- **LC-MS Method D:** Agilent Poroshell 2.7 µm EC-C18 (120 Å, 4.6 x 50 mm); 50 °C; gradient: 0-1 min 3% B, 1-8 min 3-88% B, 8-8.1 min 88-3% B, 8.1-10 min 3% B; flow rate: 0-8 min 0.75 mL/min, 8-8.1 min 0.75-1.0 mL/min, 8.1-10 min 1.0 mL/min; scan range: 400-2,000 *m/z*; voltage: 50 V
- **LC-MS Method E:** Phenomenex Aeris Widepore 3.6 µm C4 (200 Å, 2.1 x 50 mm) 50 °C; gradient: 0-4 min 5% B, 4-11 min 5-90% B, 11-11.1 min 90-5% B, 11.1-14 min 5% B; flow rate: 0-14 min 0.5 mL/min; scan range: 400-2,000 *m/z*; voltage: 50 V
- **LC-MS Method F:** Phenomenex Aeris Widepore 3.6 µm C4 (200 Å, 2.1 x 50 mm) 50 °C; gradient: 0-4 min 15% B, 4-11 min 15-90% B, 11-11.1 min 90-15% B, 11.1-14 min 15% B; flow rate: 0-14 min 0.5 mL/min; scan range: 400-2,000 *m/z*; voltage: 50 V
- **LC-MS Method G:** Phenomenex Aeris Widepore 3.6 µm C4 (200 Å, 2.1 x 50 mm) 50 °C; gradient: 0-1 min 5% B, 1-16 min 5-70% B, 16-16.1 min 70-5% B, 16.1-18 min 5% B; flow rate: 0-18 min 0.5 mL/min; scan range: 400-2,000 *m/z*; voltage: 50 V

Analytical RP-HPLC Methods

0.1% TFA in water (Buffer A) and 0.1% TFA in 90% MeCN (Buffer B) were used as mobile phases for analytical RP-HPLC analyses. Traces were collected on an Agilent 1260 Infinity II instrument at A_{214}. Analytical RP-HPLC methods are described below:

- **Analytical Method A:** Phenomenex Jupiter 4 µm C12 (90 Å, 4.6 x 150 mm); 40 °C; gradient: 0-2 min 10% B, 2-27 min 10-90% B, 27-30 min 90% B, 30-30.1 min 90-10% B, 30.1-34 min 10% B; flow rate: 1.0 mL/min
- **Analytical Method B:** Phenomenex Jupiter 4 µm C12 (90 Å, 4.6 x 150 mm); 40 °C; gradient: 0-1 min 20% B, 1-26 min 20-50% B, 26-26.1 min 50-90% B, 26.1-27.1 min 90% B, 27.1-27.2 min 90-20% B, 27.2-29 min 20% B; flow rate: 1.0 mL/min
- **Analytical Method C:** Phenomenex bioZen 3.6 µm Intact C4 (200 Å, 4.6 x 150 mm); 45 °C; gradient: 0-5 min 10% B, 5-20 min 10-50% B, 20-20.1 min 50-90% B, 20.1-22 min 90% B, 22-22.1 min 90-10% B, 22.1-25 min 10% B; flow rate: 2.0 mL/min
- **Analytical Method D:** Phenomenex Jupiter 5 µm C4 (300 Å, 4.6 x 150 mm); 40 °C; gradient: 0-5 min 10% B, 5-35 min 10-50% B, 35-35.1 min 50-90% B, 35.1-36 min 90% B, 36-36.1 min 90-10% B, 36.1-38 min 10% B; flow rate: 1.0 mL/min
- **Analytical Method E:** Phenomenex Jupiter 4 µm C12 (90 Å, 4.6 x 150 mm); 50 °C; gradient: 0-5 min 5% B, 5-30 min 5-35% B, 30-30.1 min 35-90% B, 30.1-31.1 min 90% B, 31.1-31.2 min 90-5% B, 31.2-33 min 5% B; flow rate: 1.0 mL/min
- **Analytical Method F:** Phenomenex Jupiter 4 µm C12 (90 Å, 4.6 x 150 mm); 50 °C; gradient: 0-1 min 10% B, 1-16 min 10-40% B, 16-16.1 min 40-90% B, 16.1-17.1 min 90% B, 17.1-17.2 min 90-10% B, 17.2-19 min 10% B; flow rate: 1.0 mL/min
- **Analytical Method G:** Phenomenex Jupiter 4 µm C12 (90 Å, 4.6 x 150 mm); 50 °C; gradient: 0-1 min 5% B, 1-16 min 5-35% B, 16-16.1 min 35-90% B, 16.1-17.1 min 90% B, 17.1-17.2 min 90-5% B, 17.2-19 min 5% B; flow rate: 1.0 mL/min

- **Analytical Method H:** Phenomenex Jupiter 5 µm C4 (300 Å, 4.6 x 150 mm); 40 °C; gradient: 0-5 min 1% B, 5-30 min 1-31% B, 30-30.1 min 31-90% B, 30.1-31.1 min 90% B, 31.1-31.2 min 90-1% B, 31.2-33 min 1% B; flow rate: 1.0 mL/min
- **Analytical Method I:** Phenomenex Jupiter 5 µm C4 (300 Å, 4.6 x 150 mm); 40 °C; gradient: 0-10 min 5% B, 10-35 min 5-45% B, 35-35.1 min 45-90% B, 35.1-36.1 min 90% B, 36.1-36.2 min 90-5% B, 36.2-38 min 5% B; flow rate: 1.0 mL/min
- **Analytical Method J:** Phenomenex Jupiter 5 µm C4 (300 Å, 4.6 x 150 mm); 50 °C; gradient: 0-3 min 20% B, 3-28 min 20-50% B, 28-28.1 min 50-90% B, 28.1-29.1 min 90% B, 29.1-29.2 min 90-20% B, 29.2-31 min 20% B; flow rate: 1.0 mL/min
- **Analytical Method K:** Phenomenex Jupiter 5 µm C4 (300 Å, 4.6 x 150 mm); 50 °C; gradient: 0-8 min 20% B, 8-33 min 20-50% B, 33-33.1 min 50-90% B, 33.1-34.1 min 90% B, 34.1-34.2 min 90-20% B, 34.2-36 min 20% B; flow rate: 1.0 mL/min
- **Analytical Method L:** Phenomenex Kinetex 5 µm C18 (100 Å, 4.6 x 150 mm); 40 °C; gradient: 0-2 min 5% B, 2-32 min 5-100% B, 32-35 min 100% B, 35-35.1 min 100-5% B, 35.1-36 min 5% B, flow rate: 4.0 mL/min
- **Analytical Method M:** Phenomenex bioZen 3.6 µm Intact C4 (200 Å, 4.6 x 150 mm); 45 °C; gradient: 0-5 min 20% B, 5-35 min 20-70% B, 35-35.1 min 70-90% B, 35.1-36 min 90% B, 36-36.1 min 90-10% B, 36.1-38 min 10% B; flow rate: 2.0 mL/min

Preparative RP-HPLC Methods

0.1% TFA in water (Buffer A) and 0.1% TFA in 90% MeCN (Buffer B) were used as mobile phases for preparative RP-HPLC purifications. Peptide purifications were performed on an Agilent 1260 Infinity LC system semi-preparative or Agilent 1260 Infinity II preparative system. RP-HPLC purification methods are described below:

- **Purification Method A:** Phenomenex Kinetex 5 µm C18 (100 Å, 10 x 250 mm); 45 °C; gradient: 0-10 min 10% B, 10-11 min 10-20% B, 11-36 min 20-35% B, 36-37 min 40-90% B, 37-42 min 90% B, 42-43 min 90-10% B, 43-46 min 10% B; flow rate: 4.0 mL/min
- **Purification Method B:** Phenomenex Kinetex 5 µm C18 (100 Å, 10 x 250 mm); 45 °C; gradient: 0-10 min 10% B, 10-11 min 10-35% B, 11-36 min 35-50% B, 36-37 min 50-90% B, 37-42 min 90% B, 42-43 min 90-10% B, 43-46 min 10% B; flow rate: 4.0 mL/min
- **Purification Method C:** Phenomenex Kinetex 5 µm C18 (100 Å, 10 x 250 mm); 45 °C; gradient: 0-4 min 10% B, 4-5 min 10-30% B, 5-30 min 30-38% B, 30-31 min 38-90% B, 31-36 min 90% B, 36-37min 90-10% B, 37-40 min 10% B; flow rate: 4.0 mL/min
- **Purification Method D:** Phenomenex Jupiter 4 µm C12 (90 Å, 21.20 x 250 mm); gradient: 0-4 min 20% B, 4-5 min 20-34% B, 5-30 min 34-40% B, 30-30.1 min 40-90% B, 30.1-34 min 90% B, 34-34.1 min 90-20% B, 34.1-38.5 min 20% B; flow rate: 15.0 mL/min
- **Purification Method E:** Phenomenex Jupiter 4 µm C12 (90 Å, 21.20 x 250 mm); gradient: 0-4 min 10% B, 4-5 min 10-40% B, 5-30 min 40-46% B, 30-30.1 min 46-90% B, 30.1-34 min 90% B, 34-34.1 min 90-10% B, 34.1-38.5 min 10% B; flow rate: 15.0 mL/min

- **Purification Method F:** Phenomenex Kinetex 5 µm C18 (100 Å, 10 x 250 mm); 45 °C; gradient: 0-4 min 10% B, 4-5 min 10-32% B, 5-30 min 32-40% B, 30-31 min 40-90% B, 31-36 min 90% B, 36-37min 90-10% B, 37-40 min 10% B; flow rate: 4.0 mL/min
- **Purification Method G:** Phenomenex Jupiter 4 µm C12 (90 Å, 21.20 x 250 mm); gradient: 0-4 min 10% B, 4-5 min 10-26% B, 5-30 min 26-32% B, 30-30.1 min 32-90% B, 30.1-34 min 90% B, 34-34.1 min 90-10% B, 34.1-38.5 min 10% B; flow rate: 15.0 mL/min
- **Purification Method H:** Phenomenex Kinetex 5 µm C18 (100 Å, 10 x 250 mm); 45 °C; gradient: 0-4 min 10% B, 4-5 min 10-39% B, 5-30 min 39-47% B, 30-31 min 47-90% B, 31-36 min 90% B, 36-37min 90-10% B, 37-40 min 10% B; flow rate: 4.0 mL/min
- **Purification Method I:** Phenomenex Kinetex 5 µm C18 (100 Å, 10 x 250 mm); 45 °C; gradient: 0-5 min 10% B, 5-30 min 10-18% B, 30-31 min 18-90% B, 31-36 min 90% B, 36-37min 90-10% B, 37-40 min 10% B; flow rate: 4.0 mL/min
- **Purification Method J:** Phenomenex Kinetex 5 µm C18 (100 Å, 10 x 250 mm); 45 °C; gradient: 0-4 min 10% B, 4-5 min 10-13% B, 5-30 min 13-21% B, 30-31 min 21-90% B, 31-36 min 90% B, 36-37min 90-10% B, 37-40 min 10% B; flow rate: 4.0 mL/min
- **Purification Method K:** Phenomenex Kinetex 5 µm C18 (100 Å, 10 x 250 mm); 45 °C; gradient: 0-4 min 10% B, 4-5 min 10-15% B, 5-30 min 15-23% B, 30-31 min 23-90% B, 31-36 min 90% B, 36-37min 90-10% B, 37-40 min 10% B; flow rate: 4.0 mL/min
- **Purification Method L:** Phenomenex Jupiter 4 µm C12 (90 Å, 21.20 x 250 mm); gradient: 0-4 min 10% B, 4-5 min 10-23% B, 5-30 min 23-29% B, 30-30.1 min 29-90% B, 30.1-34 min 90% B, 34-34.1 min 90-10% B, 34.1-38.5 min 10% B; flow rate: 15.0 mL/min
- **Purification Method M:** Phenomenex Kinetex 5 µm C18 (100 Å, 10 x 250 mm); 45 °C; gradient: 0-5 min 5% B, 5-30 min 5-25% B, 30-31 min 25-90% B, 31-36 min 90% B, 36-37min 90-5% B, 37-40 min 5% B; flow rate: 4.0 mL/min
- **Purification Method N:** Phenomenex Jupiter 4 µm C12 (90 Å, 21.20 x 250 mm); gradient: 0-4 min 10% B, 4-5 min 10-17% B, 5-30 min 17-23% B, 30-30.1 min 23-90% B, 30.1-34 min 90% B, 34-34.1 min 90-10% B, 34.1-38.5 min 10% B; flow rate: 15.0 mL/min
- **Purification Method O:** Phenomenex Kinetex 5 µm C18 (100 Å, 10 x 250 mm); 45 °C; gradient: 0-4 min 10% B, 4-5 min 10-15% B, 5-30 min 15-25% B, 30-31 min 25-90% B, 31-36 min 90% B, 36-37min 90-10% B, 37-40 min 10% B; flow rate: 4.0 mL/min
- **Purification Method P:** Phenomenex Jupiter 4 µm C12 (90 Å, 21.20 x 250 mm); gradient: 0-4 min 10% B, 4-5 min 10-20% B, 5-30 min 20-26% B, 30-30.1 min 26-90% B, 30.1-34 min 90% B, 34-34.1 min 90-10% B, 34.1-38.5 min 10% B; flow rate: 15.0 mL/min
- **Purification Method Q:** Phenomenex Jupiter 4 µm C12 (90 Å, 21.20 x 250 mm); gradient: 0-4 min 10% B, 4-5 min 10-31% B, 5-30 min 31-37% B, 30-30.1 min 37-90% B, 30.1-34 min 90% B, 34-34.1 min 90-10% B, 34.1-38.5 min 10% B; flow rate: 15.0 mL/min

- **Purification Method R:** Phenomenex Jupiter 4 µm C12 (90 Å, 21.20 x 250 mm); gradient: 0-4 min 10% B, 4-5 min 10-32% B, 5-30 min 32-38% B, 30-30.1 min 38-90% B, 30.1-34 min 90% B, 34-34.1 min 90-10% B, 34.1-38.5 min 10% B; flow rate: 15.0 mL/min
- **Purification Method S:** Phenomenex Jupiter 4 µm C12 (90 Å, 21.20 x 250 mm); gradient: 0-4 min 10% B, 4-5 min 10-35% B, 5-30 min 35-41% B, 30-30.1 min 41-90% B, 30.1-34 min 90% B, 34-34.1 min 90-10% B, 34.1-38.5 min 10% B; flow rate: 15.0 mL/min
- **Purification Method T:** Phenomenex Jupiter 4 µm C12 (90 Å, 21.20 x 250 mm); gradient: 0-4 min 10% B, 4-5 min 10-25% B, 5-30 min 25-31% B, 30-30.1 min 31-90% B, 30.1-34 min 90% B, 34-34.1 min 90-10% B, 34.1-38.5 min 10% B; flow rate: 15.0 mL/min
- **Purification Method U:** Phenomenex Jupiter 4 µm C12 (90 Å, 21.20 x 250 mm); gradient: 0-4 min 10% B, 4-5 min 10-29% B, 5-30 min 29-35% B, 30-30.1 min 35-90% B, 30.1-34 min 90% B, 34-34.1 min 90-10% B, 34.1-38.5 min 10% B; flow rate: 15.0 mL/min
- **Purification Method V:** Phenomenex Jupiter 5 µm C4 (300 Å, 10 x 250 mm); 50 °C; gradient: 0-10 min 20% B, 10-10.5 min 20-38% B, 10.5-35.5 min 38-46% B, 35.5-36 min 46-90% B, 36-40 min 90% B, 40-40.1 min 90-20% B, 40.1-45 min 20% B; flow rate: 4.0 mL/min
- **Purification Method W:** Phenomenex Jupiter 5 µm C4 (300 Å, 10 x 250 mm); 50 °C; gradient: 0-10 min 20% B, 10-10.5 min 20-30% B, 10.5-59.5 min 30-46% B, 59.5-60 min 46-90% B, 60-64 min 90% B, 64-64.1 min 90-20% B, 64.1-69 min 20% B; flow rate: 4.0 mL/min
- **Purification Method X:** Waters XBridge Peptide BEH300 10 µm C18 (300 Å, 19 x 250 mm); gradient: 0-4 min 10% B, 4-5 min 10-25% B, 5-30 min 25-39% B, 30-30.1 min 39-90% B, 30.1-34 min 90% B, 34-34.1 min 90-10% B, 34.1-38.5 min 10% B; flow rate: 15.0 mL/min
- **Purification Method Y:** Waters XBridge Peptide BEH300 10 µm C18 (300 Å, 19 x 250 mm); gradient: 0-4 min 10% B, 4-5 min 10-35% B, 5-30 min 35-45% B, 30-30.1 min 45-90% B, 30.1-34 min 90% B, 34-34.1 min 90-10% B, 34.1-38.5 min 10% B; flow rate: 15.0 mL/min
- **Purification Method Z:** Waters XBridge Peptide BEH300 10 µm C18 (300 Å, 19 x 250 mm); gradient: 0-4 min 10% B, 4-5 min 10-30% B, 5-30 min 30-45% B, 30-30.1 min 45-90% B, 30.1-34 min 90% B, 34-34.1 min 90-10% B, 34.1-38.5 min 10% B; flow rate: 15.0 mL/min
- **Purification Method AA:** Waters XBridge Peptide BEH300 10 µm C18 (300 Å, 19 x 250 mm); gradient: 0-4 min 10% B, 4-5 min 10-35% B, 5-30 min 35-60% B, 30-30.1 min 60-90% B, 30.1-34 min 90% B, 34-34.1 min 90-10% B, 34.1-38.5 min 10% B; flow rate: 15.0 mL/min

Fractions collected during preparative RP-HPLC purifications were analyzed by LC-MS to assess purity. All pure fractions were pooled and lyophilized to obtain dry, pure peptide which was then analyzed by analytical RP-HPLC and LC-MS.

Note that during purification of DBCO peptides and peptide-Nbz, the crude peptide solution and collected fractions were stored on ice to reduce DBCO degradation and/or Nbz hydrolysis.

Preparation of Analytical RP-HPLC and LC-MS Traces

Analytical RP-HPLC and LC-MS traces were prepared for publication using our in-house Automated Trace Maker (ATM) programs. Using .CSV files of analytical RP-HPLC or LC-MS data, these Python scripts generate the desired chromatograms that can be viewed in Microsoft Excel. The ATM programs are available for free use on the Kay Lab Github website: https://github.com/kay-lab.

Calculation of Maleimide A_{280} Extinction Coefficient

10 mg of 6-maleimidohexanoic acid was dissolved in 500 µL ligation buffer (6 M GnHCl, 100 mM phosphate, pH 7). This 95 mM 6-maleimidohexanoic acid solution was used in a 2-fold dilution series. For each dilution, triplicate absorbance measurements were taken at 280 nm using a NanoDrop One^{C} instrument (Thermo Scientific) blanked with ligation buffer. The average A_{280} values were then used to calculate the extinction coefficient using Beer's Law.

The maleimide extinction coefficient was estimated as:

- 280 nm – ~365 $M^{-1}cm^{-1}$

Calculation of Nbz A_{280} Extinction Coefficient

Peptide-Nbz was dissolved in 500 µL ligation buffer (6 M GnHCl, 100 mM phosphate, pH 7). The peptide-Nbz was treated with 1 M NH_2OH to force Nbz hydrolysis. A 2-fold dilution series was conducted on the analytical RP-HPLC at 280 nm to compare absorbance (area under the peak) to a non-treated solution of peptide-Nbz to calculate the absorbance contribution of the Nbz group.

The Nbz extinction coefficient was estimated as:

- 280 nm – ~10,931 $M^{-1}cm^{-1}$

Calculation of Peptide Concentrations

For peptides containing Trp, Tyr, Stbu/SIT-protected Cys/Pen, Ddap, maleimide, Nbz and/or DBCO, in-solution concentrations were determined using A_{280} measurements collected on a NanoDrop One^{C} instrument. The following extinction coefficients were used to calculate concentrations via Beer's Law:

- Trp – 5,500 $M^{-1}cm^{-1}$
- Tyr – 1,490 $M^{-1}cm^{-1}$
- Stbu/SIT-Cys/Pen – 125 $M^{-1}cm^{-1}$
- Ddap – 14,600 $M^{-1}cm^{-1}$
- DBCO – 14,000 $M^{-1}cm^{-1}$[5]
- Maleimide – 365 $M^{-1}cm^{-1}$
- Nbz – 10,931 $M^{-1}cm^{-1}$

Intermolecular Native Chemical Ligation (NCL) using Peptide-$NHNH_2$

To perform intermolecular NCL of two peptide segments by peptide-$NHNH_2$ activation, the peptide-$NHNH_2$ was dissolved (~1-1.5 mM) and activated (conversion of hydrazide to acyl azide to thioester) in activation buffer (6 M GnHCl, 100 mM phosphate, pH 3) for 20 min at -20 ºC by addition of freshly prepared 15 eq sodium nitrite ($NaNO_2$). Following activation, a solution containing freshly prepared MPAA pH 7 in ligation buffer (6 M GnHCl, 100 mM phosphate, pH 7) was added to a concentration of 100 mM, and the final pH was adjusted to 6.8 to initiate thiolysis. Thiol-containing peptide was dissolved in ligation buffer to ~1.5-2 mM and added to the ligation reaction (1.2 eq to peptide thioester). After 10 min, 20 mM TCEP in ligation buffer was added to the reaction and the reaction was left to rotate on a rotisserie at r.t until complete. Time points for the intermolecular NCL reactions were taken by diluting an aliquot of

reaction 1:10 with 200 mM TCEP in LC-MS-grade water (pH adjusted to 7.0 prior to addition). Acetic acid was added to a final concentration of ~5% and the dilution was centrifuged at 18,000 *g*, r.t for 10 min prior to analytical RP-HPLC and/or LC-MS analyses. Upon completion (based on analytical RP-HPLC and LC-MS), reactions were treated with 1:10 with 200 mM TCEP in LC-MS-grade water (pH adjusted to 7.0 prior to addition). Acetic acid was added to a final concentration of ~5%, spun at 5000 g, and the supernatant was purified by preparative RP-HPLC or stored at -80 °C.

Intermolecular Native Chemical Ligation (NCL) using peptide-Nbz

To perform intermolecular NCL of two peptide segments by peptide-Nbz activation, the peptides were dissolved in ligation buffer and combined to ~1-1.5 mM. Freshly prepared MPAA pH 7 in ligation buffer was added to a concentration of 100 mM, and the final pH was adjusted to 6.8 to initiate the reaction. After 10 min, 20 mM TCEP in ligation buffer was added to the reaction and the reaction was left to rotate on a rotisserie at r.t until complete. Time points were taken to monitor the reaction (follow *Intermolecular Native Chemical Ligation (NCL) using Peptide-NHNH$_2$*).

SPAAC of DBCO Peptides with Azide Peptides

SPAAC of DBCO and azide peptides was achieved as previously reported.[5] Briefly, purified DBCO and azide peptides were separately dissolved in activation buffer to ~1.5-2 mM. The DBCO and azide peptide solutions were mixed, and activation buffer was added to dilute the solution to contain an approximately equimolar concentration of both peptides (~1.0 mM peptide). The SPAAC reaction was placed on a rotisserie at r.t. for 2 h. Time points were taken by diluting a reaction aliquot 1:20 with LC-MS-grade water, and 3 mM 6-azidoheexanoic acid (prepared in 20% RP-HPLC buffer B) to quench the reaction, followed by vortexing and centrifuging the dilution at 18,000 *g*, r.t. for 10 min prior to analytical RP-HPLC and/or LC-MS. Completed reactions were directly taken into the next reaction or stored at -80 °C.

Conjugation of Maleimide Peptides with Thiol Peptides

Purified maleimide and thiol (Cys) peptides were separately dissolved in activation buffer to ~1-2 mM. The maleimide and thiol peptide solutions were mixed, and activation buffer was added to dilute the solution to contain an approximately equimolar concentration of both peptides (~1.0 mM peptide). The conjugation reaction was placed on a rotisserie at r.t. for 10 min (reaction is done at 10 min although, occasionally, the reaction was left for 2 h). Time points were taken by diluting a reaction aliquot 1:20 with 20%B RP-HPLC buffer, followed by vortexing and centrifuging the dilution at 18,000 *g*, r.t. for 10 min prior to analytical RP-HPLC and/or LC-MS. Completed reactions were directly taken into the next reaction or stored at -80 °C.

Click-Assisted NCL (CAN) of Two Peptide Segments by peptide-NHNH$_2$ Activation

To perform CAN of two peptide segments (where peptide 1 is a peptide-NHNH$_2$), the conjugate (either linked via maleimide/thiol conjugation or SPAAC) was treated as previously described.[5] In brief, the peptide-NHNH$_2$ was converted into acyl azide using 15 eq $NaNO_2$ at pH 3 and *in situ* MPAA thioester formation occurred at pH 6.8 with 100 mM MPAA. Stbu was reduced with 100 mM TCEP after the first 10 min. The NCL reaction was placed on a rotisserie at r.t. until complete. Time points were taken to monitor the reaction (follow *Intermolecular Native Chemical Ligation (NCL) using peptide-NHNH$_2$*).

Click-Assisted NCL (CAN) of Two Peptide Segments by peptide-Nbz Activation

To perform CAN of two peptide segments (where peptide 1 is a peptide-Nbz), the conjugate (either linked via maleimide/thiol conjugation or SPAAC) was treated with 100 mM MPAA to convert the peptide-Nbz to the thioester at pH 6.8. After 10 min, Stbu was

reduced with 100 mM TCEP. The NCL reaction was placed on a rotisserie at r.t. until complete. Time points were taken to monitor the reaction (follow *Intermolecular Native Chemical Ligation (NCL) using Peptide-NHNH$_2$*).

Conjugation and SPAAC of Three Peptides

To conjugate three peptides together, maleimide/thiol conjugation was first performed as described above (follow *Conjugation of Maleimide Peptides with Thiol Peptides*). After maleimide/thiol conjugation, SPAAC was performed as described above (follow *SPAAC of DBCO Peptides with Azide Peptides*), to click the third peptide. The peptide concentrations were adjusted accordingly from the first conjugation reaction to achieve ~1:1 ratios when performing SPAAC (whether after the first NCL or the conjugation). Completed reactions were directly taken into the next reaction or stored at -80 °C.

Click-Assisted NCL (CAN) of Three Peptide Segments by Peptide-NHNH$_2$ Activation

To perform CAN of three peptide segments (where peptide 1 and peptide 2 are peptide-NHNH$_2$), the conjugate was treated as previously reported with minor adjustments.[5] Peptide-NHNH$_2$ were converted into acyl azide using 20 eq $NaNO_2$ at pH 3 and *in situ* MPAA thioester formation occurred at pH 6.8 with 100 mM MPAA. Stbu was reduced with 100 mM TCEP after the first 10 min. The NCL reaction was placed on a rotisserie at r.t. until complete. Time points were taken to monitor the reaction (follow *Intermolecular Native Chemical Ligation (NCL) using Peptide-NHNH$_2$*). Completed reactions were directly taken into the next reaction or stored at -80 °C.

Controlled Activation of Peptides for Templated NCL (CAPTN) of Three Peptide Segments

Directly after maleimide/thiol conjugation of the first two peptides (follow *Conjugation and SPAAC of Three Peptides*), peptide-Nbz NCL was initiated by the addition of MPAA to 100 mM and adjusting the pH to 6.8 (follow *Click-Assisted NCL (CAN) of Two Peptide Segments by peptide-Nbz Activation* but without TCEP to avoid azide reduction). The peptide-Nbz NCL reaction was placed on a rotisserie at r.t. until completion (unless otherwise specified). Time points were taken to monitor the reaction (follow *Intermolecular Native Chemical Ligation (NCL) using Peptide-NHNH$_2$*). Following peptide-Nbz NCL, MPAA extraction was done (follow *MPAA Extraction with Et_2O*). The third peptide was then conjugated via SPAAC as described above (follow *Conjugation and SPAAC of Three Peptides*). The peptide concentrations were adjusted accordingly from the first conjugation reaction to achieve ~1:1 ratio when performing SPAAC. Peptide-NHNH$_2$ NCL was initiated using 15 eq $NaNO_2$ at pH 3 and *in situ* MPAA thioester formation occurred at pH 6.8 with 100 mM MPAA. Stbu was reduced with 100 mM TCEP after the first 10 min. The NCL reaction was placed on a rotisserie at r.t. until completion. Time points were taken to monitor the reaction (follow *Intermolecular Native Chemical Ligation (NCL) using Peptide-NHNH$_2$*).

MPAA Extraction with Et_2O

MPAA extraction was achieved by first lowering the pH to 3 by adding HCl. Et_2O (1.5x v/v in relationship to the aqueous layer) was added, vortexed, and spun down. The Et_2O was removed and the extraction was repeated for a total of six times.

Desulfurization

"Desulfurization buffer" (6 M GnHCl, 100 mM $NaPO_4$, pH 6.5) was first sparged with argon gas for ≥ 20 min. 400 mM reduced glutathione, 200 mM VA-044 in desulfurization buffer (500 µL) was prepared, along with a solution of 600 mM TCEP in desulfurization buffer (500 µL). The VA-044/GSH solution was added to a final concentration of was 60 mM VA-044 and 120 mM GSH, followed by TCEP to a final concentration of 150 mM (unless otherwise specified). The resulting desulfurization reaction was briefly vortexed and carefully pH adjusted to 6.5 with NaOH. The desulfurization reaction was then covered with argon gas and placed on a rotisserie at

37 °C until completion (unless otherwise specified). Time points were taken by first diluting 60 µL desulfurization reaction in 60 µL 200 mM TCEP in LC-MS-grade water (pH adjusted to 7), vortexing, and then 6.6 µL acetic acid was added. After vortexing, the dilution was centrifuged at 18,000 *g*, r.t. for 10 min prior to analytical RP-HPLC and LC-MS analyses. The finished desulfurization reaction was stored at -80 °C until the next reactions or purified by RP-HPLC.

Ddap Removal

Ddap removal buffer (~2 M hydroxylamine in desulfurization buffer, pH 6.8-7.0) was prepared by dissolving hydroxylamine in the full volume of desulfurization buffer needed (with vortexing and sonication), and pH adjusted to 6.8-7.0 with NaOH. The Ddap cleavage reaction was performed as previously reported.[1] The cleavage was initiated by diluting the peptide solution 1:1 with Ddap cleavage buffer. The reaction was then vortexed, and the pH was verified to be ~6.8. The cleavage reaction was placed on a r.t. rotisserie for 2 h (unless otherwise specified). Time points of the Ddap cleavage reaction were taken by diluting a reaction aliquot 1:1 with 20% acetic acid in LC-MS-grade water. After vortexing the dilution, centrifugation was performed at 18,000 *g*, r.t. for 10 min prior to analytical RP-HPLC and LC-MS analyses. Once Ddap cleavage was complete, the reaction was taken into the next reaction or stored at -80 °C.

AlHx Removal with Pd/TPPTS

The AlHx removal was performed as previously reported.[1] Briefly, 350 mM GSH solution was prepared in degassed ligation buffer and pH adjusted to 8.0, 188 mM $Pd(OAc)_2$ was prepared in degassed DMF, and 850 mM TPPTS was prepared in degassed ddH_2O. The peptide was dissolved to ~0.5 mM in degassed ligation buffer. The $Pd(OAc)_2$ and TPPTS solutions were mixed in a 1:1 ratio, then added to the peptide to a final concentration of 20 mM $Pd(OAc)_2$. The reaction was briefly mixed with vortexing prior to adding GSH to a final concentration of 10 mM. The pH of the reaction was adjusted to 8.0, the reaction was degassed, and placed on the rotisserie at 37 °C until complete. Time points of the reaction were taken by treating with a 1:1 ratio of 500 mM DTT (prepared in ddH_20, pH 7.0) for 10 min at r.t. to quench the Pd. The time points were then acidified by adding acetic acid to 5% followed by vortexing and centrifugation at 18,000 *g*, r.t. for 10 min prior to analytical RP-HPLC and LC-MS analyses. Once AlHx removal was complete, the reaction was taken into the next reaction or stored at -80 °C.

AlHx Removal with $[Pd(allyl)Cl]_2$

The AlHx removal was performed as previously reported.[1] Briefly, the $[Pd(allyl)Cl]_2$ (25 eq) and GSH (25 eq) solution was prepared in sparged ligation buffer and added to the reaction containing the AlHx-modified conjugate. The reaction was pH adjusted to 8.0, degassed, and placed a 37 °C with mixing until completion. Time points of the reaction were taken by treating with a 40 mM DTT (prepared in ddH_20, pH 7.0) for 10 min at r.t. to quench the Pd. The time points were then acidified by adding acetic acid to 5% followed by vortexing and centrifugation at 18,000 *g*, r.t. for 10 min prior to analytical RP-HPLC and LC-MS analyses. Once AlHx removal was completed, the reaction was stored at -80 °C.

Acm Removal

The Acm removal was performed as previously reported.[11] Briefly, 200 mM $PdCl_2$ solution was prepared in degassed ligation buffer and added to the peptide solution to a final concentration of 5 mM $PdCl_2$. The reaction was briefly mixed with vortexing prior to adjusting the pH of the reaction to 7.0. The reaction was degassed, and placed on the rotisserie at 37 °C until complete. Time points of the reaction were taken by treating with a 1:1 ratio of 500 mM DTT (prepared in ddH_20, pH 7.0) for 10 min at r.t. to quench the Pd. The time points were then acidified by adding acetic acid to 5%. After vortexing, centrifugation was performed at 18,000 *g*, r.t. for 10

min prior to analytical RP-HPLC and LC-MS analyses. Once Acm removal was complete, the reaction was purified by semi-preparative RP-HPLC.

SECTION 3: Supplementary Table

Table S1. Summary of all peptides synthesized in this work

Peptide	Sequence	Calculated MW (Da)	Comments
1a	Ac-RRRYSTEVEK(Ddap-R-C)NV-NHNH$_2$	2143.3	Maleimide/thiol-mediated CAN (Figure S1A)
2a	H$_2$N-C(Stbu)GK(Ddap-R-Mal)ENTWY-NH$_2$	1685.8	Maleimide/thiol-mediated CAN (Figure S1B)
1	H$_2$N-ARRRYSTEVEK(Ddap-R-Mal)NV-NHNH$_2$	2220.5	Three-segment templated NCL with peptide-NHNH$_2$ (Figure S6A)
2	H$_2$N-C(Stbu)A*R*K(Ddap-R-C)EGSDFHI*R*PNMQWYLS*R*K(Ddap-R-N$_3$)TAV-NHNH$_2$	4174.0	Three-segment templated NCL with peptide-NHNH$_2$ and CAPTN (Figure S6B)
3	H$_2$N-C(Stbu)GK(Ddap-R-DBCO)ENTVY-NH$_2$	1721.1	Three-segment templated NCL with peptide-NHNH$_2$ and CAPTN (Figure S6C)
1b	H$_2$N-ARRRYSTEVEK(Ddap-R-DBCO)NVS-Nbz	2574.9	CAN with peptide-Nbz (Figure S10A)
1c	H$_2$N-ARRRYSTEVEK(Ddap-R-DBCO)NT-Nbz	2489.7	CAN with peptide-Nbz (Figure S10B)
2b	H$_2$N-C(Stbu)GK(Ddap-R-N$_3$)ENTVY-NH$_2$	1544.9	CAN with peptide-Nbz (Figure S10C)
1d	H$_2$N-ARRRYSTEVEKNVS-Nbz	1854.0	Intermolecular control for CAN with peptide-Nbz (Figure S11A)
1e	H$_2$N-ARRRYSTEVEKNT-Nbz	1768.8	Intermolecular control for CAN with peptide-Nbz (Figure S11B)
2c	H$_2$N-C(Stbu)GKENTVY-NH$_2$	1000.2	Intermolecular control for CAN with peptide-Nbz and MPAA-mediated Stbu removal (Figure S11C)
4b	H$_2$N-AGK(R-N$_3$)ENTVY-NH$_2$	1175.3	Azide stability assessment (Figure S16)
5b	Ac-RRRYSTEVEKNVG-NHNH$_2$	1649.7	Peptide-NHNH$_2$ activation studies (Figure S19A)
6b	H$_2$N-A*R*KDEAFGSAHILMNPQ*R*VTWY*R*KGS-NHNH$_2$	3015.4	Peptide-NHNH$_2$ activation studies (Figure S19B)
1'	H$_2$N-ARRRYSTEVEK(Ddap-R-Mal)NV-Nbz	2365.7	Three-segment CAPTN (Figure S24)
9	H$_2$N-MVTI*R*LA*R*HGAKK*R*PFYQ*VVV*ADS*R*NA*R*NGRFIERVGFFNPI-NHNH$_2$	4884.7	Intermolecular NCL of S16 (N'_S16, Figure S31A)
10	H$_2$N-CSEKEEGTRLDLDRIAHWVGQGATISDRVAALIKEVNKAA-OH	4351.9	Intermolecular NCL of S16 (C'_S16, Figure S31B)
12	H$_2$N-MVTI*R*LA*R*HGAKK*R*PFYQ*VVV*ADS*R*NA*R*NGRFIE(AlHx-N$_3$)RVGFFNPI-NHNH$_2$	5207.1	AlHx-mediated CAN of S16 (N_S16, Figure S33A)
13	H$_2$N-C(Stbu)SE(AlHx-DBCO)KEEGTRLDLDRIAHWVGQGATISDRVAALIKEVNKAA-OH	4938.7	AlHx-mediated CAN of S16 (C_S16, Figure S33B)
7a	Ac-AGENTWK(Ddap-R-DBCO)YG-NHNH$_2$	1802.1	CAN at Pen (Figure S37A)
8a	H$_2$N-PenGK(Ddap-R-N$_3$)ENTWY-NH$_2$	1571.8	CAN at Pen (Figure S37B)
7b	Ac-AGENTWKYG-NHNH$_2$	1081.2	Intermolecular control for CAN at Pen (Figure S38A)
8b	H$_2$N-PenGKENTWY-NH$_2$	1027.1	Intermolecular control for CAN at Pen and Pen(Stbu/SIT) reference (Figure S38B)
S17_T2-L84	H$_2$N-TDK*I*RTLQG*R*VVSDKMEKS*I*VVA*I*E*R*FVKHP*I*YGKF*I*K*R*TTKLHVHDENNECG*I*GDVVE *I*RECRPLSKTKSWTLV*R*VVEKAVL-OH	9555.2	SPPS attempt of full-length S17 (Figure S42B)

S17_T2-H45	H_2N-TDK*I*RTLQG*R*VVSDKMEKS*I*VVA*I*E*R*FVKHP*I*YGKF*I*K*R*TTKLH-$NHNH_2$	5164.2	SPPS attempt of S17 N-terminal half with HV NCL (Figure S42C)
S17_A24-L84	H_2N-C*I*E*R*FVKHP*I*YGKF*I*K*R*TTKLHVHDENNECG*I*GDVVE*I*REC*R*PLSKTKSWTLV*R*VVEKAVL-OH	7262.3	SPPS attempt of S17 C-terminal half with VA NCL (Figure S42D)
17	H_2N-TDK*I*RTLQG*R*VVSDKMEKSIV*V*-Nbz	2644.1	Intermolecular NCL of S17 (N'_S17, Figure S43A)
18	H_2N-C*I*E*R*FVKHP*I*YGKF*I*K*R*TTKLH-$NHNH_2$	2729.3	Intermolecular NCL of S17 (I'_S17, Figure S43B)
20	H_2N-TDK*I*RTLQG*R*VVSDK(Ddap-R-Mal)MEKSIV*V*-Nbz	3242.8	CAPTN of S17 (N_S17, Figure S45A)
21	H_2N-C(Stbu)*I*E*R*FVK(Ddap-R-C)HP*I*YGKF*I*K*R*TTK(Ddap-R-N_3)LH-$NHNH_2$	3912.9	CAPTN of S17 (I_S17, Figure S45B)
22	H_2N-Pen(SIT)HDE(AlHx-R-DBCO)NNEC(Acm)G*I*GDVVE*I*REC(Acm)*R*PLSK*T*KS*WT*LV*R*VVEKAVLHHHHHHA-OH	6248.2	CAPTN of S17 (C_S17, Figure S45C)
8c	H_2N-Pen(Stbu)GKENTWY-NH_2	1114.9	Pen(Stbu/SIT) removal studies (Figure S46A)
8d	H_2N-Pen(SIT)GKENTWY-NH_2	1129.4	Pen(Stbu/SIT) removal studies (Figure S46B)

Residues that are *italicized* were double coupled during SPPS. Underlined residues indicate the use of a pseudoproline during SPPS. N-termini of peptides 2a, 1, 2, 3, 1b, 1c, 2b, 2c, 4b, 1', 12, 13, 7a, 8a, 20, 21, 22, 8c, and 8d were protected with Boc prior to on-resin peptide derivatization (e.g. HH functionalization, SIT/Stbu conversion).

SECTION 4: Supplementary Figures and Schemes

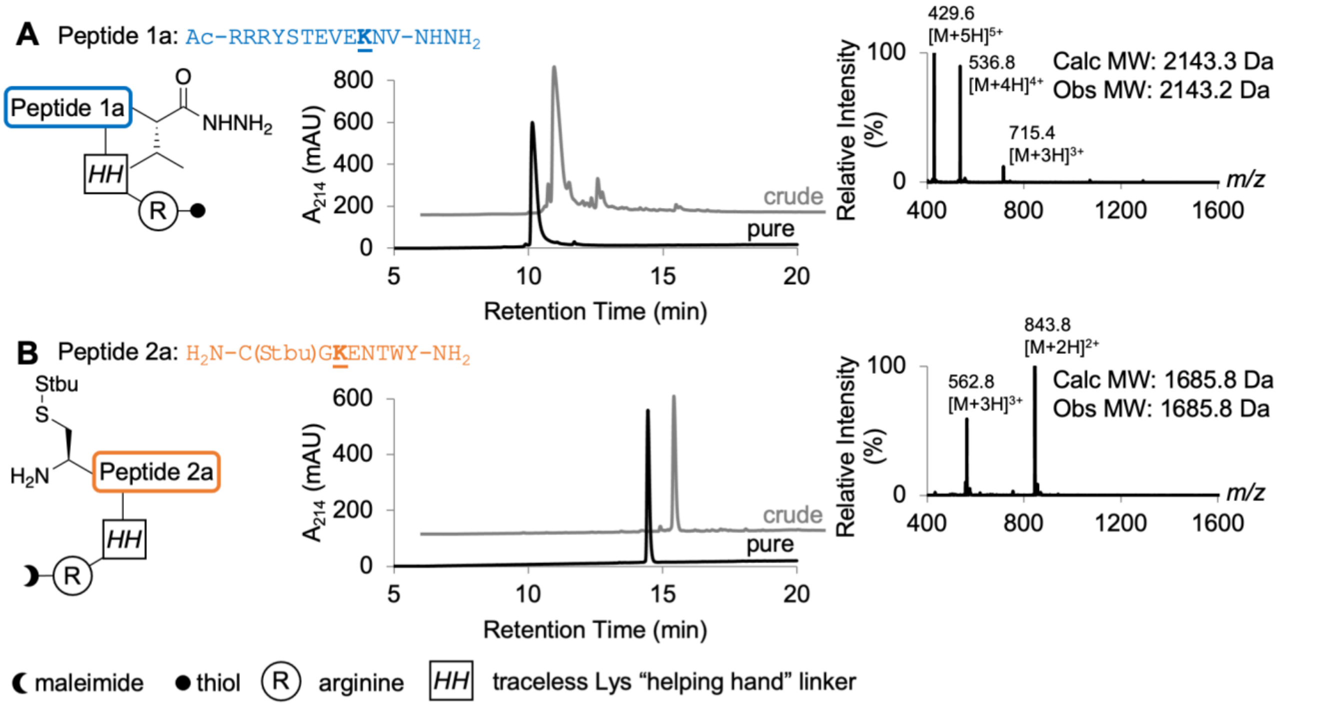

Figure S1. Peptides for maleimide/thiol-mediated CAN. (A) Peptide 1a-$NHNH_2$ was functionalized with a thiol-containing HH linker. This peptide was synthesized at 50 μmol scale, cleaved under standard 50 μmol scale conditions, and purified using purification method A with an isolated yield of 23% (24.6 mg). (B) Peptide 2a was functionalized with a maleimide-containing HH linker. This peptide was synthesized at 50 μmol scale, cleaved under standard 50 μmol scale conditions, and purified using purification method B with an isolated yield of 25% (21.1 mg). Analytical RP-HPLC method C and LC-MS method A were used for the analyses of pure and crude peptides. MS of the pure peptides taken from the entire LC-MS chromatograms are reported. Underlined and bolded Lys (**K**) indicate placements of HH linkers.

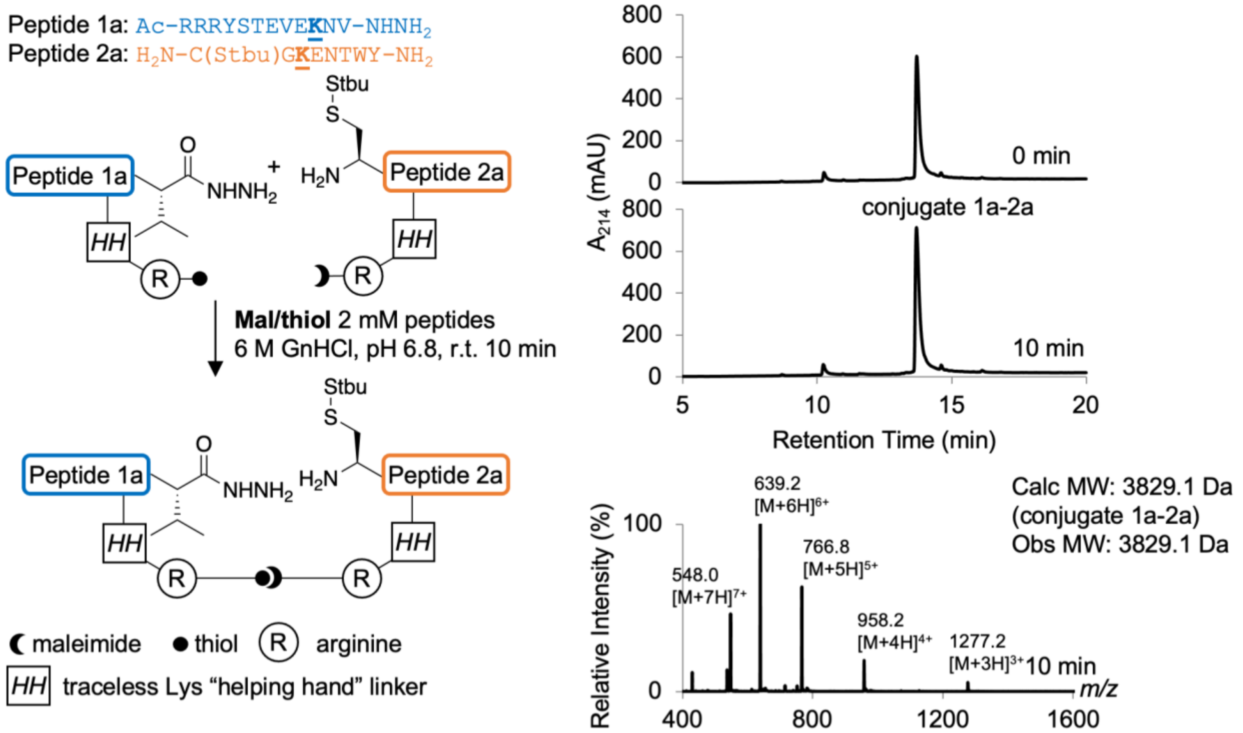

Figure S2. Maleimide/thiol conjugation reaction for CAN. Pure peptide 1a-NHNH$_2$ (Fig S1A) was conjugated to pure peptide 2a (Fig S1B). The reaction was monitored by analytical RP-HPLC using method C and LC-MS using method A. Clean and rapid conversion to product conjugate 1a-2a (**3a** in main text) was observed. MS from the entire LC-MS chromatogram is reported. Underlined and bolded Lys (**K**) indicate placements of HH linkers.

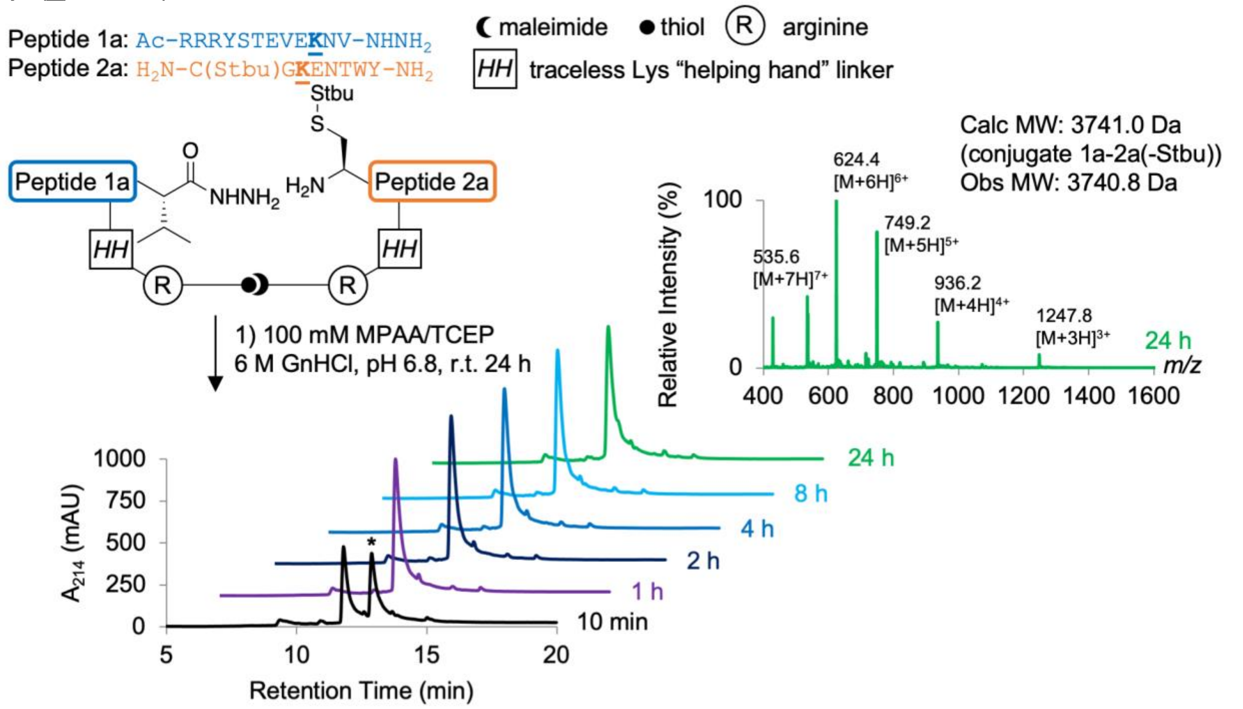

Figure S3. Maleimide/thiol conjugate stability. Stability of the peptide conjugate 1a-2a (**3a** in main text, Fig S2) without peptide-NHNH$_2$ activation was monitored over 24 h in NCL conditions (100 mM MPAA,100 mM TCEP, 6 M GnHCl, pH 6.8) by analytical RP-HPLC and LC-MS. The second peak (*) in the 10 min analytical RP-HPLC trace is conjugate with Stbu. No instability of the conjugate was observed. Analytical RP-HPLC method C and LC-MS method A were used for analysis. MS from the entire LC-MS chromatogram is reported. Underlined and bolded Lys (**K**) indicate placements of HH linkers.

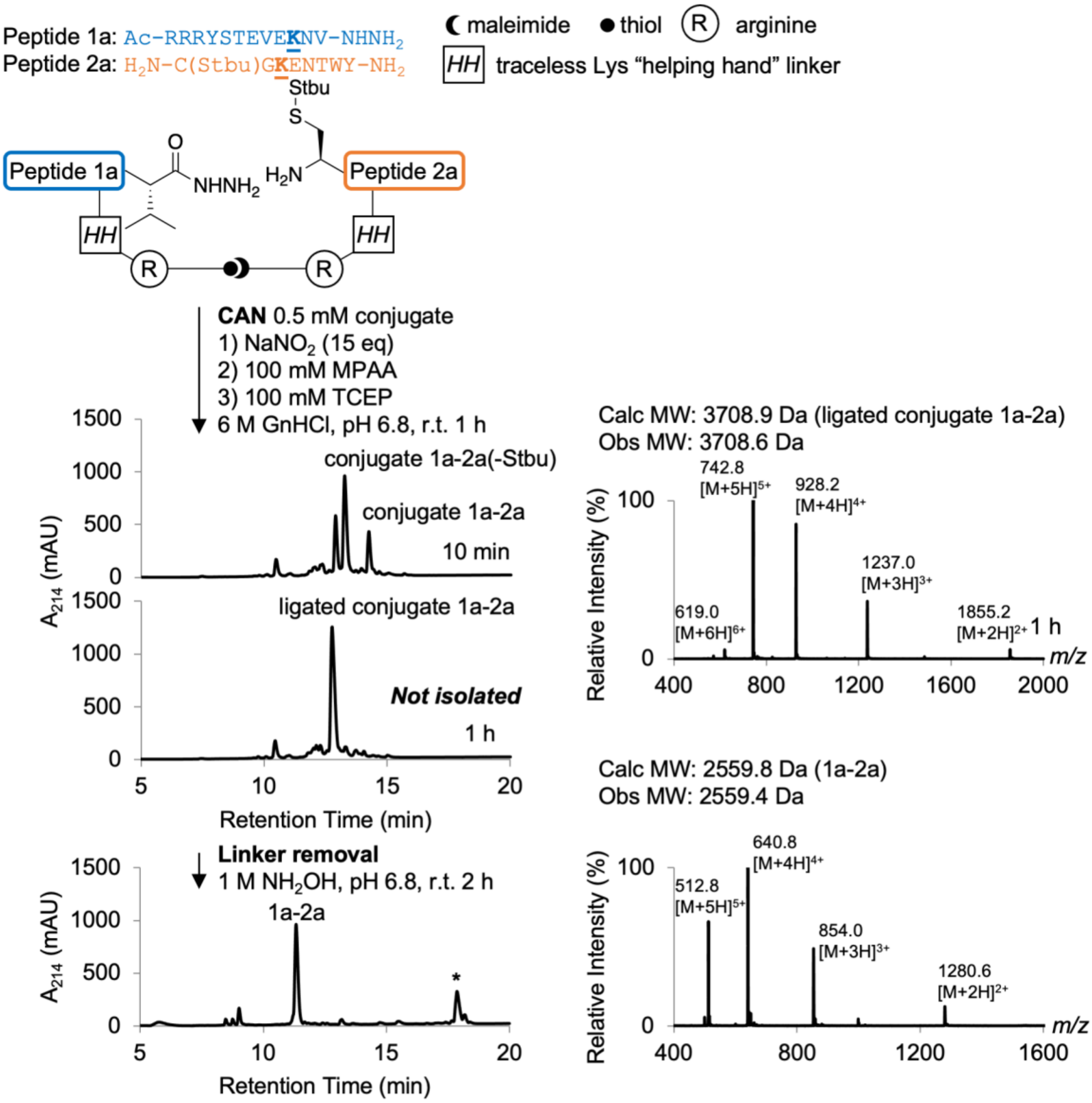

Figure S4. Maleimide/thiol-mediated CAN. Peptide conjugate 1a-2a (**3a** in main text, Fig S2) was ligated via peptide-$NHNH_2$ activation. Following CAN and HH linker removal, the desired product 1a-2a (**6a** in main text) was obtain demonstrating the utility of this second conjugation reaction needed for a 3-segment system. The (*) indicates the cleaved HH linkers. The analytical RP-HPLC traces are also shown in Fig 2. In addition to Fig 2, the MS of the individually labeled peaks from the analytical RP-HPLC chromatograms are reported. For the CAN reaction, the analytical RP-HPLC method C was used for analysis. Analytical RP-HPLC method F was used for the linker removal reaction. LC-MS method A was used for both reactions. Underlined and bolded Lys (**K**) indicate placements of HH linkers.

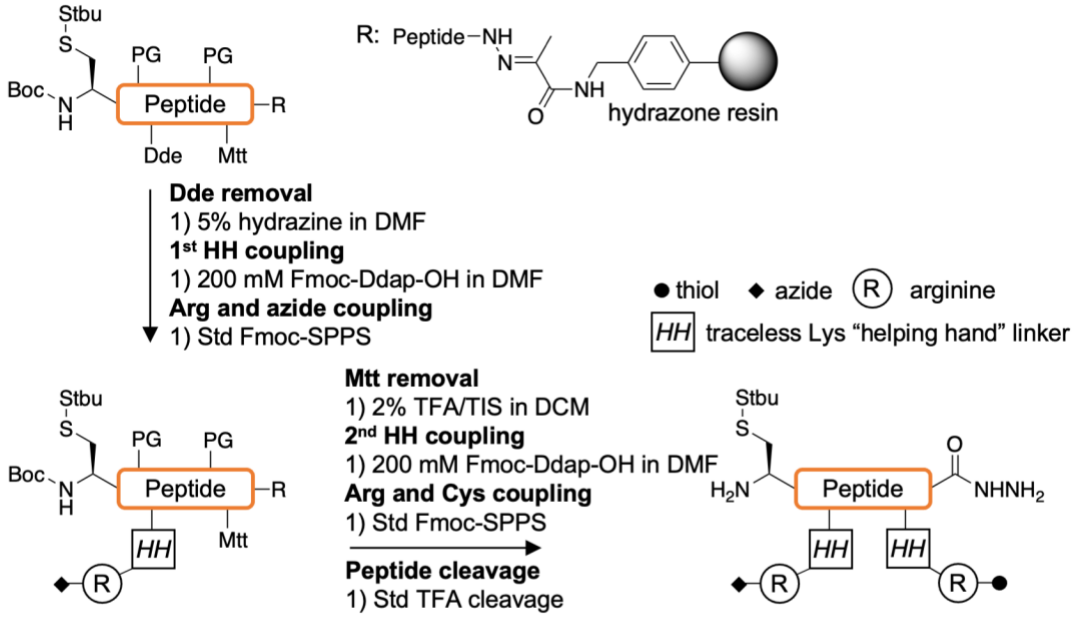

Figure S5: SPPS scheme for the synthesis of peptide-$NHNH_2$ with two functionalized HH linkers. Dde removal with 5% hydrazine enables selective coupling of the 1st HH followed by Arg and azide functionalization. Mtt removal with 2% TFA/TIS allows for the 2nd HH coupling and the functionalization with Arg and Cys (thiol). This synthesis was completed on hydrazone resin that is stable to low TFA conditions and yields a peptide-$NHNH_2$ post cleavage. Placement of Lys(Dde) and Lys(Mtt) during SPPS dictates the azide or thiol-functionalized HH linker positions. See method section for more detail.

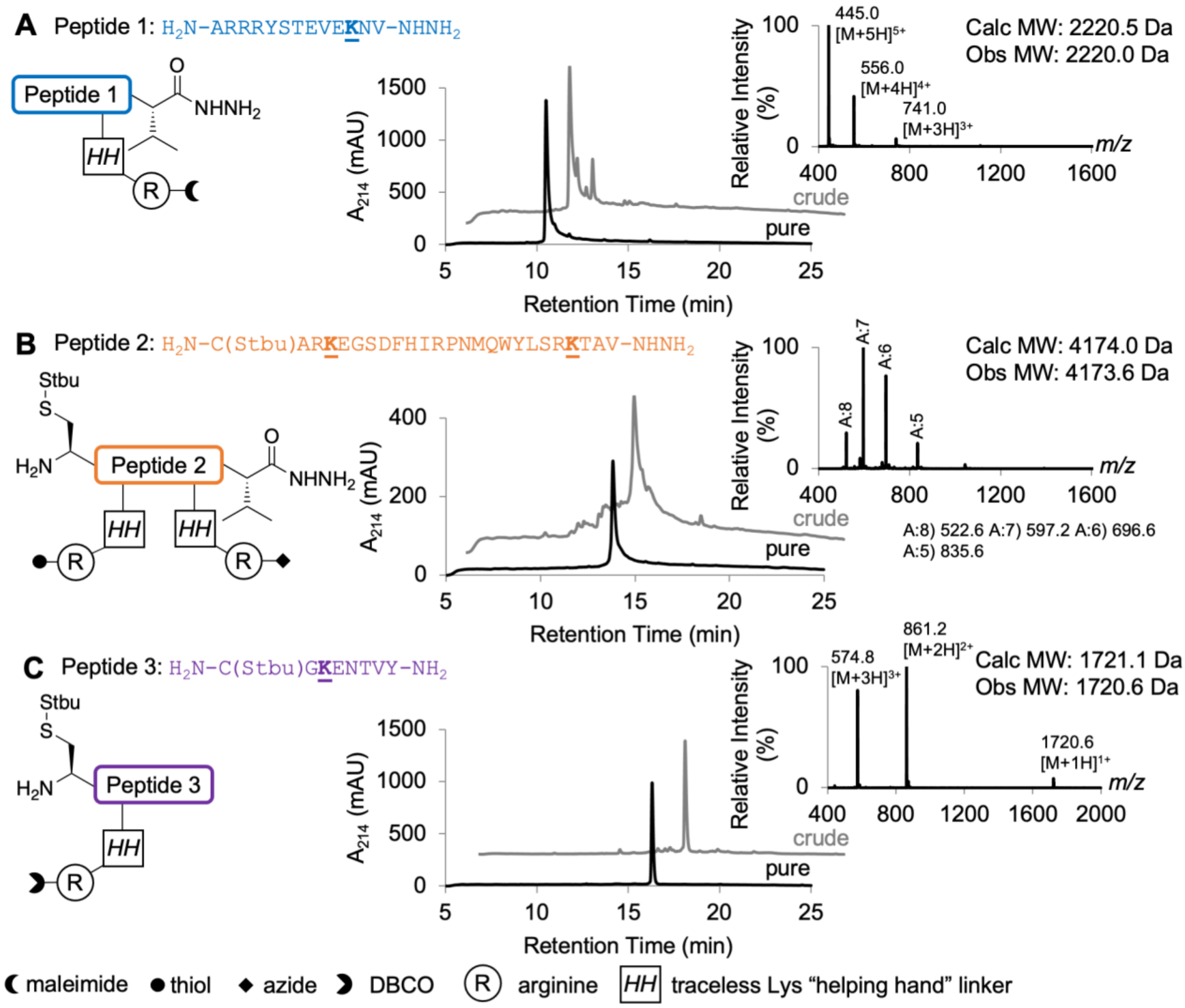

Figure S6: Peptides for the 3-segment templated NCL by peptide-$NHNH_2$ activation. (A) Peptide 1-$NHNH_2$ was functionalized with a maleimide-containing HH linker. This peptide was synthesized at a 50 µmol scale, cleaved under 50 µmol scale conditions, and purified using purification method L with an isolated yield of 22% (24.4 mg). Purity of peptide 1 was assessed by analytical RP-HPLC and LC-MS analysis. (B) Peptide 2-$NHNH_2$ was functionalized with a thiol-containing HH linker near its N-terminus and an azide HH linker near its C-terminus. This peptide was synthesized at a 25 µmol scale following steps described in Fig S5, cleaved under standard 25 µmol scale conditions, and purified using purification method D with an isolated yield of 18% (18.8 mg). Analytical RP-HPLC and LC-MS analysis were used to ensure purity. Note that this peptide contains all 20 canonical AAs other than Met substituted for Nle. (C) Peptide 3 was functionalized with a DBCO-containing HH linker. This peptide was synthesized at a 50 µmol scale, cleaved under standard 50 µmol scale conditions with Cu protection, and purified using purification method E with an isolated yield of 27% (23.2 mg). Purity of peptide 3 was assessed by analytical RP-HPLC and LC-MS analysis. Analytical RP-HPLC method A was used for the analyses of pure and crude peptides. LC-MS method A was used for peptide 1 and peptide 3 while method B was used for peptide 2. MS of the pure peptides taken from the entire LC-MS chromatograms are reported. Underlined and bolded Lys (**K**) indicate placements of HH linkers.

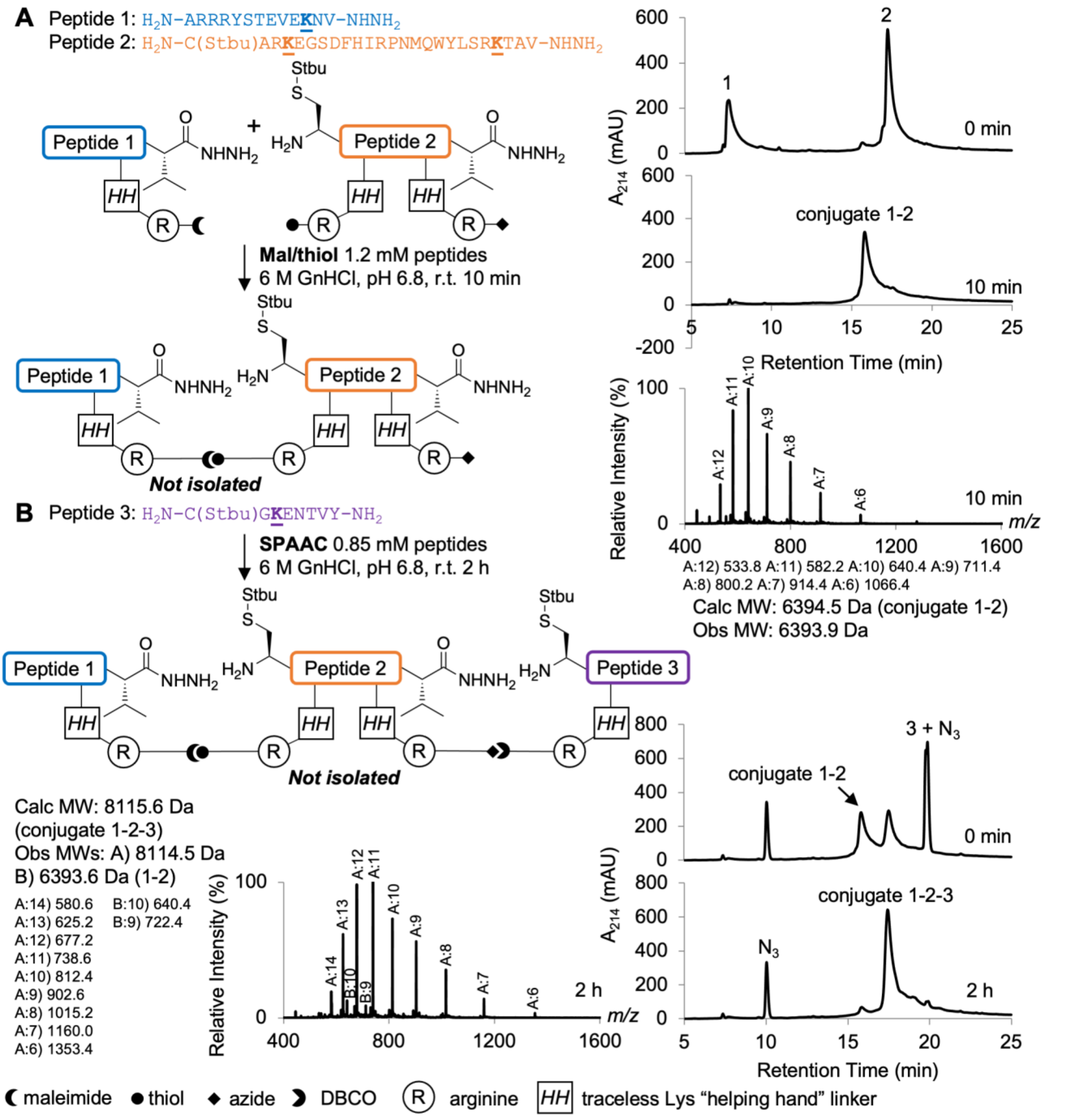

Figure S7. Conjugation reactions for the 3-segment templated NCL by peptide-NHNH2 activation. (A) Pure peptide 1-NHNH2 (Fig S6A) was conjugated to pure peptide 2-NHNH2 (Fig S6B). The reaction was monitored by analytical RP-HPLC and LC-MS. Conversion to product conjugate 1-2 (**4** in main text) was observed at 10 min. Note that the 0 min time point was conducted at pH 3 to catch the individual peptides. The pH was adjusted to 6.8 to initiate the conjugation. (B) Directly following the first conjugation reaction, conjugate 1-2 was clicked to pure peptide 3 (Fig S6C) by SPAAC. Reaction progression was monitored by analytical RP-HPLC and LC-MS. The time points were quenched with excess 6-azidohexanoic acid (N_3) to consume unreacted peptide 3-DBCO HH linker. The major product is the expected conjugate 1-2-3 (**5** in main text). Analytical RP-HPLC method B and LC-MS method B were used for the analyses. MS from the entire LC-MS chromatograms are reported. Underlined and bolded Lys (**K**) indicate placements of HH linkers. Nle was substituted for Met in peptide 2.

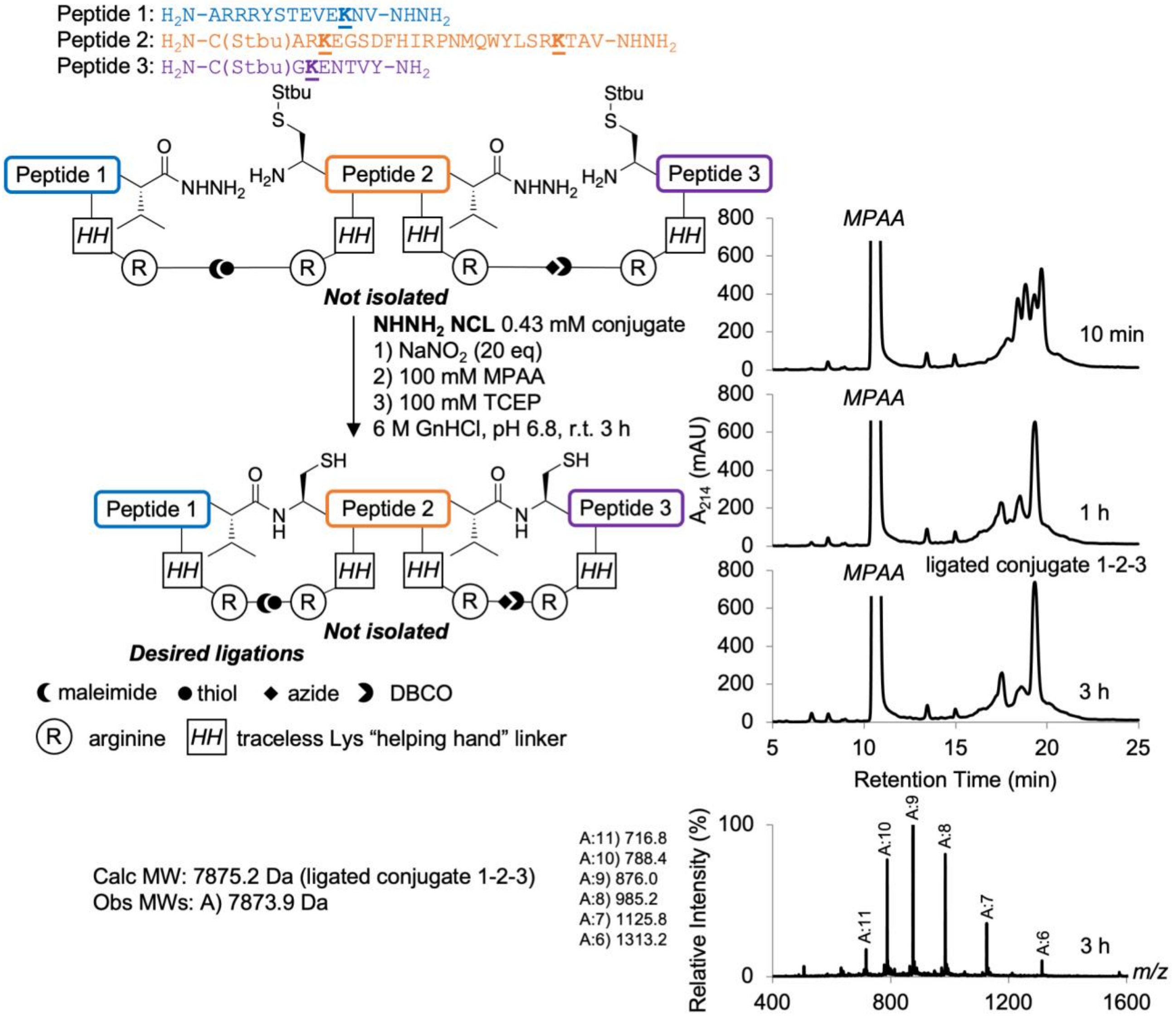

Figure S8. 3-segment templated NCL by peptide-NHNH$_2$ activation. Conjugate 1-2-3 (**5** in main text, Fig S7) was ligated in one-pot following conjugation by treatment with NaNO$_2$ at pH 3 to convert peptide 1-NHNH$_2$ and peptide 2-NHNH$_2$ simultaneously to thioesters via MPAA thiolysis. The reaction was initiated by raising the pH to 6.8. TCEP was then added to remove Stbu on both peptide 2 and peptide 3's N-terminal Cys. Time points of the ligation were taken over the course of 3 h and analyzed by analytical RP-HPLC and LC-MS. The reaction was completed in 3 h. Note that the analytical RP-HPLC traces contain multiple wide peaks while the MS of the 3 h time point is rather clean. This is because cross-ligations and desired ligations are occurring resulting in identical masses but different species with varying retention times by analytical RP-HPLC. Analytical RP-HPLC method B and LC-MS method B were used for the analysis. MS from the entire LC-MS chromatogram is reported. Underlined and bolded Lys (**K**) indicate placements of HH linkers. Nle was substituted for Met in peptide 2.

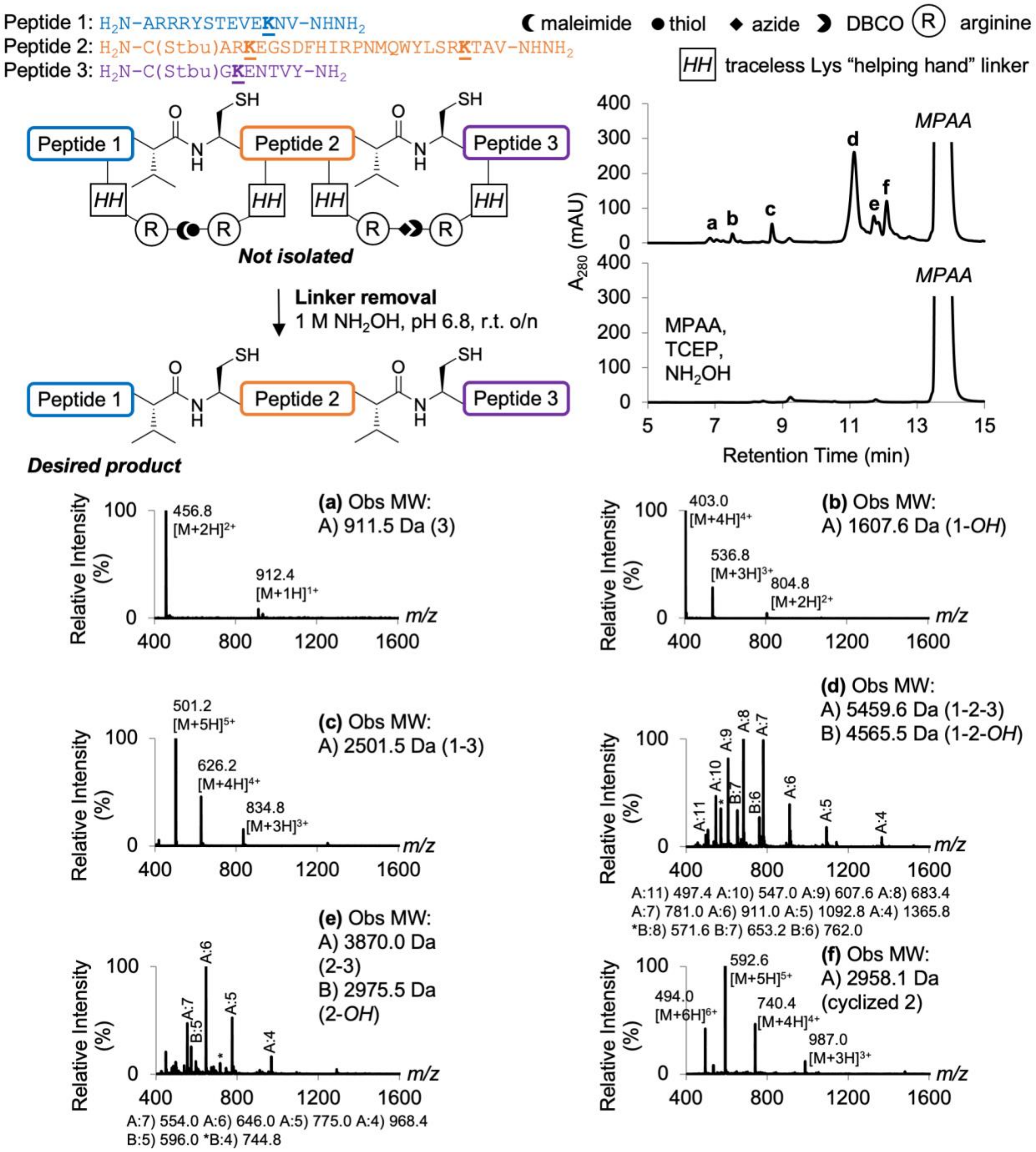

Figure S9. Linker removal from the 3-segment templated NCL by peptide-NHNH$_2$ activation. Ligated conjugate 1-2-3 (ligated **5** in main text, Fig S8) was treated with 1 M NH$_2$OH to cleave the HH linkers in one-pot following NCL completion. LC-MS analysis revealed the multiple products from the NCL reaction. While the major peak d is the desired ligated 1-2-3 peptide (**6** in main text, 70 % RP-HPLC yield), cross-ligation was observed yielding the side product 1-3 (peak c, **7** in the main text, 6.5 % RP-HPLC yield). Additionally, cyclization of the peptide 2 occurred (peak f, **8** in the main text, 18 % RP-HPLC yield). MPAA-related peaks were identified from an MPAA/TCEP/NH$_2$OH only injection. The LC chromatogram is also shown in Fig 3. LC-MS method C was used for the analysis. MS from the individually labeled peaks from the LC-MS chromatogram are reported. Underlined and bolded Lys (**K**) indicate placements of HH linkers. Nle was substituted for Met in peptide 2. Italicized *-OH* indicates thioester hydrolysis.

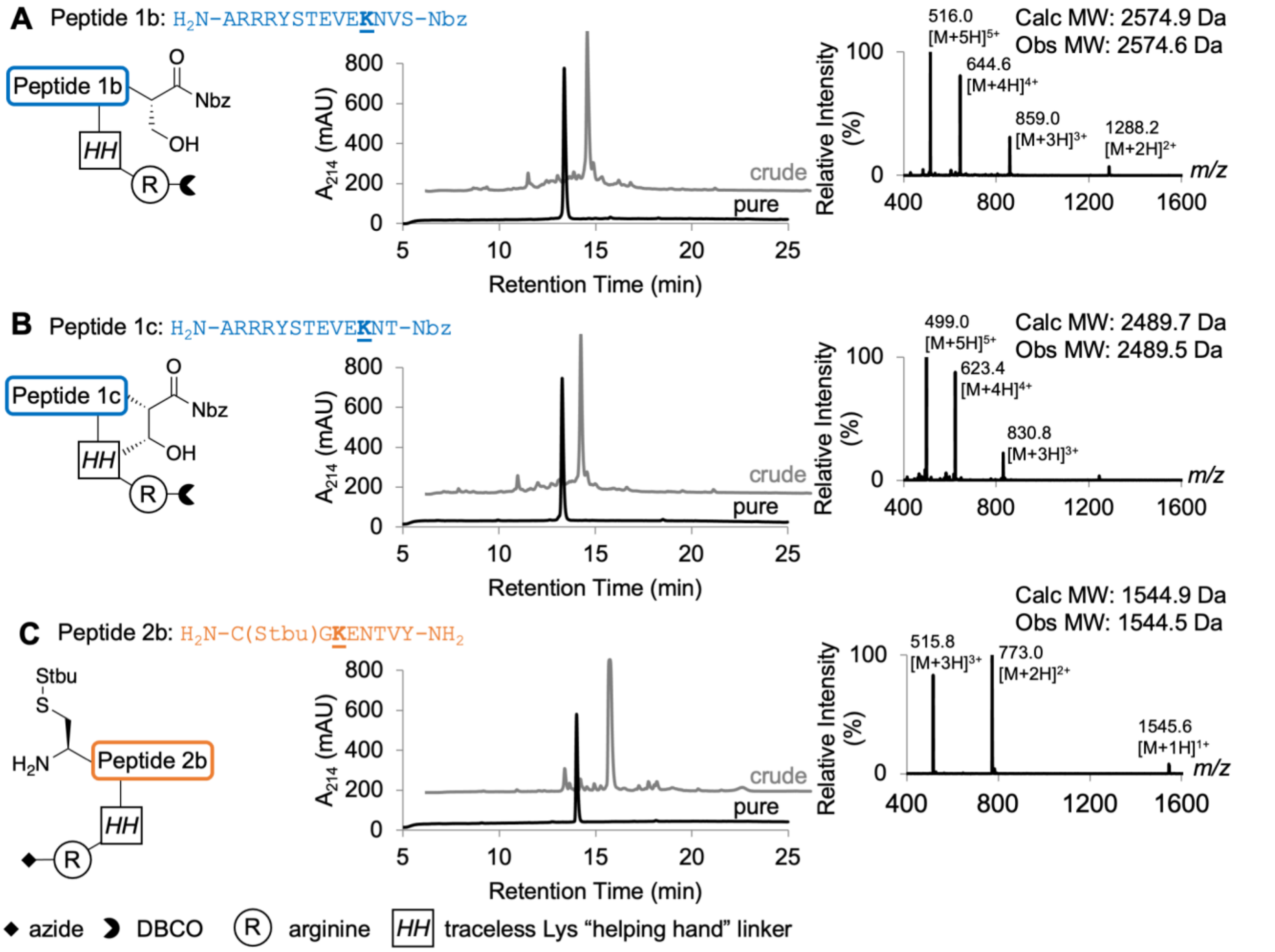

Figure S10: Peptides for the peptide-Nbz CAN experiments. (A) Peptide 1b-Nbz was functionalized with a DBCO-containing HH linker. The C-terminal residue Ser was used as a fast thioester. This peptide was synthesized at a 25 μmol scale, cleaved under 25 μmol scale conditions with Cu protection, and purified using purification method C with an isolated yield of 17% (10.9 mg). Purity of peptide 1b was assessed by analytical RP-HPLC and LC-MS analysis. (B) Peptide 1c-Nbz was functionalized with a DBCO-containing HH linker. The C-terminal residue Thr was used as a slow thioester. This peptide was synthesized at a 25 μmol scale, cleaved under 25 μmol scale conditions with Cu protection, and purified using purification method C with an isolated yield of 16% (10.0 mg). Purity of peptide 1c was assessed by analytical RP-HPLC and LC-MS analysis. (C) Peptide 2b was functionalized with an azide-containing HH linker. This peptide was synthesized at a 25 μmol scale, cleaved under standard 25 μmol scale conditions, and purified using purification method F with an isolated yield of 29% (11.2 mg). Purity of peptide 2b was assessed by analytical RP-HPLC and LC-MS analysis. Analytical RP-HPLC method A and LC-MS method A were used for the analyses of pure and crude peptides. MS of the pure peptides taken from the entire LC-MS chromatograms are reported. Underlined and bolded Lys (**K**) indicate placements of HH linkers.

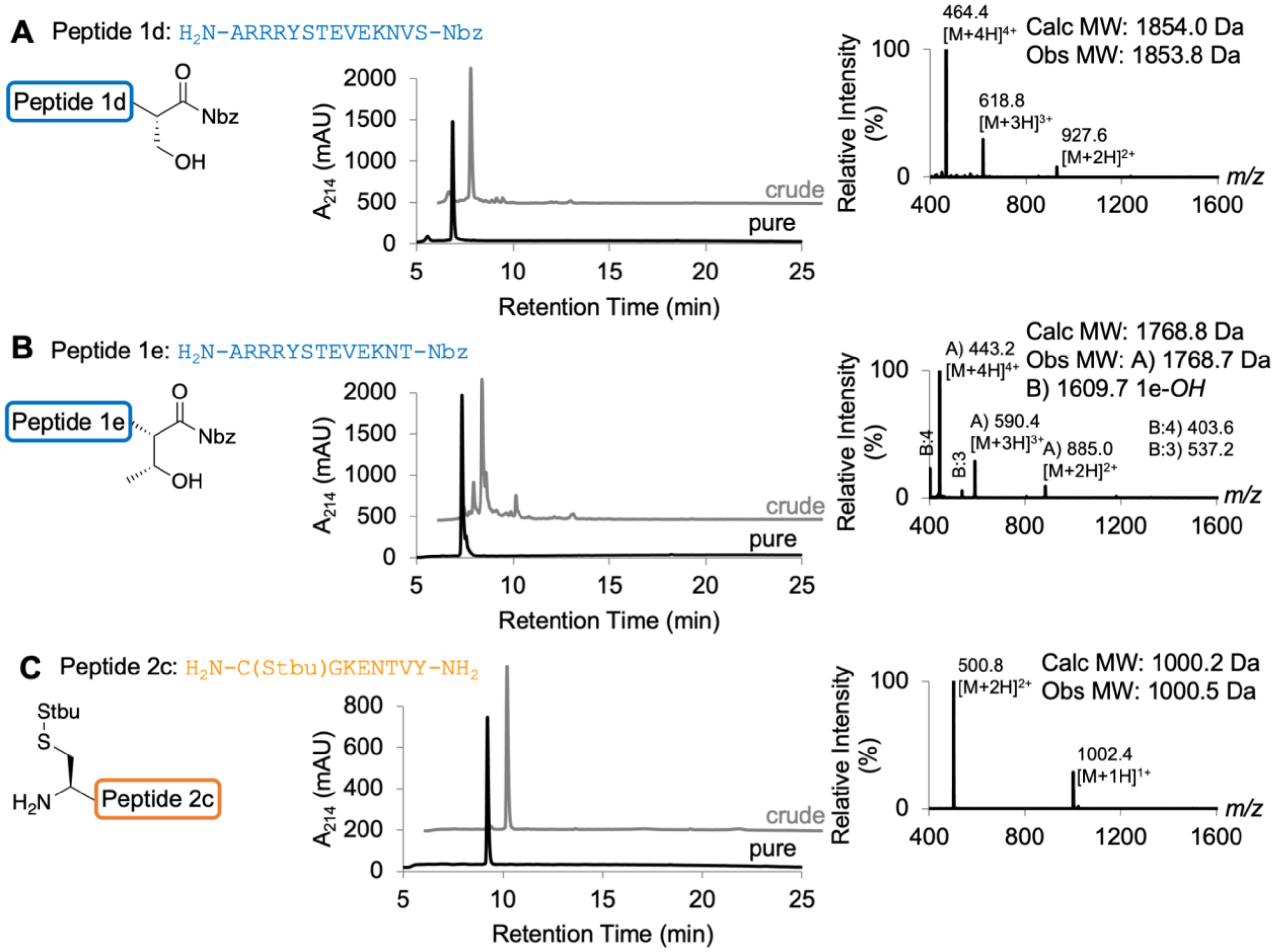

Figure S11: Peptides for the intermolecular NCL control. (A) Peptide 1d-Nbz was synthesized at a 25 µmol scale, cleaved under standard 25 µmol scale conditions, and purified using purification method J with an isolated yield of 23% (10.7 mg). The sequence matches peptide 1b (Fig S10A) including the C-terminal residue Ser as a fast thioester. Purity of peptide 1d was assessed by analytical RP-HPLC and LC-MS analysis. (B) Peptide 1e-Nbz was synthesized at a 25 µmol scale, cleaved under standard 25 µmol scale conditions, and purified using purification method I with an isolated yield of 22% (9.7 mg). The sequence matches peptide 1c (Fig S10B) including the C-terminal residue Thr as a slow thioester. Purity of peptide 1e was assessed by analytical RP-HPLC and LC-MS analysis. Note that some Nbz hydrolysis was observed by MS due to the sample sitting in RP-HPLC buffer prior to the analysis. (C) Peptide 2c was synthesized at a 25 µmol scale, cleaved under standard 25 µmol scale conditions, and purified using purification method K with an isolated yield of 30% (7.5 mg). The sequence matches peptide 2b (Fig S10C). Purity of peptide 2c was assessed by analytical RP-HPLC and LC-MS analysis. Analytical RP-HPLC method A and LC-MS method A were used for the analyses of pure and crude peptides. MS of the pure peptides taken from the entire LC-MS chromatograms are reported. Italicized *-OH* indicates peptide-Nbz hydrolysis.

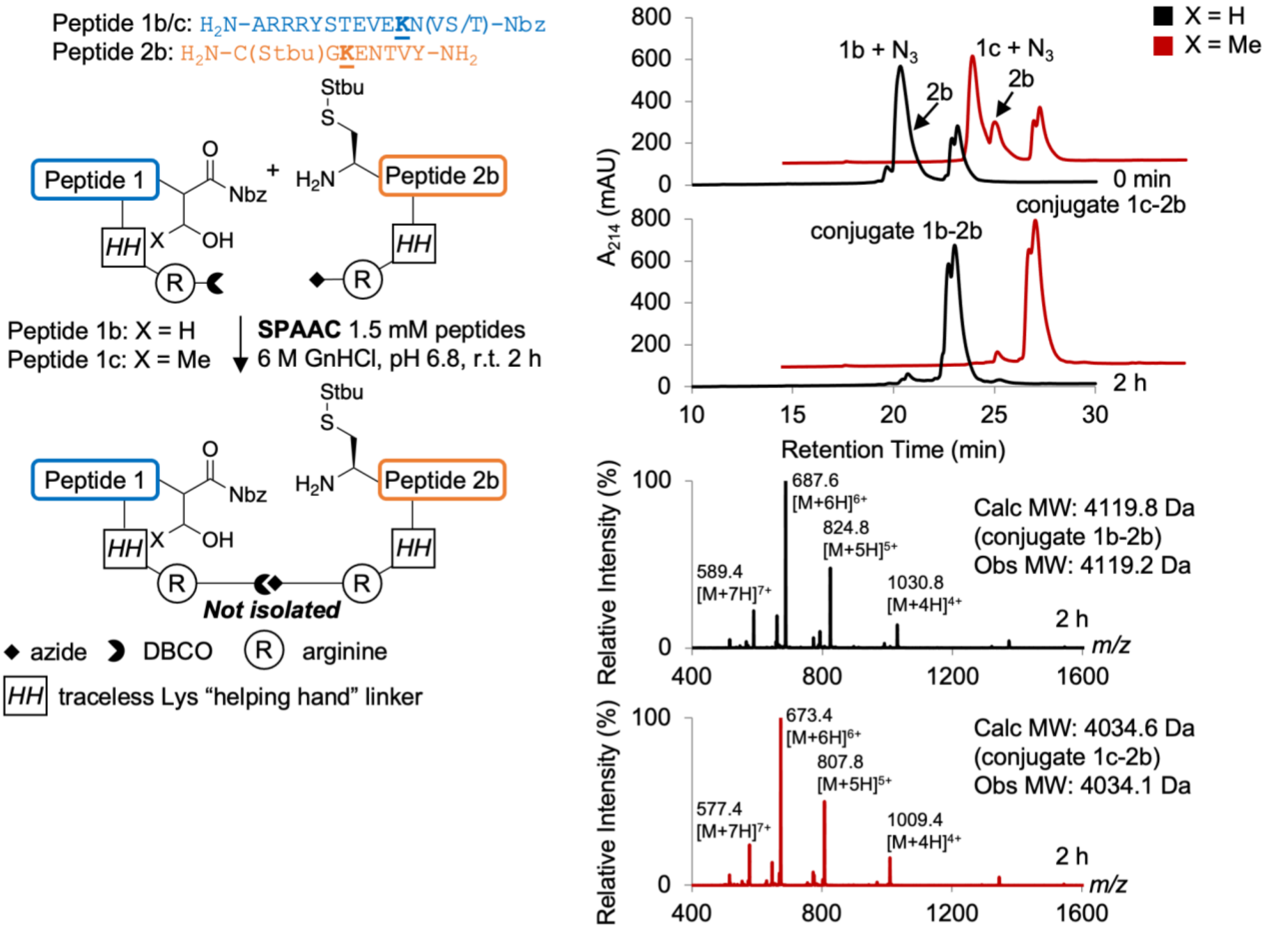

Figure S12: SPAAC reaction for the peptide-Nbz CAN experiments with fast and slow thioesters. Pure peptide 1b-Nbz (Fig S10A) and pure peptide 1c-Nbz (Fig S10B) were clicked to pure peptide 2b (Fig S10C). The time points were quenched with excess 6-azidohexanoic acid (N_3) to consume unreacted peptide 1b/c-DBCO HH linker. Clean conversion to product conjugates 1b-2b and 1c-2b was observed. Two peaks are expected due to the formation of regioisomers.[5] The reaction was monitored by analytical RP-HPLC using method D and LC-MS using method B. MS from the entire LC-MS chromatograms are reported. Underlined and bolded Lys (**K**) indicate placements of HH linkers.

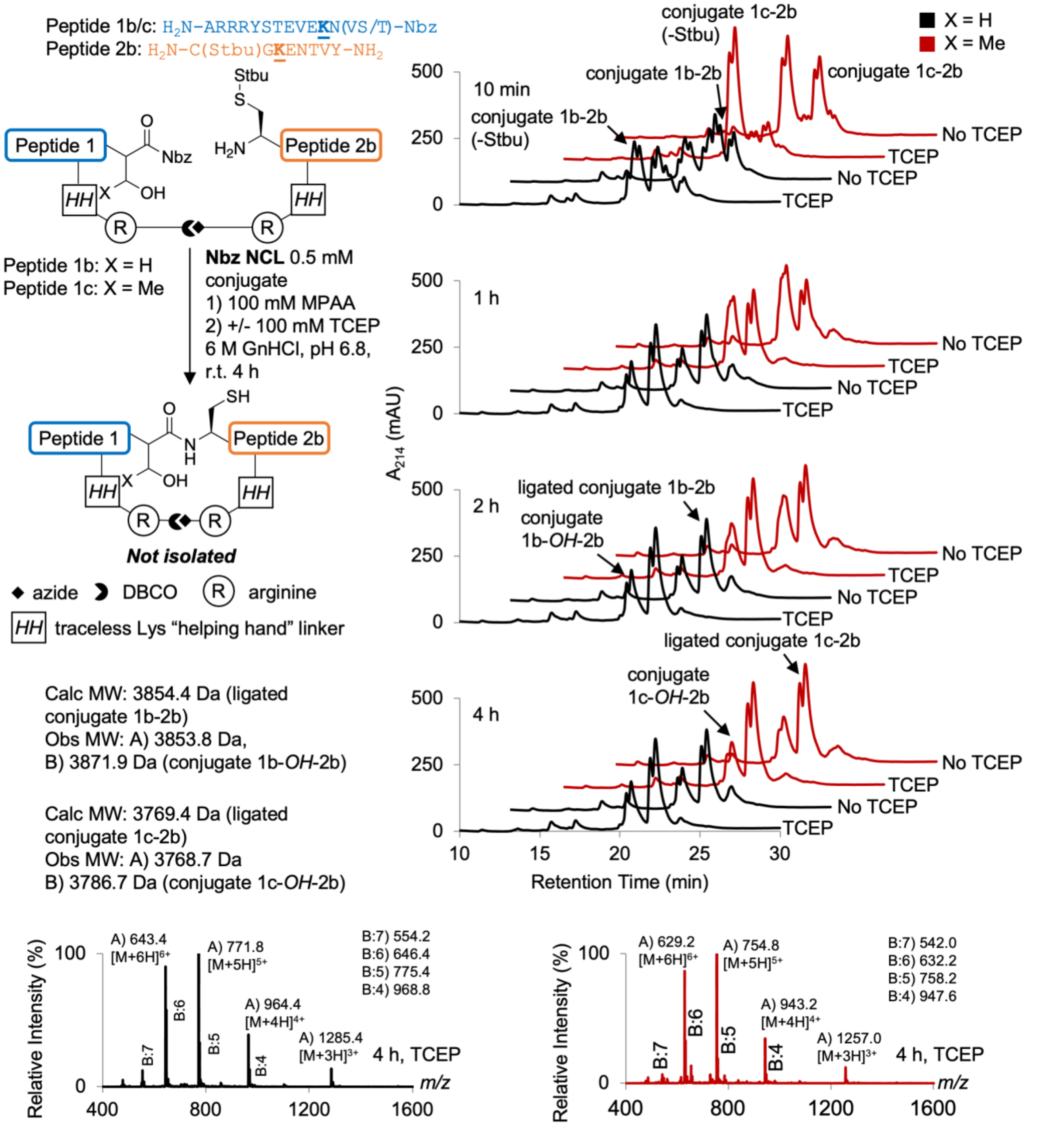

Figure S13: Peptide-Nbz CAN experiments with fast and slow thioesters. Peptide conjugates 1b-2b and 1c-2b (Fig S12) were ligated via peptide-Nbz activation. Two peaks are expected due to the formation of regioisomers during SPAAC.[5] Reaction conditions include 100 mM MPAA with or without 100 mM TCEP. Conversion to the ligated conjugates 1b-2b and 1c-2b was observed at 4 h with some hydrolyzed thioester. Comparable conversion with and without TCEP was observed. Analytical RP-HPLC method D and LC-MS method B were used for analysis. MS from the entire LC-MS chromatograms are reported. Underlined and bolded Lys (**K**) indicate placements of HH linkers. Italicized *-OH* indicates thioester hydrolysis.

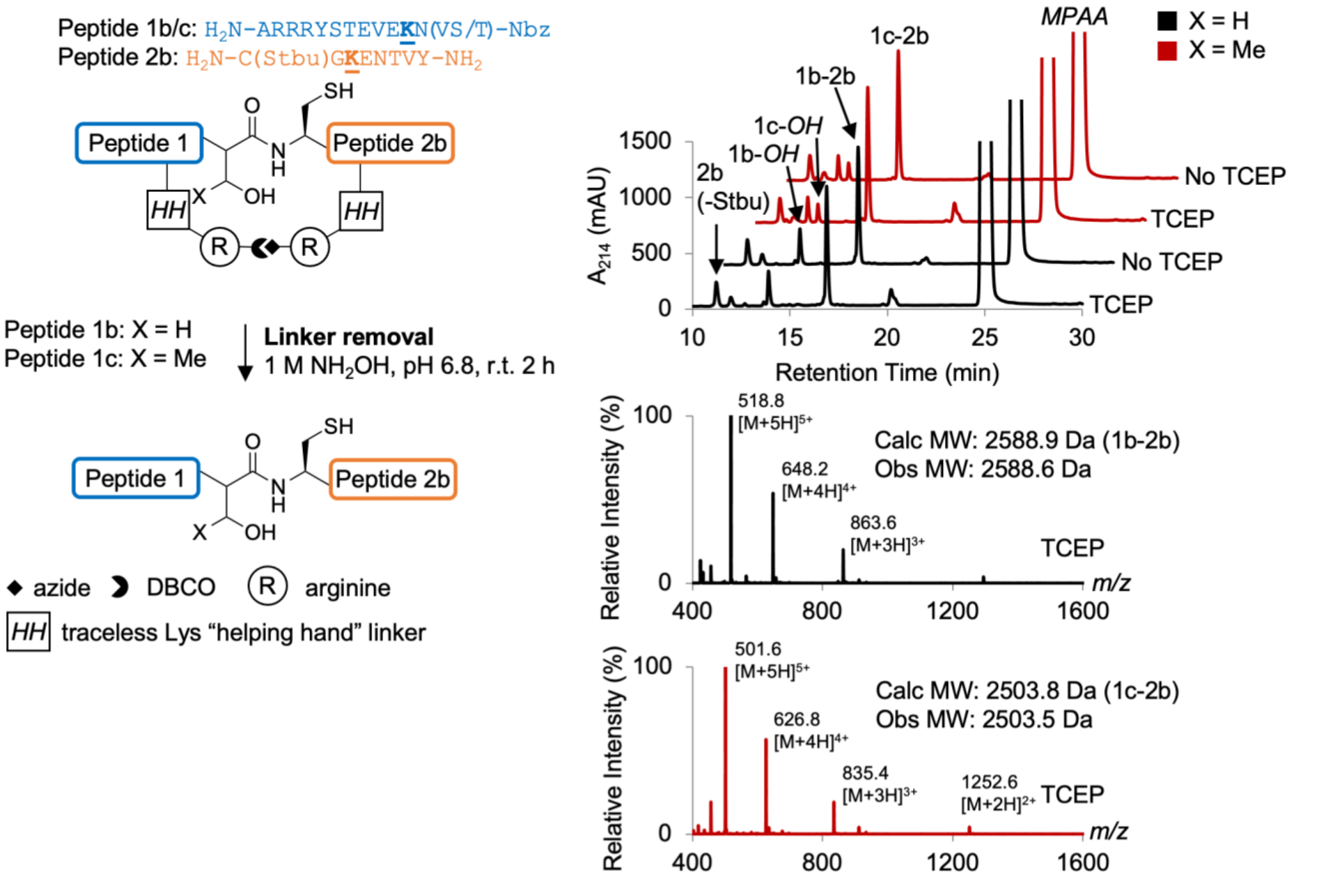

Figure S14. Linker removal from the peptide-Nbz CAN experiments with fast and slow thioesters. Ligated conjugates 1b-2b and 1c-2b from the peptide-Nbz CAN reactions (Fig S13) were treated with 1 M NH_2OH to cleave the HH linkers in one-pot following NCL completion. Analytical RP-HPLC and LC-MS analysis reveal the major products to be the desired ligated 1b-2b and 1c-2b peptides. Some unreacted starting material 2b was observed due to thioester hydrolysis (1b-*OH* and 1c-*OH*). The comparable results from the CAN reactions with and without TCEP suggest that TCEP does not play a considerable part in product formation. In addition, no incompatibilities with all step involved with CAN and peptide-Nbz chemistry were identified as results were comparable to the intermolecular control (Fig S15) although performing better (faster NCL completion). Analytical RP-HPLC method E and LC-MS method D were used for analysis. MS of the individually labeled product peaks from the analytical RP-HPLC chromatogram are reported. Underlined and bolded Lys (**K**) indicate placements of HH linkers. Italicized -*OH* indicates thioester hydrolysis.

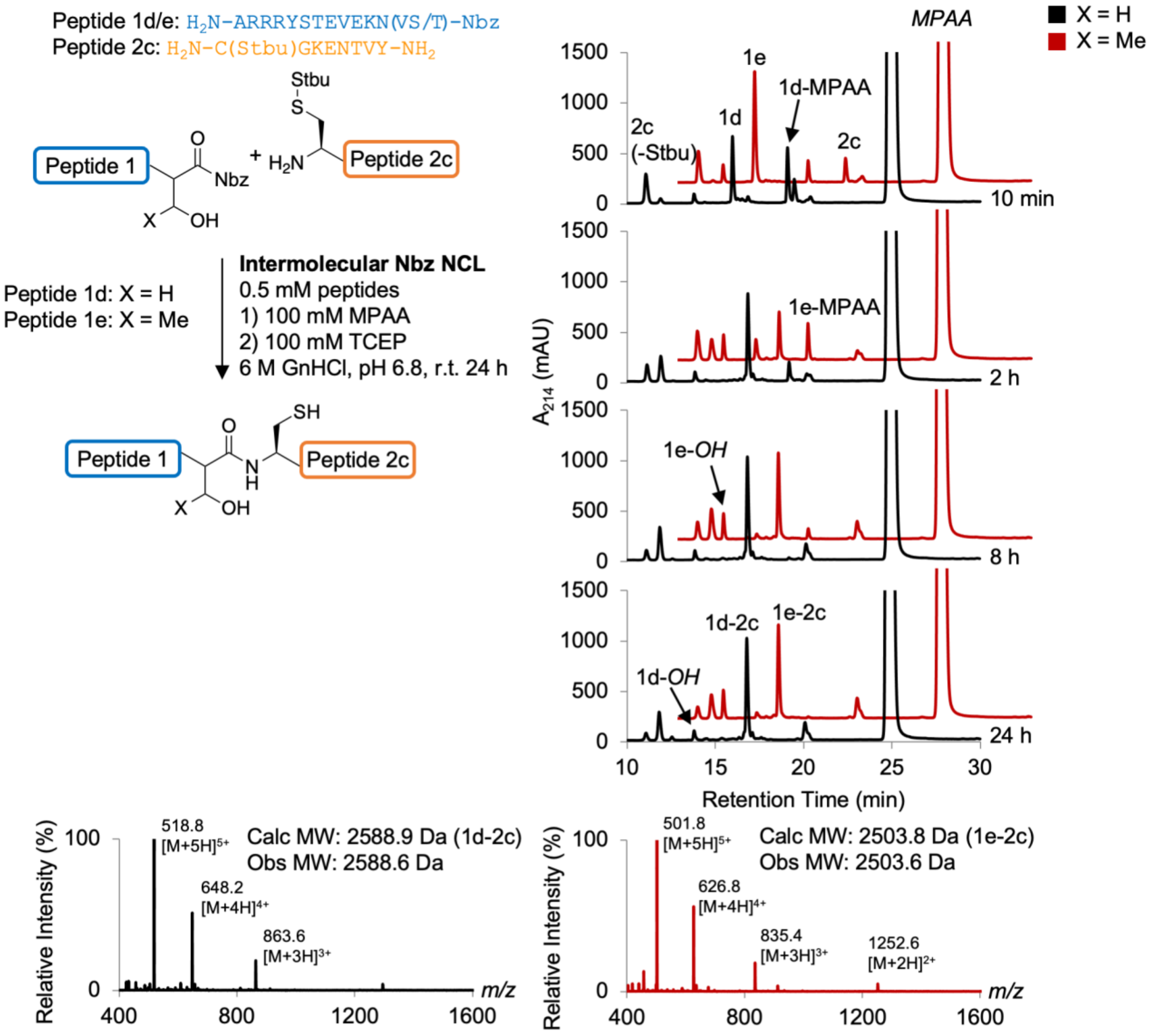

Figure S15. Intermolecular peptide-Nbz NCL control reactions with fast and slow thioesters. Peptide 1d-Nbz (Fig S11A) as well as peptide 1e-Nbz (Fig S11B) were ligated to peptide 2c (Fig S11C) via peptide-Nbz activation with 100 mM MPAA and 100 mM TCEP. Complete conversion to the ligated peptide 1d-2c was observed at 8 h and peptide 1e-2c at 24 h with some hydrolyzed thioester (1d-*OH* and 1e-*OH*). Analytical RP-HPLC method E and LC-MS method D were used for the analyses. MS of the individually labeled product peaks from the analytical RP-HPLC chromatogram are reported. Italicized -*OH* indicates thioester hydrolysis.

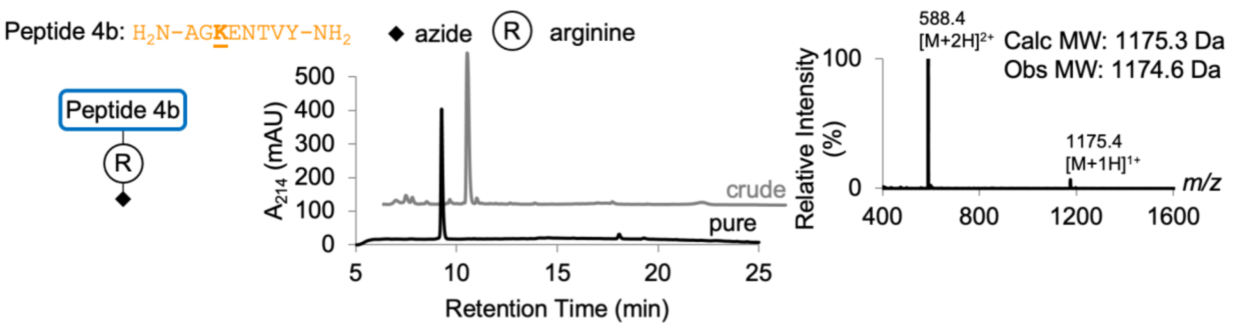

Figure S16: Peptide for azide stability assessment. Peptide 4b was functionalized with an azide. This peptide was synthesized at a 25 µmol scale, cleaved under 25 µmol scale conditions, and purified using purification method N with an isolated yield of 29% (8.5 mg). Purity of peptide 4b was assessed by analytical RP-HPLC and LC-MS analysis. Analytical RP-HPLC method A and LC-MS method A were used for the analysis of pure and crude peptide 4b. MS of the pure peptide taken from the entire LC-MS chromatogram is reported. Underlined and bolded Lys (**K**) indicates placements of the azide modification.

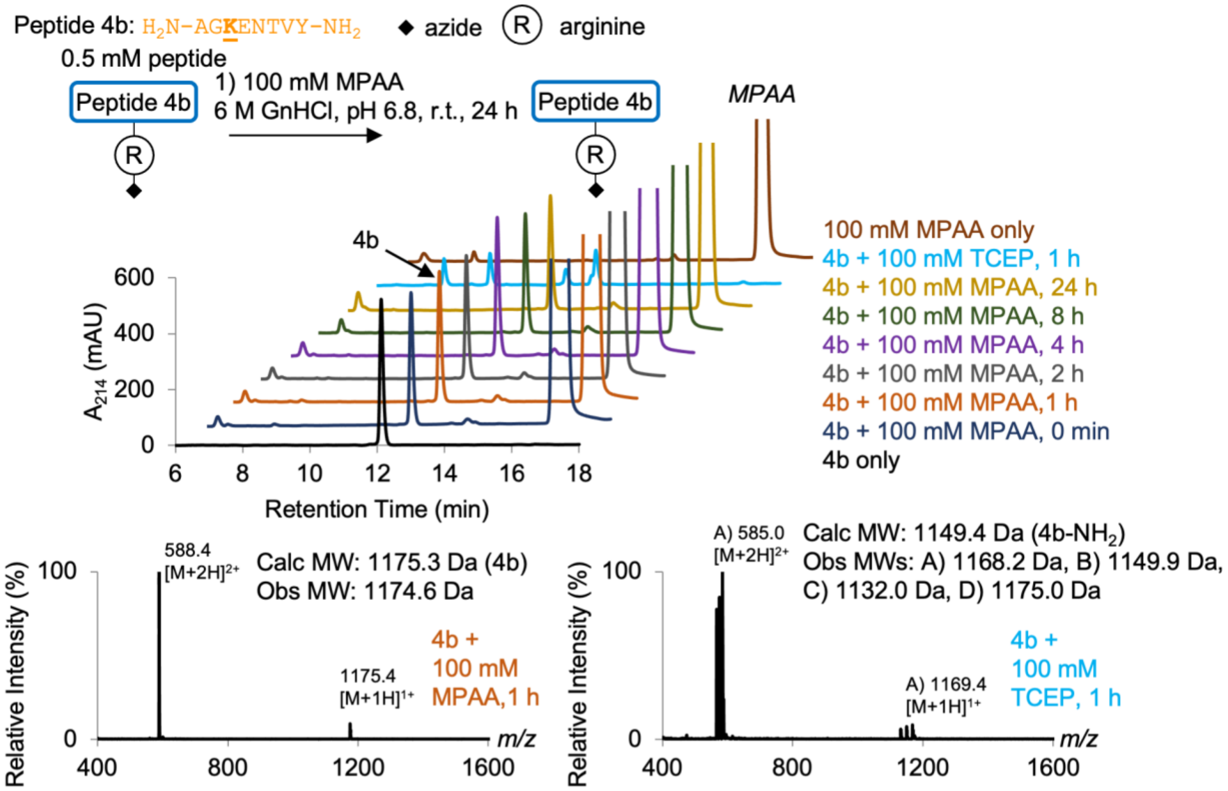

Figure S17. Azide stability assessment. To assess the stability of azide to the mild reducing peptide-Nbz NCL conditions containing MPAA, peptide 4b (Fig S16) was dissolved in NCL conditions (100 mM MPAA, 6 M GnHCl, pH 6.8) and the azide stability was monitored by analytical RP-HPLC and LC-MS. No azide instability was observed over 24 h ensuring suitability for one-pot SPAAC following peptide-Nbz NCL. In comparison, 100 mM TCEP resulted in side reactions including azide reduction after 1 h (light blue trace). Analytical RP-HPLC method G and LC-MS method A were used for the analysis. MS from the entire LC-MS chromatograms are reported.

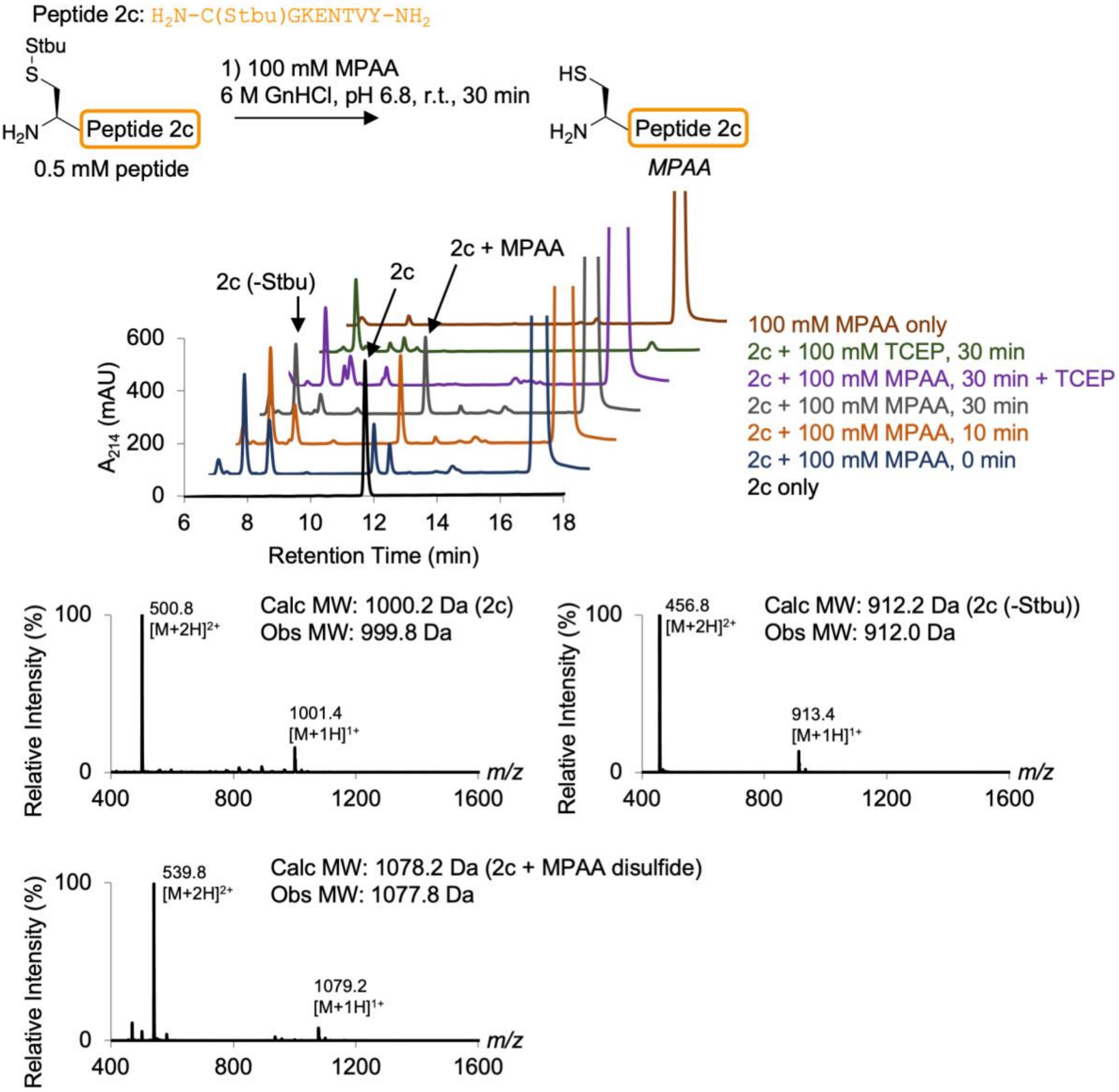

Figure S18. Stbu removal mediated by MPAA. To assess the removal of Stbu by MPAA in the absence of TCEP, peptide 2c (Fig S11C) was dissolved in NCL conditions (100 mM MPAA, 6 M GnHCl, pH 6.8) and the Stbu removal was monitored by analytical RP-HPLC and LC-MS. Complete Stbu removal was achieved after 10 min (orange trace) and comparable to treatment with 100 mM TCEP (green trace). An additional peak was observed during the MPAA-mediated removal and was identified by LC-MS to be a peptide-MPAA disulfide. Analytical RP-HPLC method G and LC-MS method A were used for the analysis. MS of the individually labeled peaks from the analytical RP-HPLC chromatogram are reported.

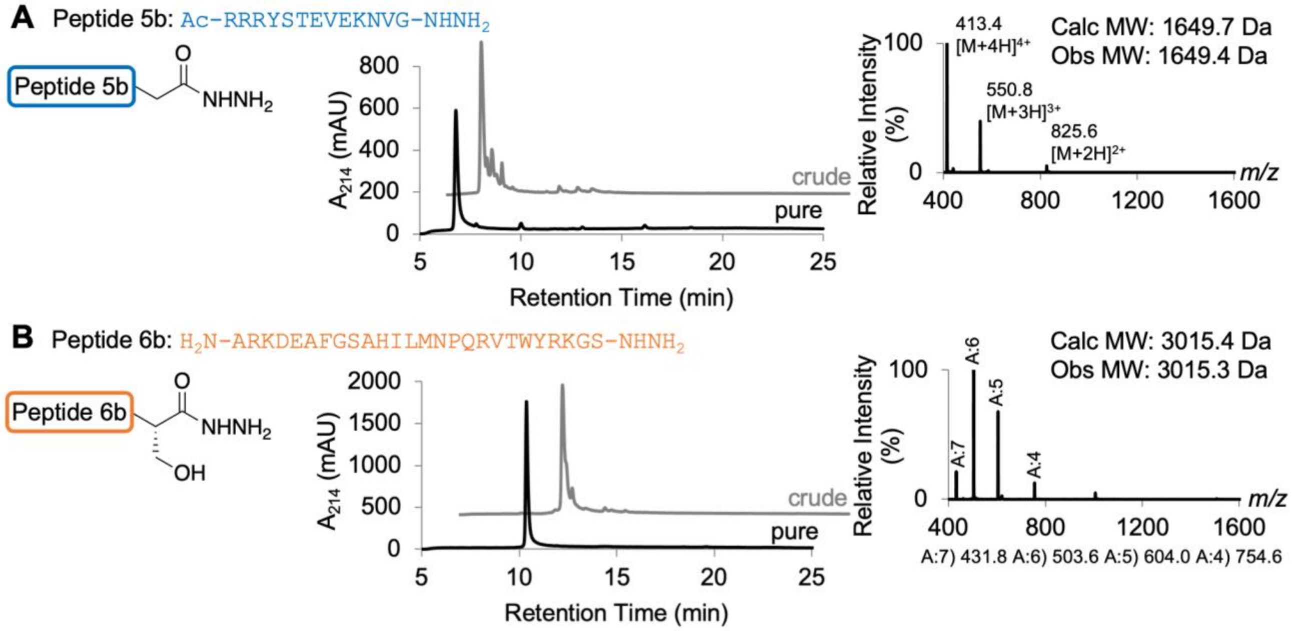

Figure S19: Peptides for peptide-NHNH$_2$ activation studies. (A) Peptide 5b-NHNH$_2$ was synthesized at a 25 µmol scale, cleaved under 25 µmol scale conditions, and purified using purification method M with an isolated yield of 30% (12.4 mg). Purity of peptide 5b was assessed by analytical RP-HPLC and LC-MS analysis. (B) Peptide 6b-NHNH$_2$ was synthesized at a 25 µmol scale, cleaved under standard 25 µmol scale conditions, and purified using purification method L with an isolated yield of 24% (18.1 mg). Analytical RP-HPLC and LC-MS analysis were used to ensure purity. Note that this peptide contains all 20 canonical AAs other than Met substituted for Nle. Analytical RP-HPLC method A and LC-MS method A were used for the analyses of pure and crude peptides. MS of the pure peptides taken from the entire LC-MS chromatograms are reported.

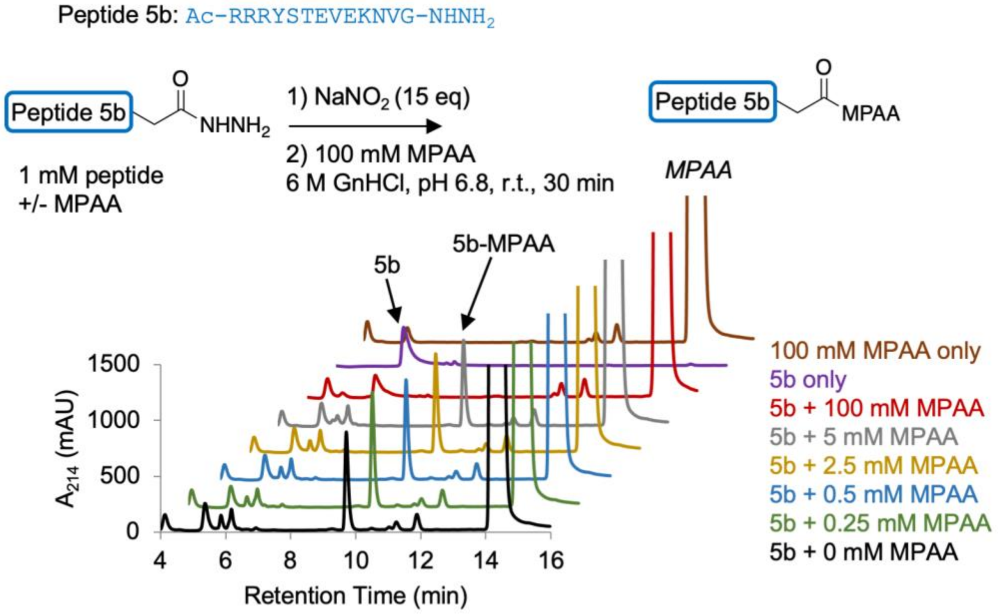

Figure S20. Inhibitory effect of MPAA on peptide-NHNH$_2$ activation. To assess the impact of MPAA on peptide-NHNH$_2$ activation, peptide 5b (Fig S19A) was pretreated with increasing concentrations of MPAA and activation to the thioester using standard NaNO$_2$ treatment and MPAA thiolysis was attempted. Analytical RP-HPLC analysis was conducted 30 min after MPAA addition and adjusting the pH to 6.8. Comparable conversion to the control (0 mM MPAA pretreatment, black trace) was achieved when pretreated with low mM MPAA while complete inhibition was observed with the 100 mM MPAA pretreatment (red trace). Analytical RP-HPLC method F was used for the analysis.

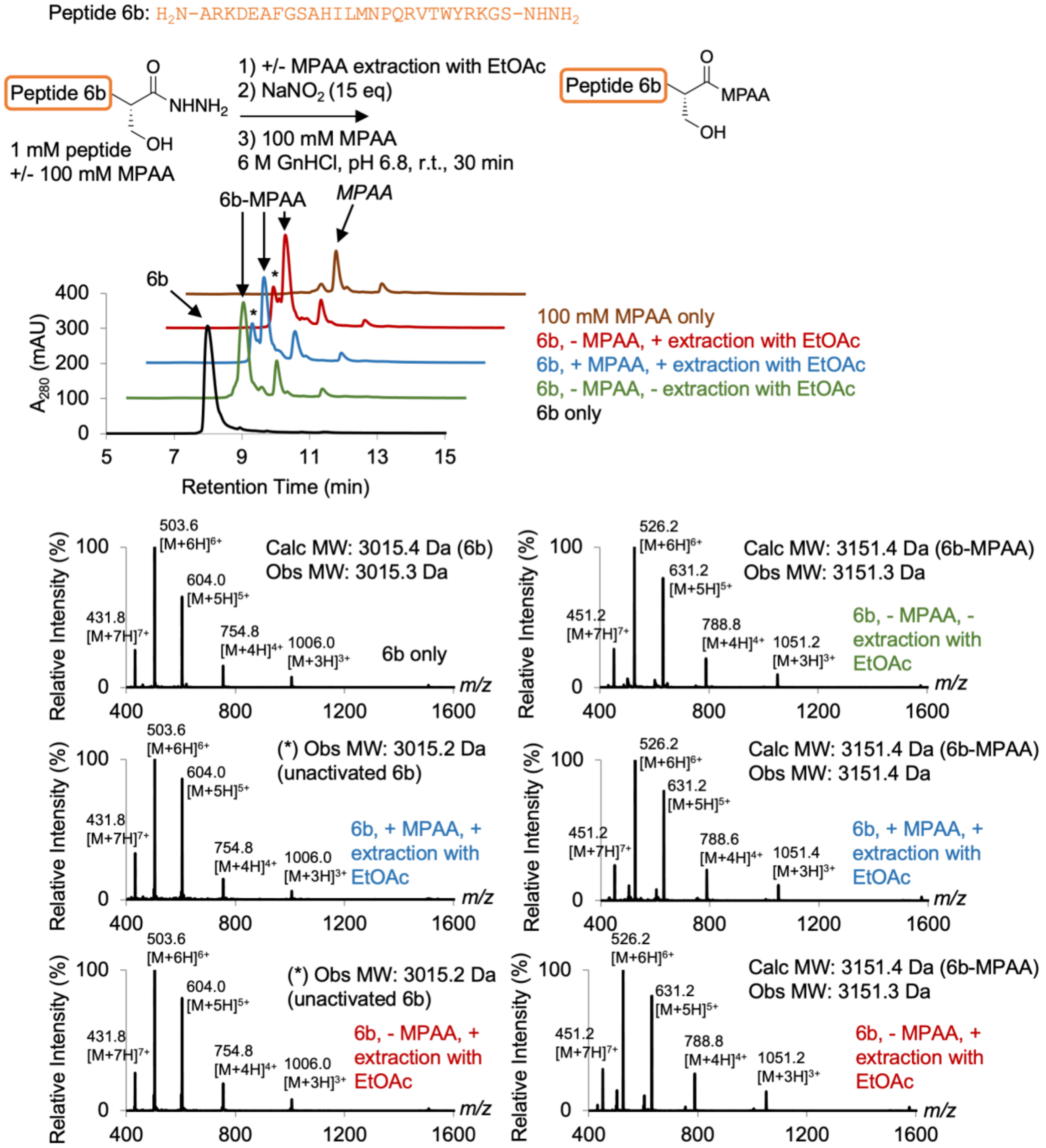

Figure S21. MPAA extraction with EtOAc for peptide-NHNH$_2$ activation. Peptide 6b-NHNH$_2$ (Fig S19B) was pretreated with 100 mM MPAA. The MPAA was extracted using EtOAc (see method section for more detail) and activation to the thioester using standard NaNO$_2$ treatment and MPAA thiolysis was attempted. Pretreatment resulted in incomplete conversion to the thioester as compared to the non-pretreated reaction. Similarly, incomplete conversion to the thioester was observed when performing an EtOAc extraction without pretreatment with MPAA suggesting that EtOAc extraction is preventing proper activation. LC-MS analysis was conducted 30 min after MPAA addition and adjusting the pH to 6.8. LC-MS method E was used for the analysis. MS from the individually labeled peaks from the LC-MS chromatogram are reported. Nle was substituted for Met in peptide 6b.

Peptide 6b: H_2N-ARKDEAFGSAHILMNPQRVTWYRKGS-NHNH$_2$

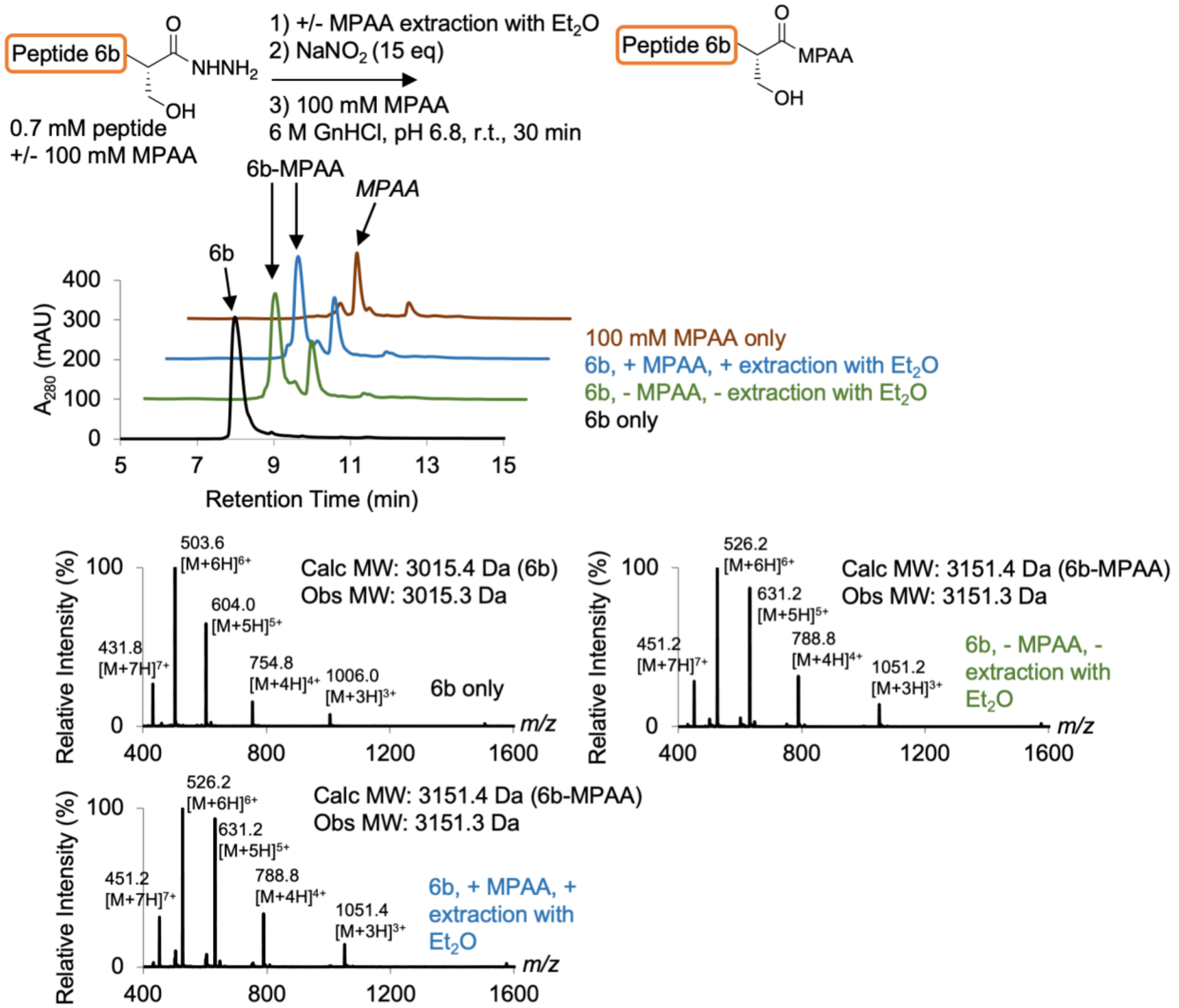

Figure S22. MPAA extraction with Et_2O for peptide-NHNH$_2$ activation. Peptide 6b-NHNH$_2$ (Fig S19B) was pretreated with 100 mM MPAA. The MPAA was extracted using Et_2O (see method section for more detail) and activation to the thioester using standard $NaNO_2$ treatment and MPAA thiolysis was attempted. Conversion to the thioester is comparable to the control when sample was pretreated with 100 mM MPAA and extracted with Et_2O. No incomplete activation was observed by LC-MS analysis unlike when the extraction was performed with EtOAc (Fig S21). LC-MS analysis was conducted 30 min after MPAA addition and adjusting the pH to 6.8. LC-MS method E was used for the analysis. MS from the individually labeled peaks from the LC-MS chromatogram are reported. Nle was substituted for Met in peptide 6b.

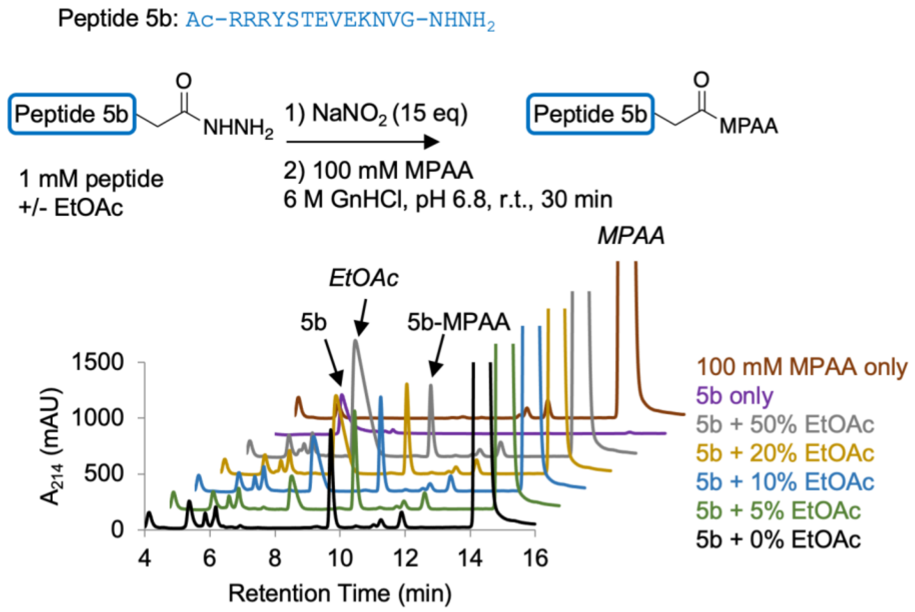

Figure S23. Impact of EtOAc on peptide-NHNH2 activation. Peptide 5b (Fig S19A) was treated with increasing amounts of EtOAc (%v/v) and standard activation to the thioester was conducted with $NaNO_2$ treatment and MPAA thiolysis. Activation in the presence of EtOAc is comparable to the control (0% EtOAc, black trace) suggesting that residual EtOAc from extraction is likely not the cause for incomplete activation seen in Fig S21. A slight decrease in activation was observed when treated with 50% EtOAc. Analytical RP-HPLC method F was used for the analysis.

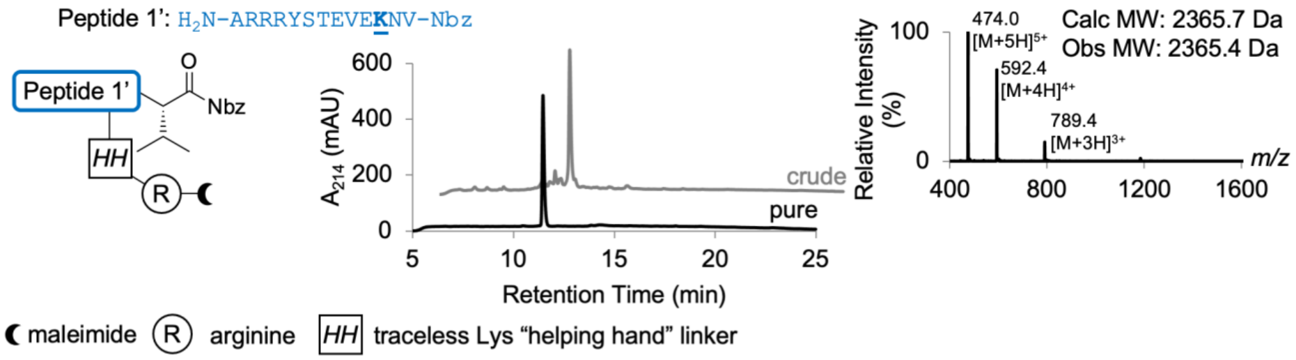

Figure S24: Peptide for CAPTN. Peptide 1'-Nbz was functionalized with a maleimide-containing HH linker. The sequence (other than -Nbz vs. -NHNH2) and HH functionalization match peptide 1 (Fig S6A). This peptide was synthesized at a 25 µmol scale, cleaved under standard 25 µmol scale conditions, and purified using purification method G with an isolated yield of 20% (11.8 mg). Purity of peptide 1' was assessed by analytical RP-HPLC and LC-MS. Analytical RP-HPLC method A and LC-MS method A were used for the analysis of pure and crude peptide 1'. MS of the pure peptide taken from the entire LC-MS chromatogram is reported. Underlined and bolded Lys (**K**) indicate placements of the HH linker.

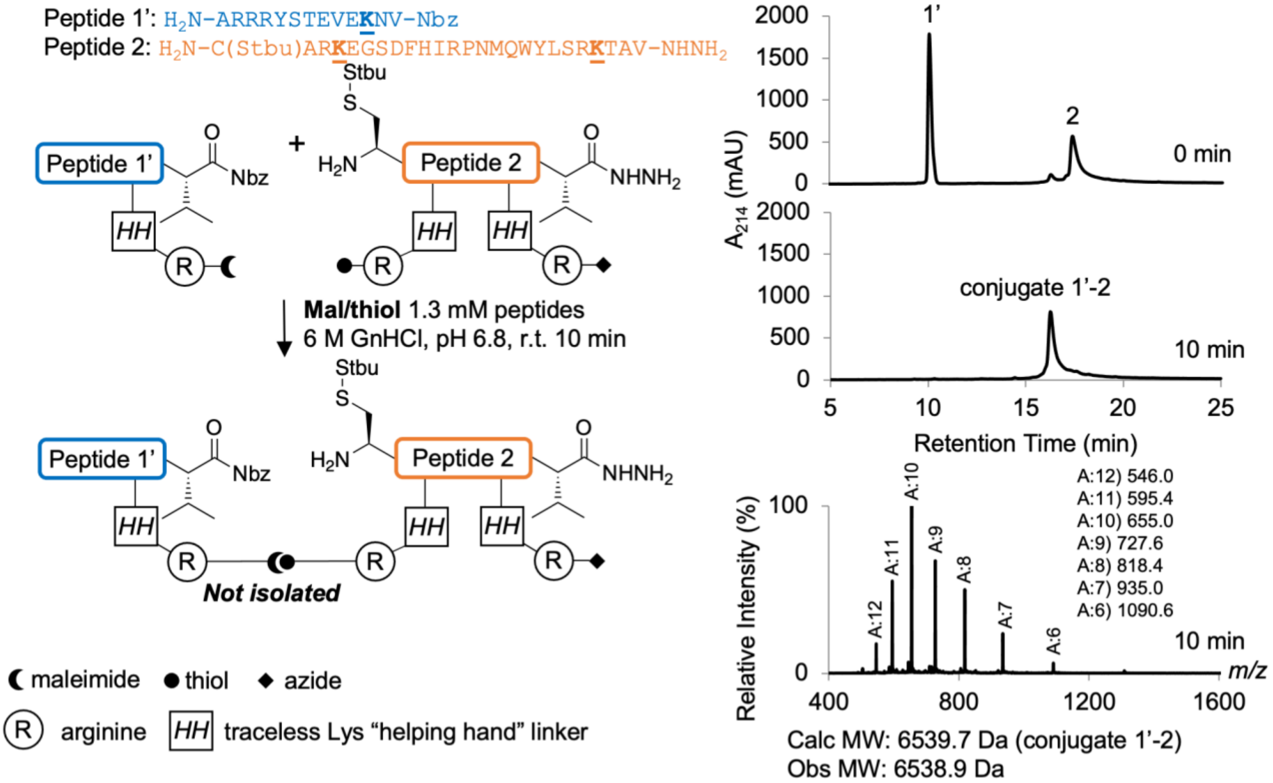

Figure S25. Conjugation reaction for CAPTN. Pure peptide 1'-Nbz (Fig S24) was conjugated to pure peptide 2-$NHNH_2$ (Fig S6B). The reaction was monitored by analytical RP-HPLC and LC-MS. Conversion to product conjugate 1'-2 was achieved in 10 min. Note that the 0 min time point was conducted at pH 3 to catch the individual peptides. The pH was adjusted to 6.8 to initiate the conjugation. Analytical RP-HPLC method B and LC-MS method B were used for the analyses. MS from the entire LC-MS chromatogram is reported. Underlined and bolded Lys (**K**) indicate placements of HH linkers. Nle was substituted for Met in peptide 2.

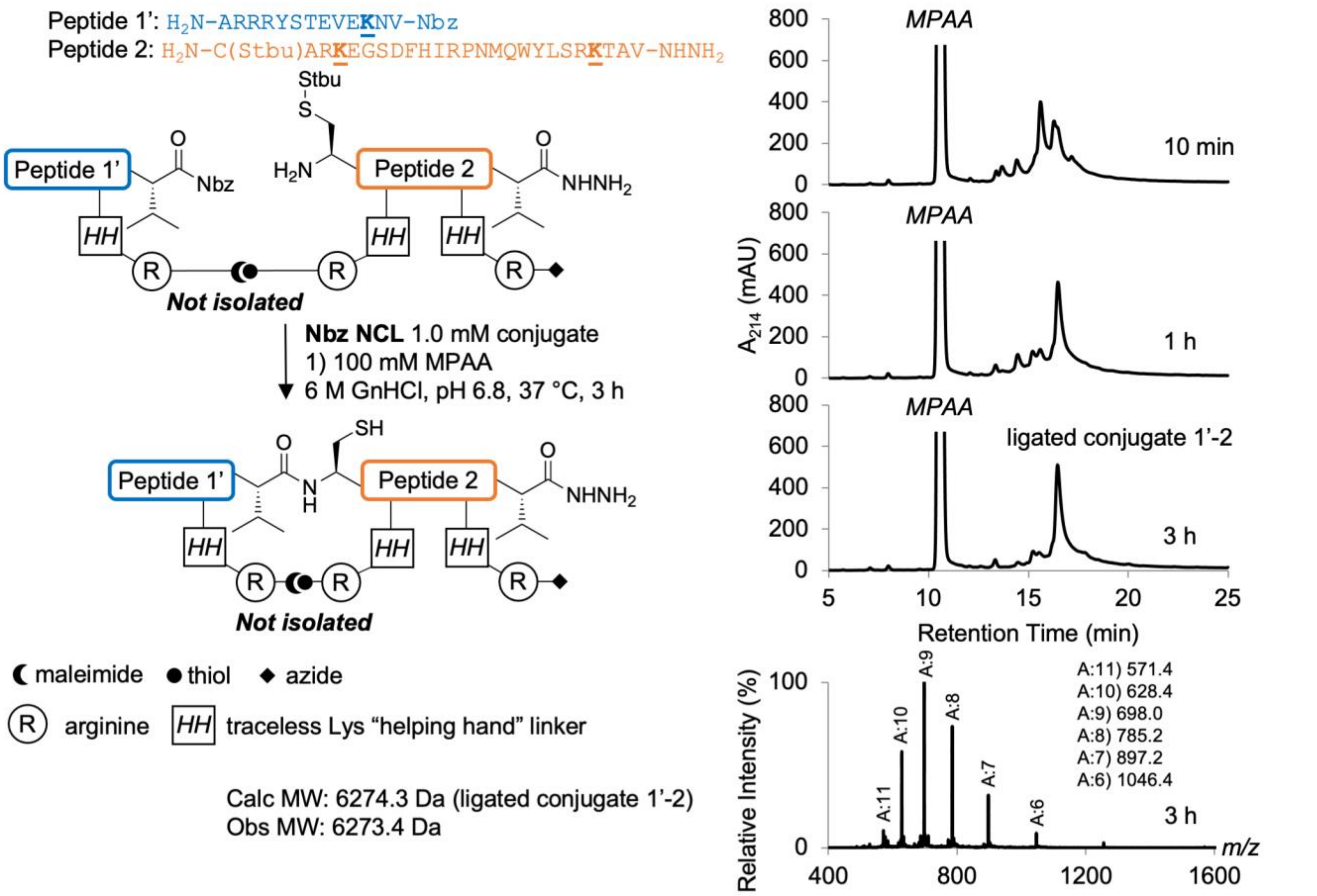

Figure S26. Peptide-Nbz ligation for CAPTN. Conjugate 1'-2 (Fig S25) was ligated in one-pot following conjugation by treatment with 100 mM MPAA at pH 6.8 to convert peptide 1'-Nbz to the thioester while maintaining peptide 2 as a peptide-NHNH$_2$. Time points of the ligation were taken over the course of 3 h and analyzed by analytical RP-HPLC and LC-MS. Conversion to the ligated conjugate (**4'** in main text) was observed at the 3 h time point with minimal thioester hydrolysis. Analytical RP-HPLC method B and LC-MS method B were used for the analyses. MS from the entire LC-MS chromatogram is reported. Underlined and bolded Lys (**K**) indicate placements of HH linkers. Nle was substituted for Met in peptide 2.

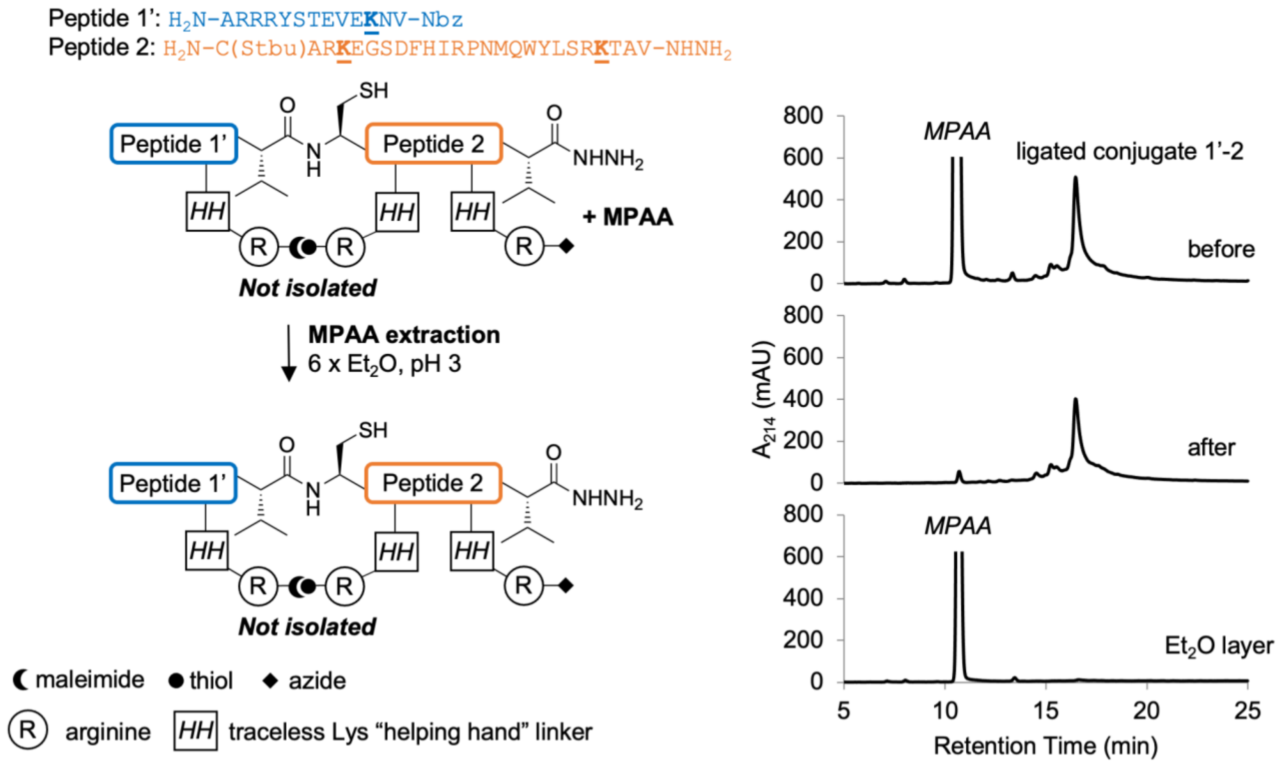

Figure S27. MPAA extraction with Et_2O for CAPTN. After the peptide-Nbz ligation (Fig S26), MPAA removal was achieved with 6 Et_2O extractions at pH 3 (see method section for more detail). The Et_2O layers were combined, the Et_2O was evaporated, and the remaining content was dissolved in RP-HPLC buffer to assess peptide loss. No loss of ligated conjugate 1’-2 (**4’** in main text) was observed. Analytical RP-HPLC method B was used for the analysis. Underlined and bolded Lys (**K**) indicate placements of HH linkers. Nle was substituted for Met in peptide 2.

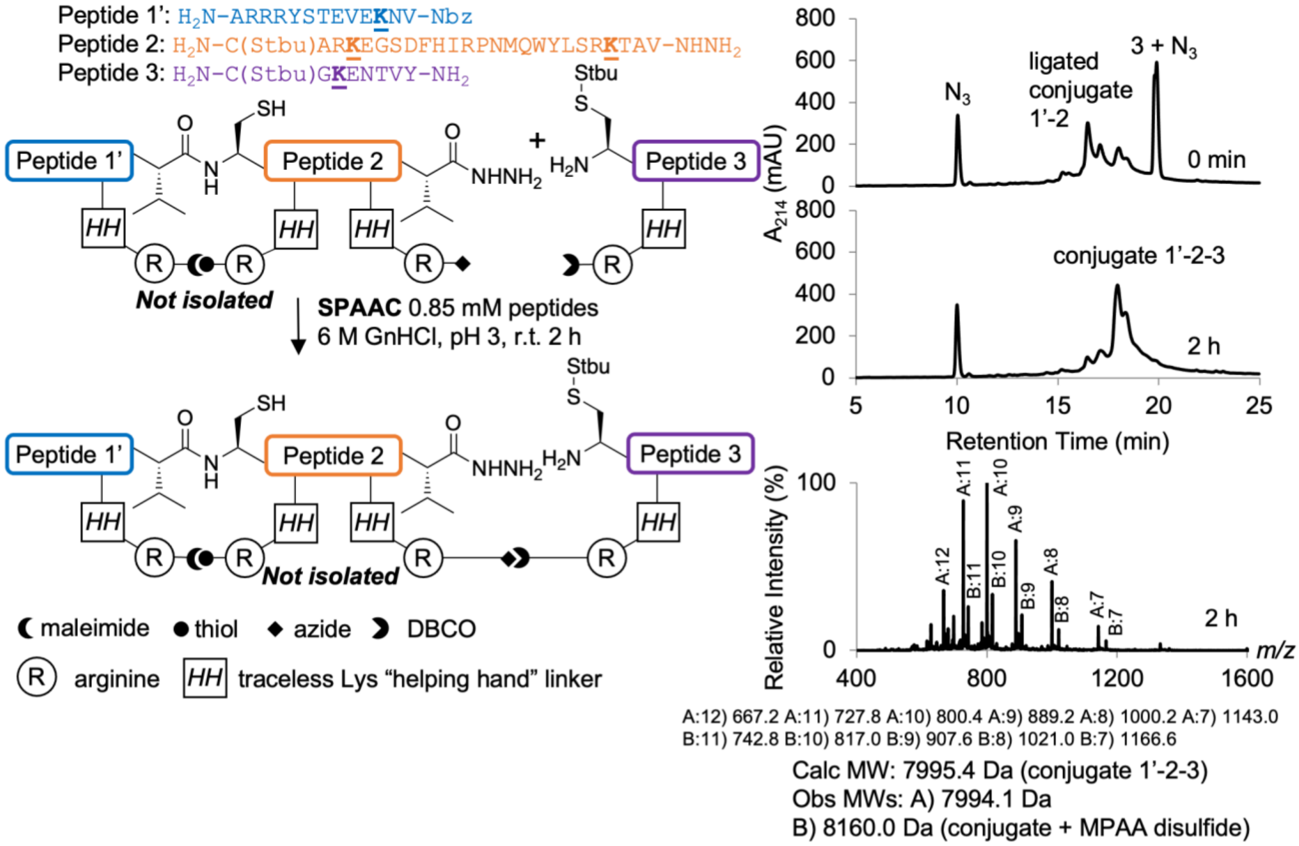

Figure S28. SPAAC reaction for CAPTN. Ligated conjugate 1’-2 (**4’** in main text, Fig S27) was clicked to pure peptide 3 (Fig S6C) by SPAAC. Reaction progression was monitored by analytical RP-HPLC and LC-MS. The time points were quenched with excess 6-

azidohexanoic acid (N_3) to consume unreacted peptide 3-DBCO HH linker. After 2 h, the major product is the expected conjugate 1'-2-3 (**5'** in main text). Analytical RP-HPLC method B and LC-MS method B were used for the analyses. MS from the entire LC-MS chromatogram is reported. Underlined and bolded Lys (**K**) indicate placements of HH linkers. Nle was substituted for Met in peptide 2.

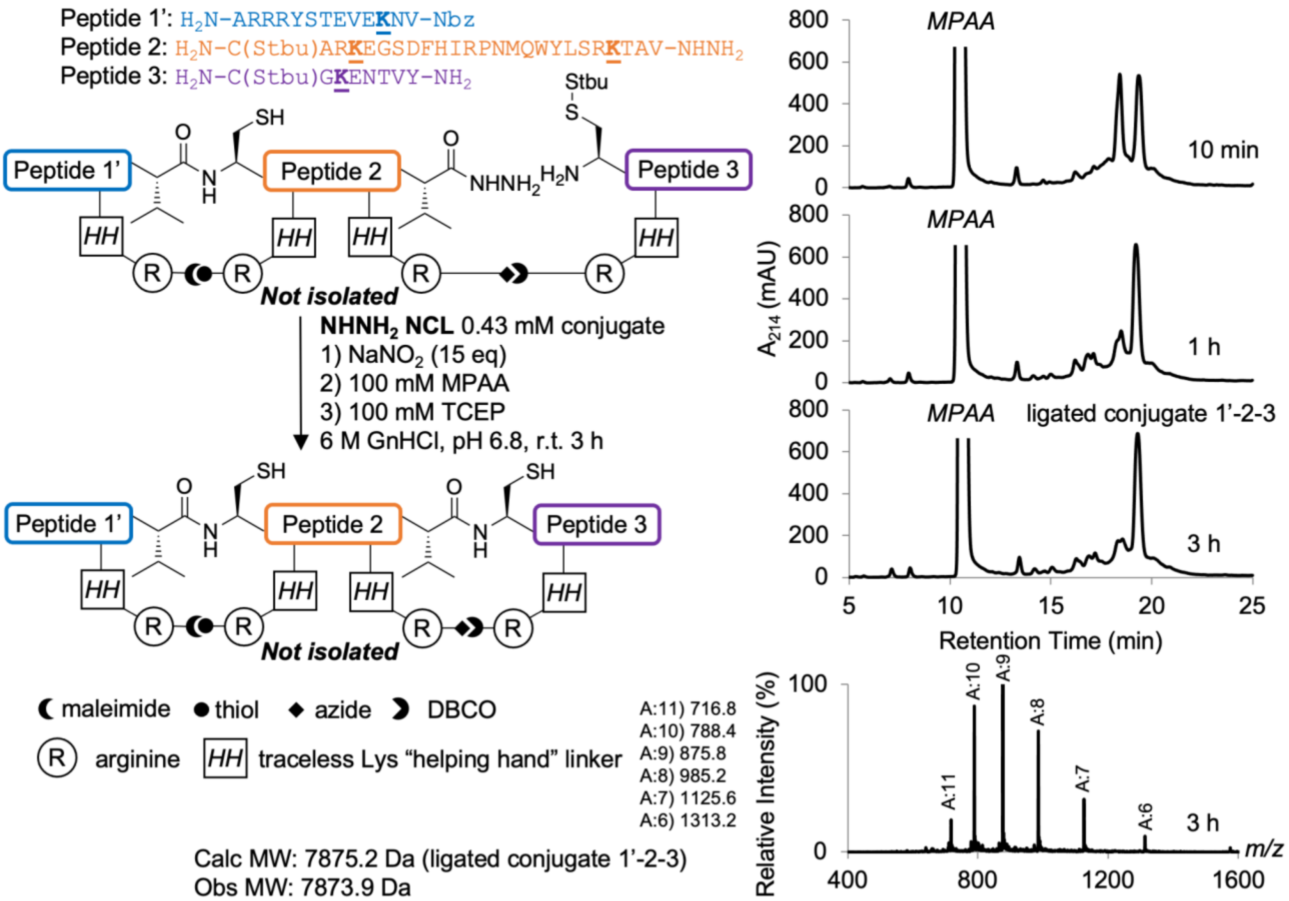

Figure S29. Peptide-$NHNH_2$ ligation for CAPTN. Peptide 2-$NHNH_2$ and peptide 3 from the conjugate 1'-2-3 (**5'** in main text, Fig S28) were ligated in one-pot following SPAAC by treatment with $NaNO_2$ at pH 3 to convert peptide 2-$NHNH_2$ to a thioester via MPAA thiolysis. The reaction was initiated by raising the pH to 6.8. TCEP was then added to remove the Stbu group on peptide 3's N-terminal Cys. Time points of the ligation were taken over the course of 3 h and analyzed by analytical RP-HPLC and LC-MS. Analytical RP-HPLC method B and LC-MS method B were used for the analyses. MS from the entire LC-MS chromatogram is reported. Underlined and bolded Lys (**K**) indicate placements of HH linkers. Nle was substituted for Met in peptide 2.

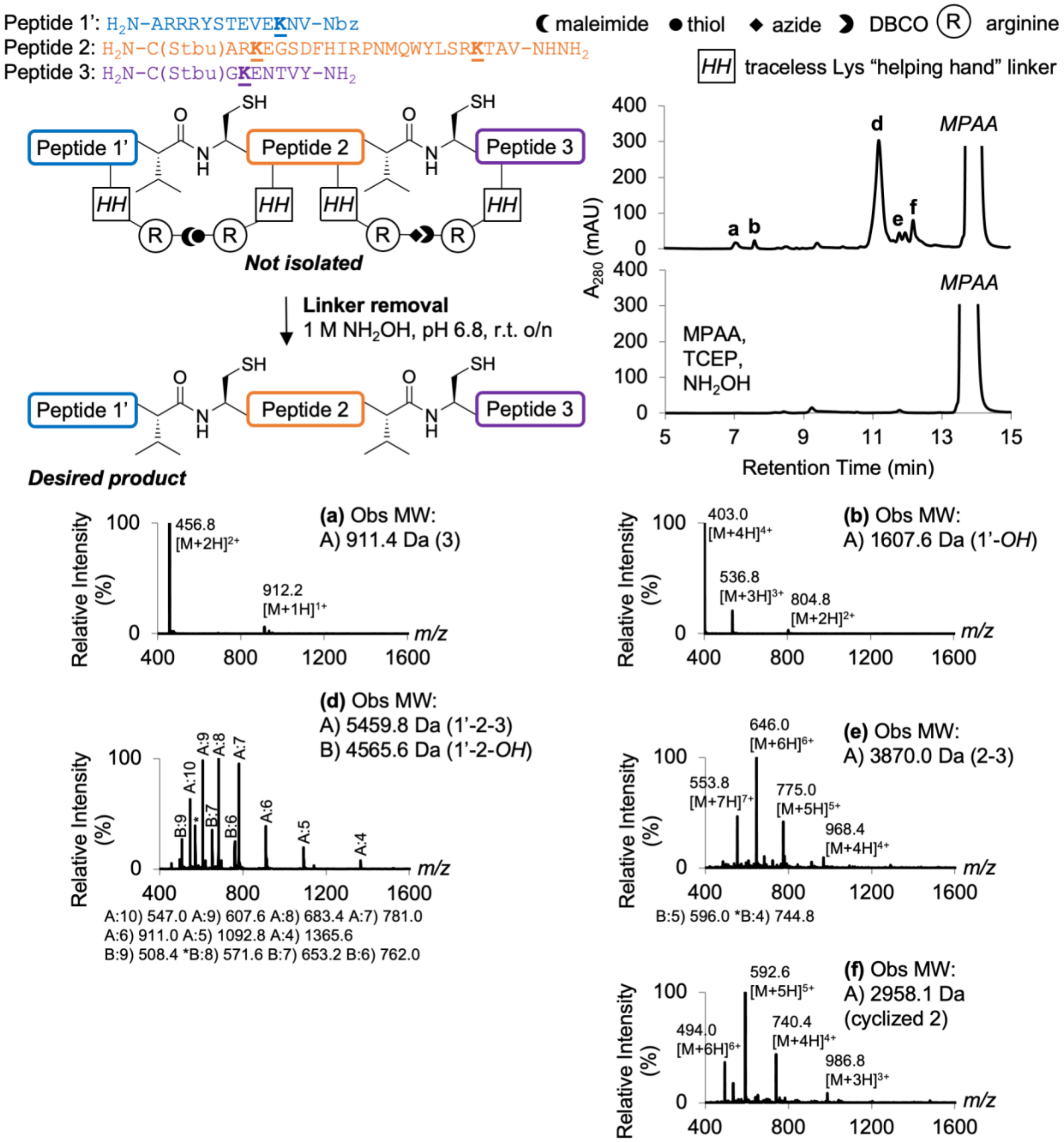

Figure S30. Linker removal following the peptide-NHNH$_2$ NCL from CAPTN. After the peptide-NHNH$_2$ ligation, the ligated conjugate 1'-2-3 (ligated **5'** in main text, Fig S29) was treated with 1 M NH$_2$OH to cleave the HH linkers in one-pot following NCL completion. LC-MS analysis revealed that the main peak d is the desired ligated peptide 1'-2-3 (**6** in main text, 87 % RP-HPLC yield). Cyclization of peptide 2 (peak f, **8** in main text, 6.6 % RP-HPLC yield) was also observed due to incomplete ligation during the peptide-Nbz NCL and negligible cross-ligation between peptides 1' and 3 was observed resulting in fewer side products than in the simultaneous 3-segment templated NCL (Fig S9). The LC chromatogram is also shown in Fig 3. LC-MS method C was used for the analysis. MS from the individually labeled peaks from the LC-MS chromatogram are reported. Underlined and bolded Lys (**K**) indicate placements of HH linkers. Nle was substituted for Met in peptide 2. Italicized *-OH* indicates thioester hydrolysis.

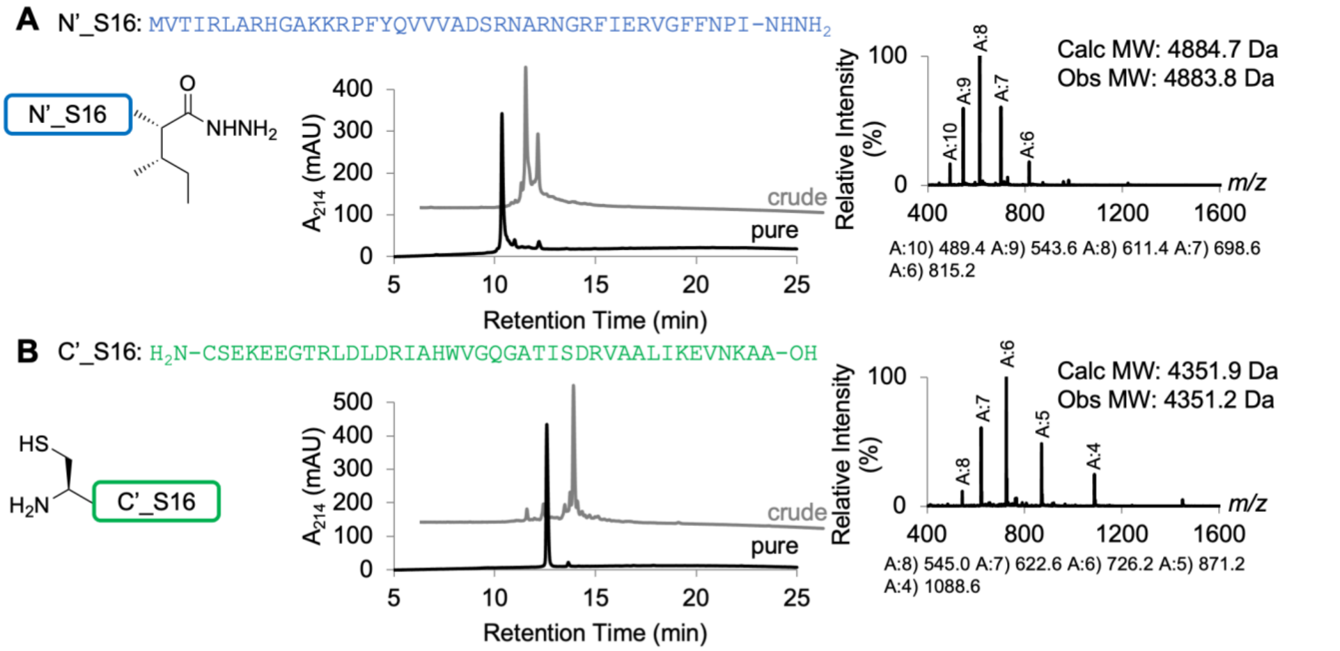

Figure S31. Peptides for the intermolecular NCL of S16. (A) Peptide N'_S16-NHNH2 (**9** in main text) was synthesized at a 50 µmol scale, cleaved under standard 50 µmol scale conditions, and purified using purification method X with an isolated yield of 29% (70.8 mg). Purity of peptide N'_S16-NHNH2 was assessed by analytical RP-HPLC and LC-MS analysis. (B) C'_S16 (**10** in main text) was synthesized at a 50 µmol scale, cleaved under 50 µmol scale conditions with EDT, and purified using purification method Y with an isolated yield of 15% (32.6 mg). Purity of peptide C'_S16 was assessed by analytical RP-HPLC and LC-MS analysis. Analytical RP-HPLC method L and LC-MS method A were used for the analyses of pure and crude peptides. MS of the pure peptides taken from the entire LC-MS chromatograms are reported. Nle was substituted for Met in N'_S16-NHNH2.

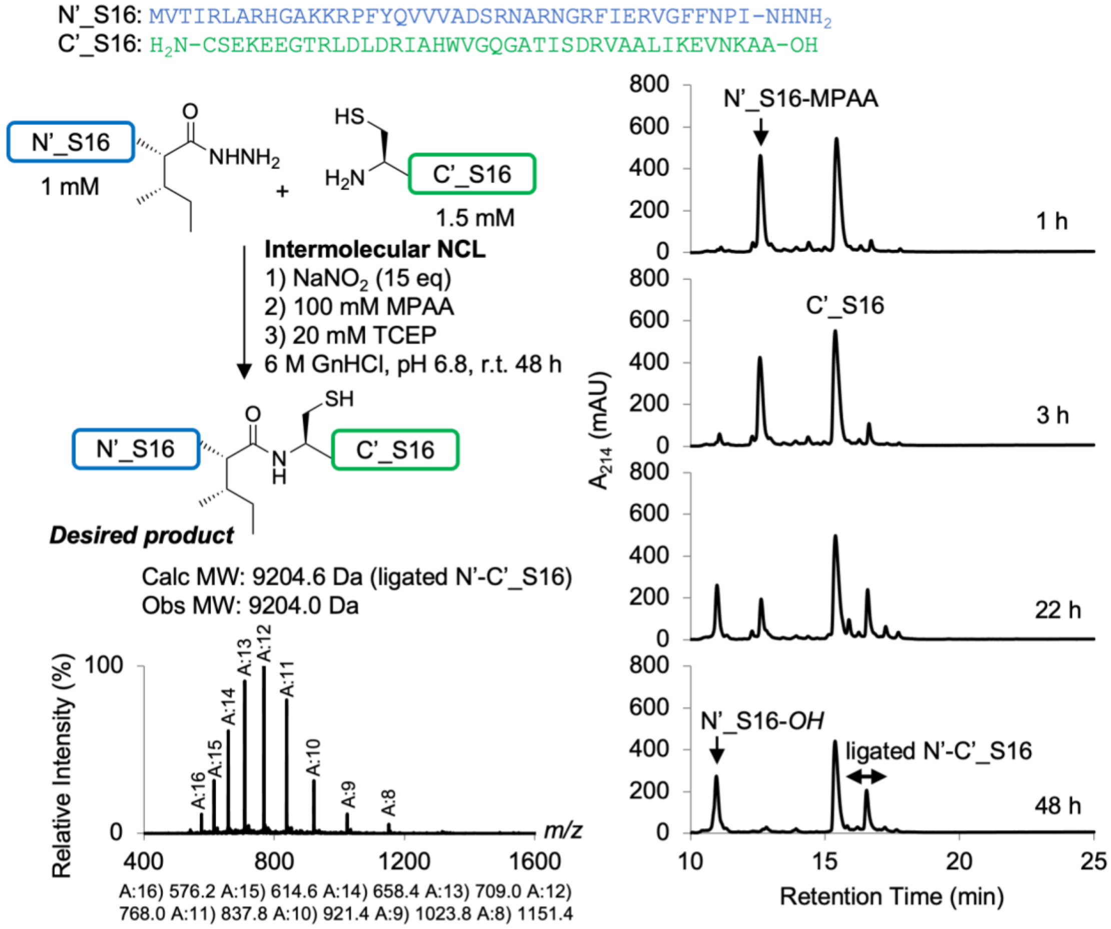

Figure S32. Intermolecular NCL of S16. Peptide N'_S16-NHNH2 (**9** in main text, Fig S31A) was ligated to C'_S16 (**10** in main text, Fig S31B) via peptide-NHNH2 activation by NaNO2 treatment at pH 3 and MPAA thiolysis. The reaction was initiated by adjusting the pH

to 6.8 after which TCEP was added. Depletion of the N'_S16-MPAA thioester (**9a** in main text) was observed at 48 h due to significant hydrolysis (**9b** in main text) resulting in poor conversion to ligated N'-C'_S16 product (**11** in main text, 20 % RP-HPLC yield). The analytical RP-HPLC traces of the 1 h, 22 h, and 48 h time points are also shown in Figure 4A. Analytical RP-HPLC method M and LC-MS method G were used for the analyses. MS of the individually labeled product peak from the analytical RP-HPLC chromatogram is reported. Nle was substituted for Met in N'_S16-NHNH$_2$. Italicized -*OH* indicates thioester hydrolysis.

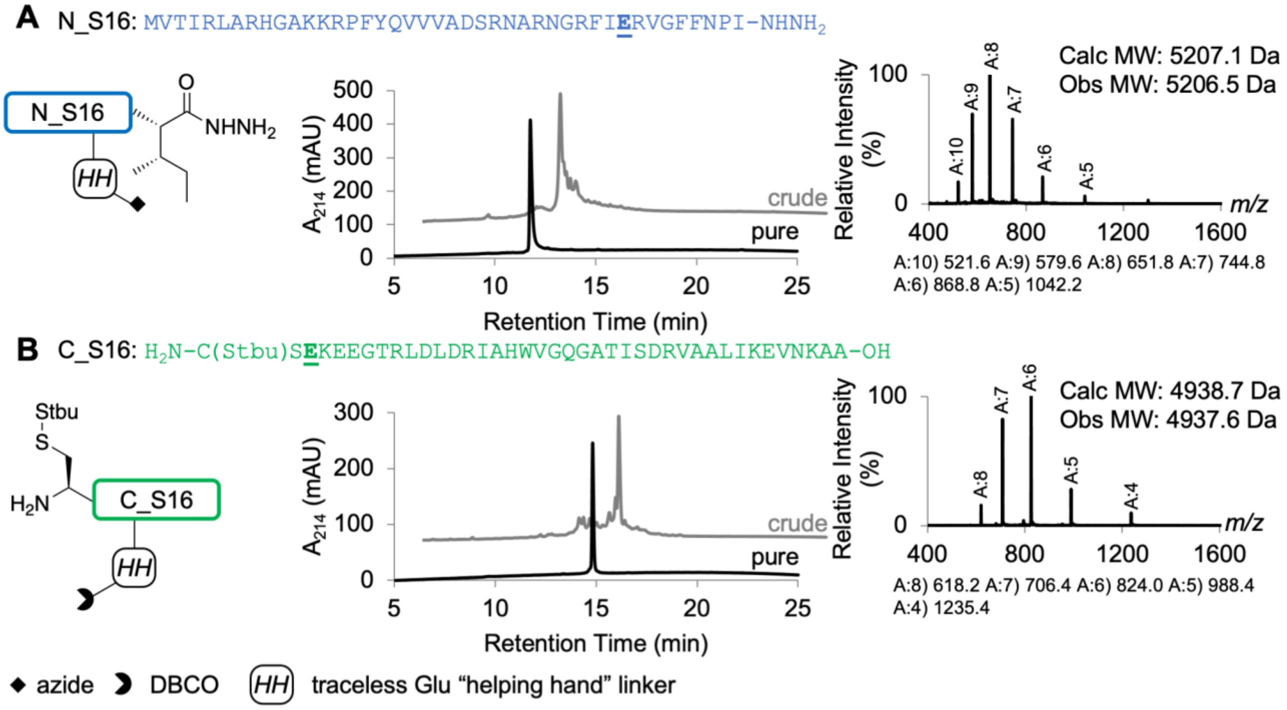

Figure S33. Peptides for the AlHx-mediated CAN of S16. (A) Peptide N_S16-NHNH$_2$ (**12** in main text) was functionalized with an azide-containing HH linker. The sequence matches peptide N'_S16-NHNH$_2$ (Fig S31A). This peptide was synthesized at a 50 µmol scale, cleaved under 50 µmol scale conditions, and purified using purification method Z with an isolated yield of 23% (59.9 mg). Purity of peptide N_S16-NHNH$_2$ was assessed by analytical RP-HPLC and LC-MS analysis. (B) Peptide C_S16 (**13** in main text) was functionalized with a DBCO-containing HH linker. The sequence matches peptide C'_S16 (Fig S31B). This peptide was synthesized at a 50 µmol scale, cleaved under 50 µmol scale conditions with Cu protection, and purified using purification method AA with an isolated yield of 12% (29.6 mg). Purity of peptide C_S16 was assessed by analytical RP-HPLC and LC-MS analysis. Analytical RP-HPLC method L and LC-MS method A were used for the analyses of pure and crude peptides. MS of the pure peptides taken from the entire LC-MS chromatograms are reported. Underlined and bolded Glu (**E**) indicate placements of HH linkers. Nle was substituted for Met in N_S16-NHNH$_2$.

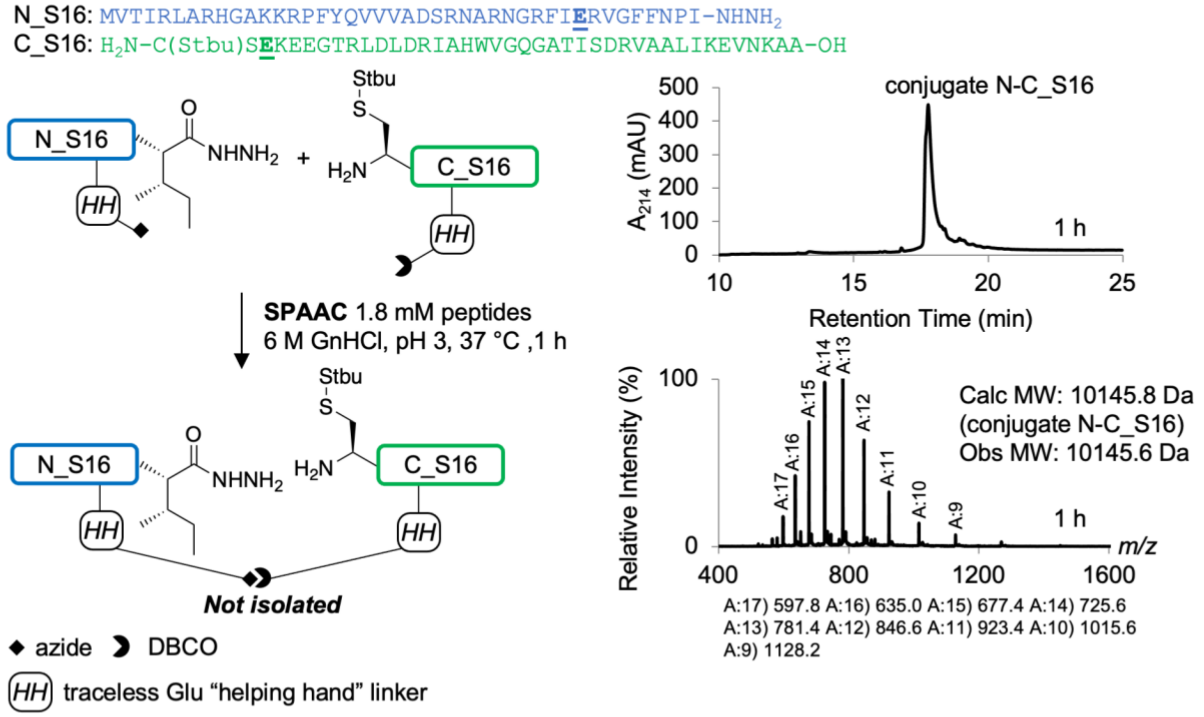

Figure S34. SPAAC reaction for the AlHx-mediated CAN of S16. Pure peptide N_S16-NHNH2 (**12** in main text, Fig S33A) was clicked to pure peptide C_S16 (**13** in main text, Fig S33B). Clean conversion to product conjugates N-C_S16 was observed at 1h. Two peaks are expected due to the formation of regioisomers.[5] The reaction was monitored by analytical RP-HPLC using method M and LC-MS using method G. MS from the entire LC-MS chromatogram is reported. Underlined and bolded Glu (**E**) indicate placements of HH linkers. Nle was substituted for Met in N_S16-NHNH2.

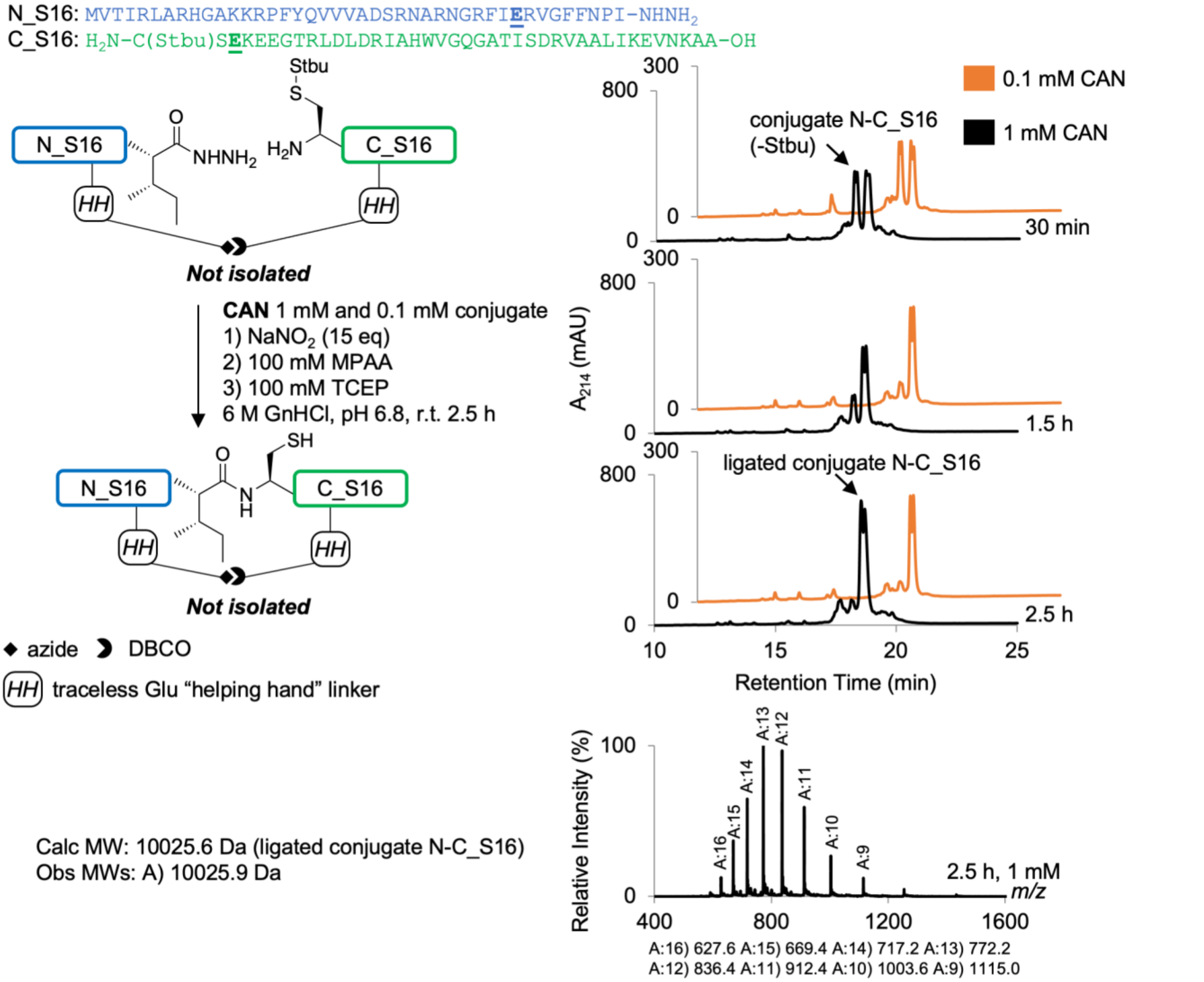

Figure S35. AlHx-mediated CAN of S16. Peptide conjugate N-C_S16 (Fig S34) was ligated via peptide-NHNH2 activation in one-pot following conjugation by treatment with $NaNO_2$ at pH 3 to convert peptide N_S16-NHNH2 to the thioester via MPAA thiolysis. The reaction was initiated by raising the pH to 6.8. TCEP was then added to remove Stbu on peptide C_S16's N-terminal Cys. Two peaks are expected due to the formation of regioisomers during SPAAC.[5] Conversion to the ligated conjugate N-C_S16 (**14** in main text) was observed at 2.5 h. The CAN reactions were conducted at 1 mM conjugate (black) and 0.1 mM conjugate (orange). Both reactions reached completion at 2.5 h due to the concentration-independent templated ligation. Analytical RP-HPLC method M and LC-MS method G were used for analysis. MS from the entire LC-MS chromatogram is reported. Underlined and bolded Glu (**E**) indicate placements of HH linkers. Nle was substituted for Met in N_S16-NHNH2.

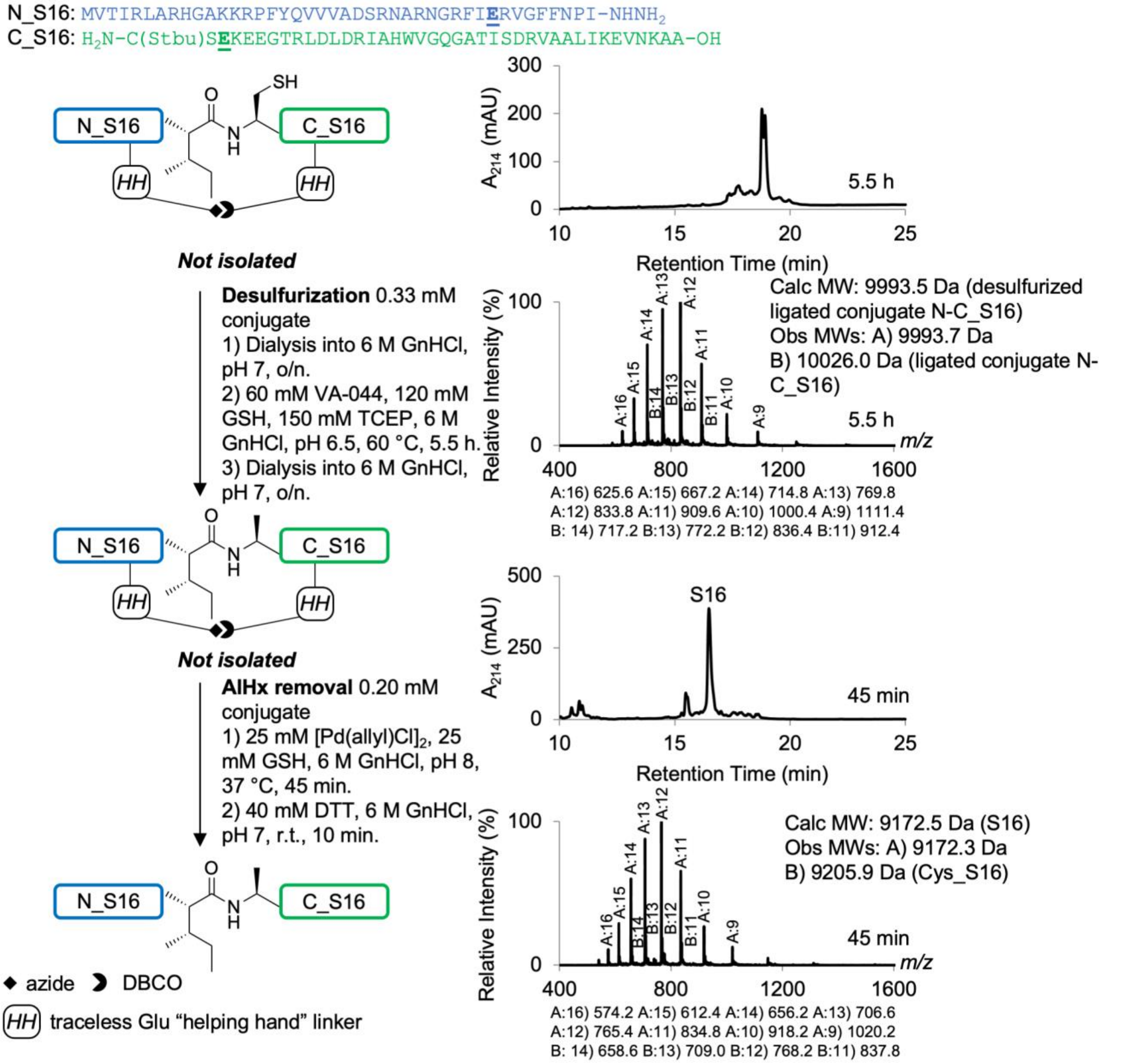

Figure S36. Desulfurization and linker removal following the AlHx-mediated CAN of S16. Ligated conjugate N-C_S16 (**14** in main text, Fig S35) was dialyzed into desulfurization buffer to removed MPAA and desulfurization was done with 60 mM VA-044, 120 mM GSH, and 150 mM TCEP. The reaction reached completion at 5.5 h after which a second dialysis into AlHx removal buffer was done. The AlHx removal was performed with 25 mM $[Pd(allyl)Cl]_2$ and GSH in 45 min. The Pd was quenched with DTT prior to analysis. The analytical RP-HPLC chromatograms and the MS of S16 (**16** in main text) post AlHx removal are also shown in Fig 4B. Analytical RP-HPLC method M and LC-MS method G were used for the analyses. MS of the product peak from the analytical RP-HPLC chromatograms are reported. Underlined and bolded Glu (**E**) indicate placements of HH linkers. Nle was substituted for Met in N_S16-$NHNH_2$.

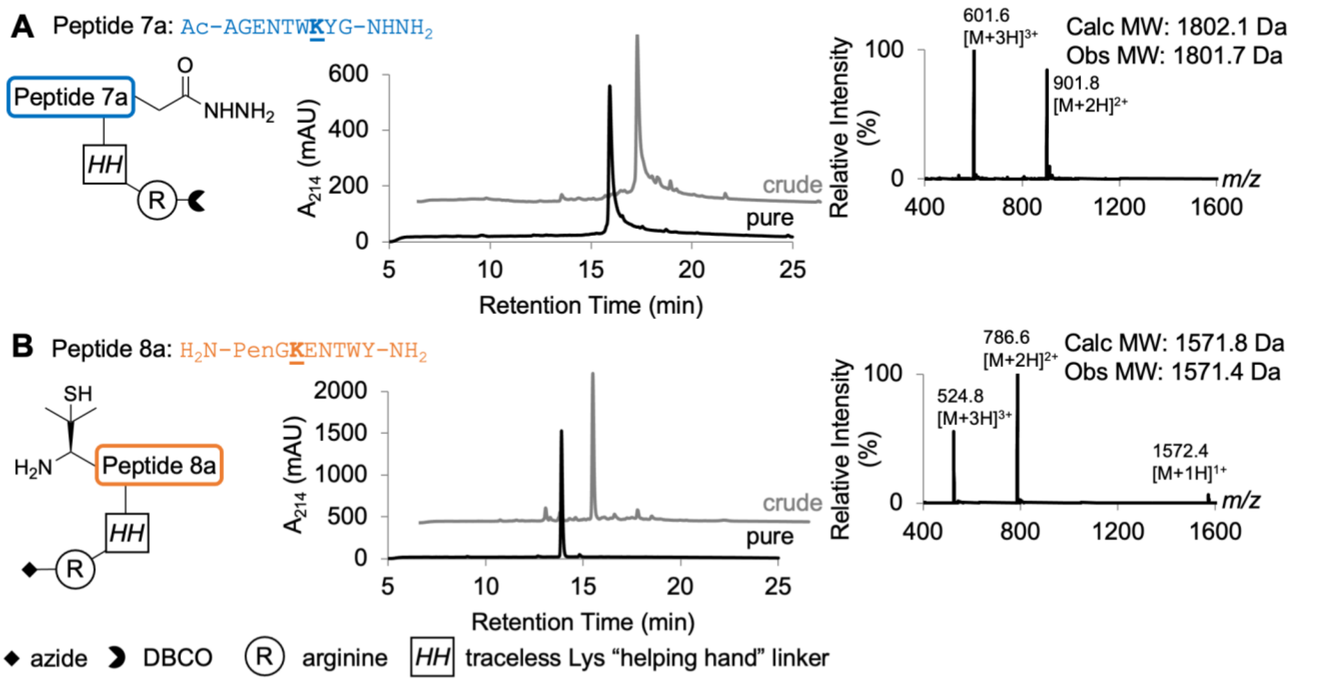

Figure S37. Peptides for CAN at Pen. (A) Peptide 7a-NHNH2 was functionalized with a DBCO-containing HH linker. This peptide was synthesized at a 25 µmol scale, cleaved under 25 µmol scale conditions with Cu protection, and purified using purification method E with an isolated yield of 21% (9.5 mg). Purity of peptide 7a-NHNH2 was assessed by analytical RP-HPLC and LC-MS analysis. (B) Peptide 8a was functionalized with an azide-containing HH linker. This peptide was synthesized at a 25 µmol scale, cleaved under standard 25 µmol scale conditions, and purified using purification method D with an isolated yield of 27% (10.6 mg). Purity of peptide 8a was assessed by analytical RP-HPLC and LC-MS analysis. Analytical RP-HPLC method A and LC-MS method A were used for the analyses of pure and crude peptides. MS of the pure peptides taken from the entire LC-MS chromatograms are reported. Underlined and bolded Lys (**K**) indicate placements of HH linkers.

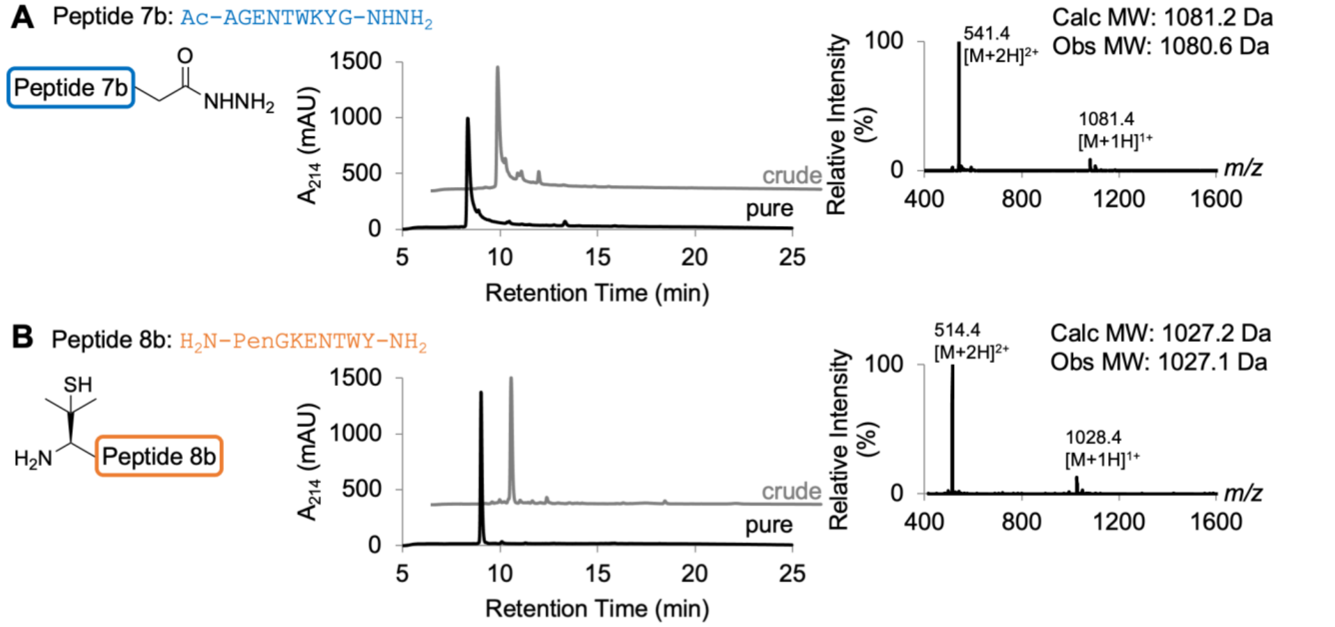

Figure S38. Peptides for the intermolecular NCL at Pen. A) Peptide 7b-NHNH2 was synthesized at a 25 µmol scale, cleaved under standard 25 µmol scale conditions, and purified using purification method L with an isolated yield of 22% (5.9 mg). The sequence matches peptide 7a-NHNH2 (Fig S37A). Purity of peptide 7b-NHNH2 was assessed by analytical RP-HPLC and LC-MS analysis. (B) Peptide 8b was synthesized at a 25 µmol scale, cleaved under 25 µmol scale conditions with EDT, and purified using purification method N with an isolated yield of 29% (7.4 mg). The sequence matches peptide 8a (Fig S37B). Purity of peptide 8b was assessed by analytical RP-HPLC and LC-MS analysis. Analytical RP-HPLC method A and LC-MS method A were used for the analyses of pure and crude peptides. MS of the pure peptides taken from the entire LC-MS chromatograms are reported.

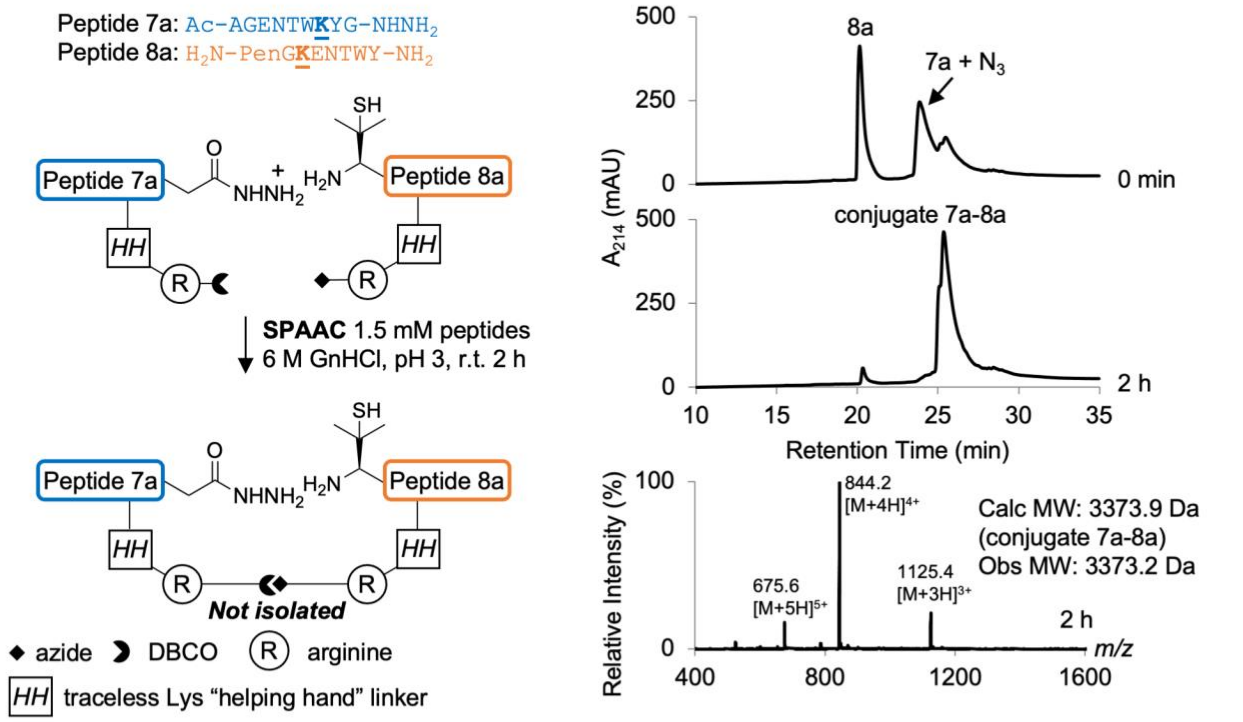

Figure S39. SPAAC reaction for CAN at Pen. Pure peptide 7a-NHNH2 (Fig S37A) was clicked to pure peptide 8a (Fig S37B). The time points were quenched with excess 6-azidohexanoic acid (N_3) to consume unreacted peptide 7a-DBCO HH linker. Clean conversion to product conjugate 7a-8a was observed. Two peaks are expected due to the formation of regioisomers.[5] The reaction was monitored by analytical RP-HPLC using method D and LC-MS using method B. MS from the entire LC-MS chromatogram is reported. Underlined and bolded Lys (**K**) indicate placements of HH linkers.

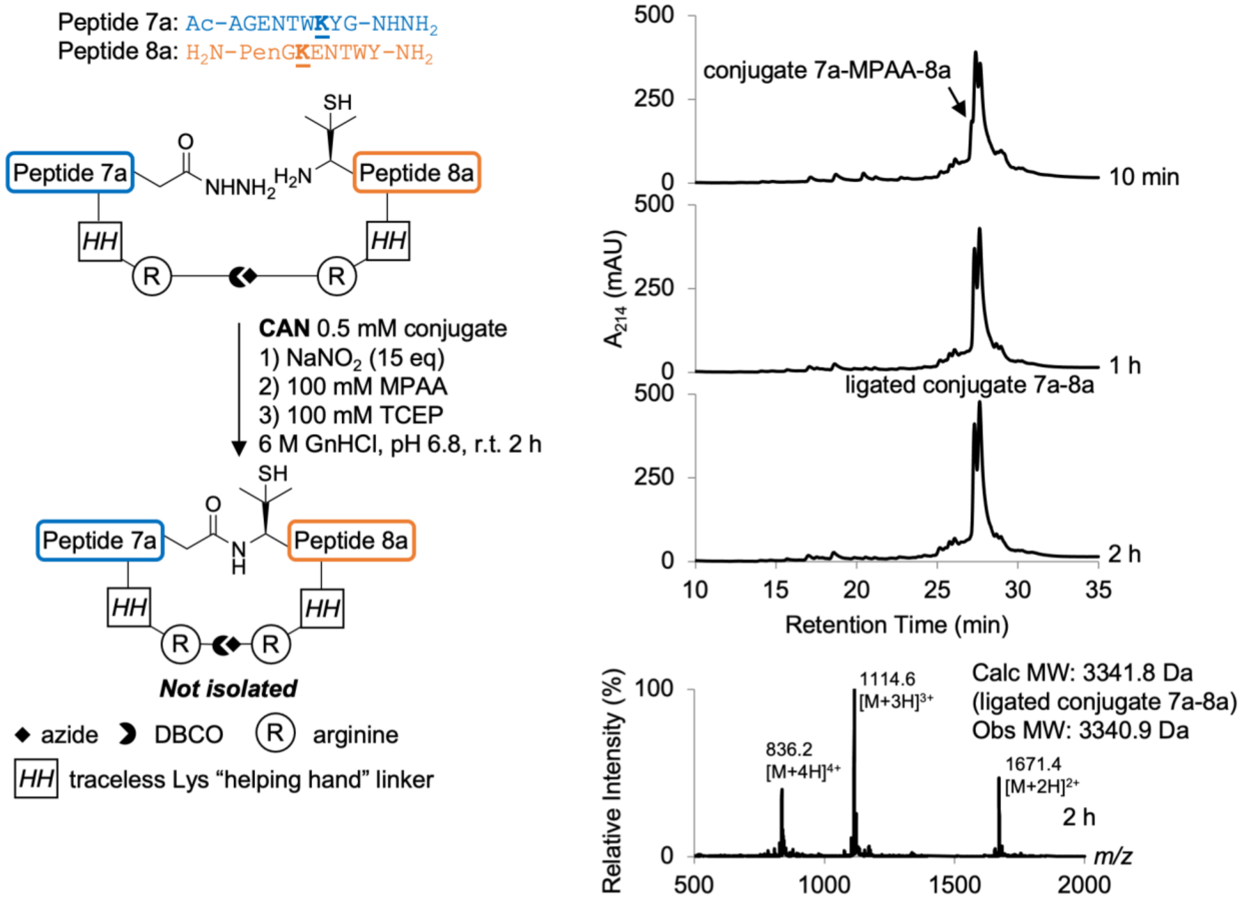

Figure S40. Peptide-NHNH2 CAN at Pen. Peptide conjugate 7a-8a (Fig S39) was ligated via peptide-NHNH2 activation in one-pot following conjugation by treatment with $NaNO_2$ at pH 3 to convert peptide 7a-NHNH2 to the thioester via MPAA thiolysis. The reaction

was initiated by raising the pH to 6.8. Two peaks are expected due to the formation of regioisomers during SPAAC.[5] Conversion to the ligated conjugates 7a-8a was observed at 1 h. Analytical RP-HPLC method D and LC-MS method B were used for analysis. MS from the entire LC-MS chromatogram is reported. Underlined and bolded Lys (**K**) indicate placements of HH linkers.

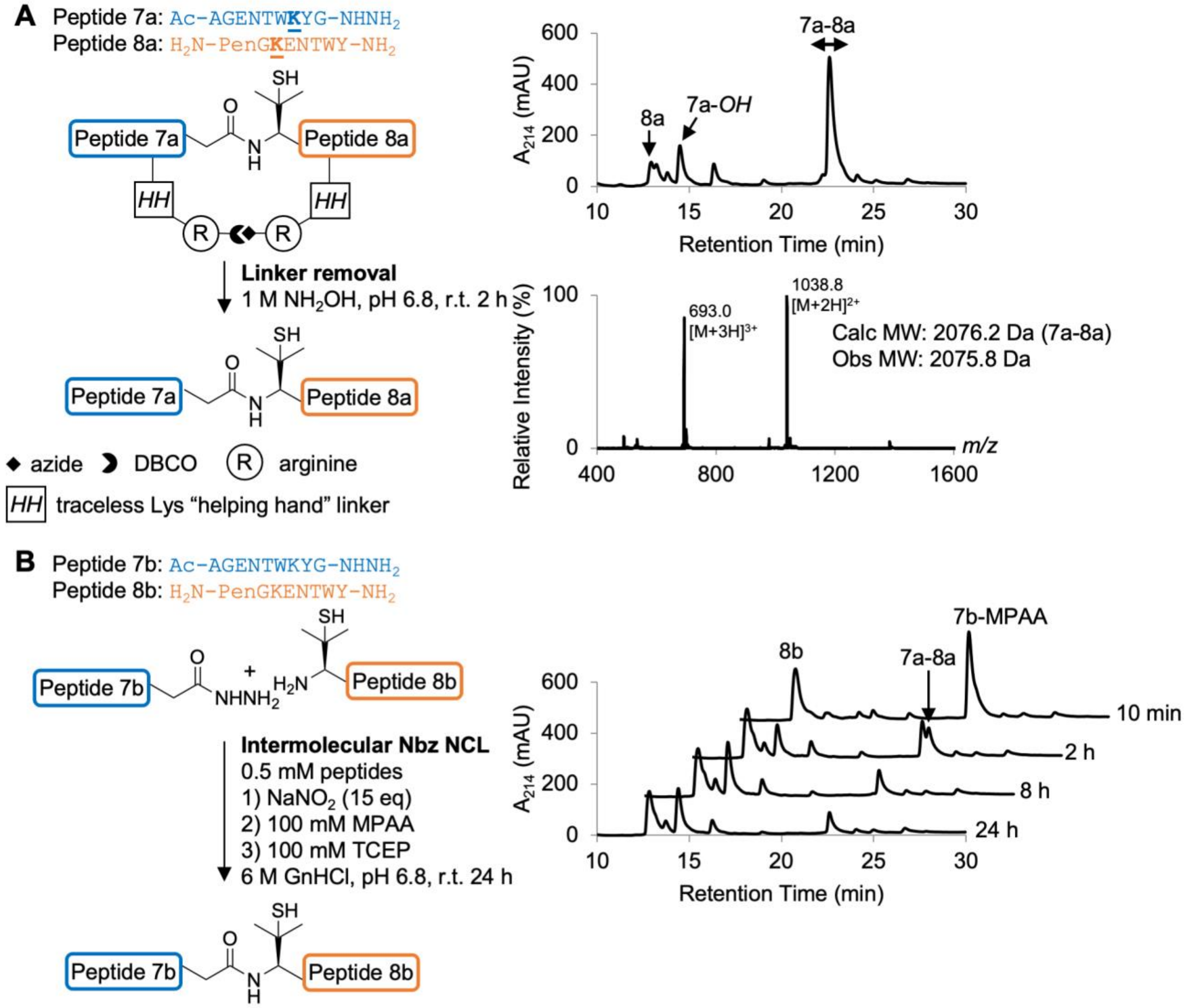

Figure S41. HH linker removal following CAN at Pen and the intermolecular NCL comparison. (A) Linker removal from the CAN at Pen. Ligated conjugate 7a-8a (Fig S40) was treated with 1 M NH_2OH to cleave the HH linkers in one-pot following NCL completion. Analytical RP-HPLC and LC-MS analysis reveal the major product to be the desired ligated 7a-8a. Some unreacted starting material, 7a-*OH* and 8a, are observed due to thioester hydrolysis. (B) Intermolecular NCL control reaction at Pen. Peptide 7d-$NHNH_2$ (Fig S38A) was ligated to peptide 8b (Fig S38B) via peptide-$NHNH_2$ activation by treatment with $NaNO_2$ at pH 3 to convert peptide 7b-$NHNH_2$ to the thioester via MPAA thiolysis. The reaction was initiated by raising the pH to 6.8. TCEP was then added to match the CAN experiment. Depletion of the 7b-MPAA thioester was observed at 24 h due to significant hydrolysis resulting in poor conversion to ligated 7b-8b product. The CAN reaction significantly outperformed the intermolecular reaction at comparable concentrations. Analytical RP-HPLC method H and LC-MS method A were used for the analyses. MS of the individually labeled product peak from the analytical RP-HPLC chromatogram is reported. Underlined and bolded Lys (**K**) indicate placements of HH linkers. Italicized -*OH* indicates thioester hydrolysis.

A S17: H_2N-TDKIRTLQG RVVSDKMEKS IVVAIERFVK HPIYGKFIKR TTKLHVHDEN NECGIGDVVE IRECRPLSKT KSWTLVRVVE KAVL-OH

B S17_T2-L84: H_2N-TDKIRTLQG RVVSDKMEKS IVVAIERFVK HPIYGKFIKR TTKLHVHDEN NECGIGDVVE IRECRPLSKT KSWTLVRVVE KAVL-OH

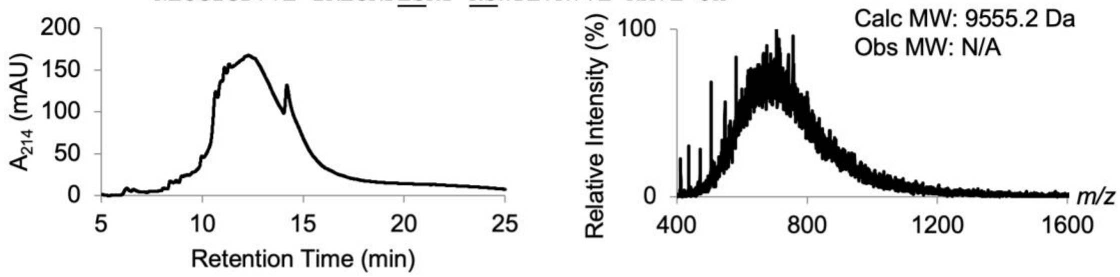

C S17_T2-H45: H_2N-TDKIRTLQG RVVSDKMEKS IVVAIERFVK HPIYGKFIKR TTKLH-NHNH$_2$

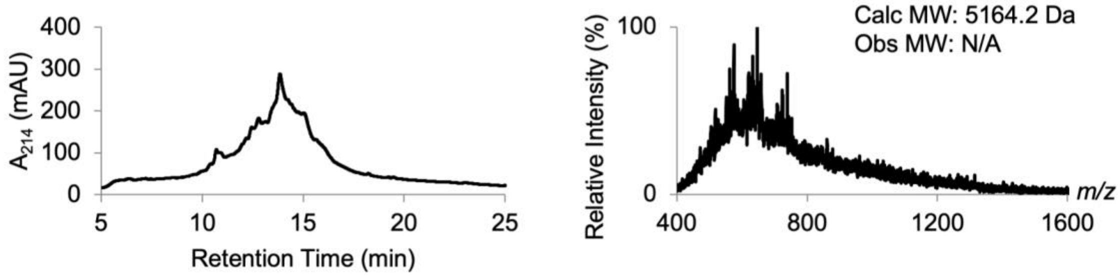

D S17_A24-L84: H_2N-CIERFVK HPIYGKFIKR TTKLHVHDEN NECGIGDVVE IRECRPLSKT KSWTLVRVVE KAVL-OH

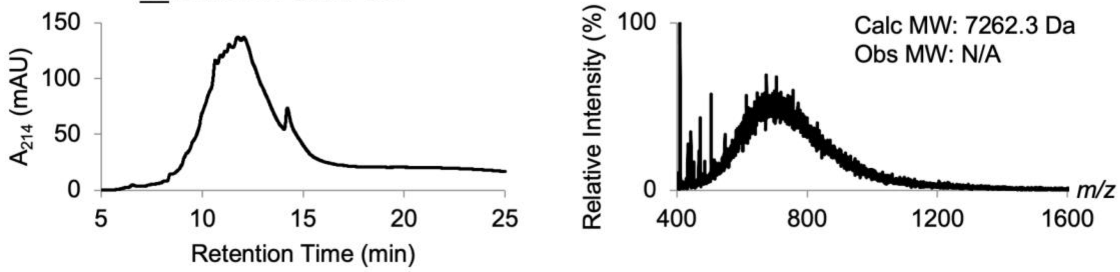

Figure S42. SPPS attempts for the synthesis of S17. (A) Sequence of the *E.coli* ribosomal subunit S17. (B) SPPS of S17_T2-L84. The full-length S17 was synthesized at a 25 µmol scale and cleaved under standard 25 µmol scale conditions. The synthesis was deemed unsuccessful by analytical RP-HPLC and LC-MS analysis. (C) SPPS of S17_T2-H45. The S17 N-terminal half for the two-segment approach with the H45-V46 NCL junction was synthesized at a 25 µmol scale and cleaved under standard 25 µmol scale conditions. The synthesis was deemed unsuccessful by analytical RP-HPLC and LC-MS analysis. (D) SPPS of S17_A24-L84. The S17 C-terminal half for the two-segment approach with the V23-A24 NCL junction was synthesized at a 25 µmol scale and cleaved under 25 µmol scale conditions with EDT. The synthesis was deemed unsuccessful by analytical RP-HPLC and LC-MS analysis. Analytical RP-HPLC method A and LC-MS method B were used for the analyses. MS from the entire LC-MS chromatograms are reported. Italicized residues were double-coupled due to deletions identified in previous attempts of alternative peptides for different approaches (data not shown). Underlined residues indicate use of pseudoprolines to assist in the synthesis. Red M indicate Nle substitutions and red C indicate Acm-protected Cys.

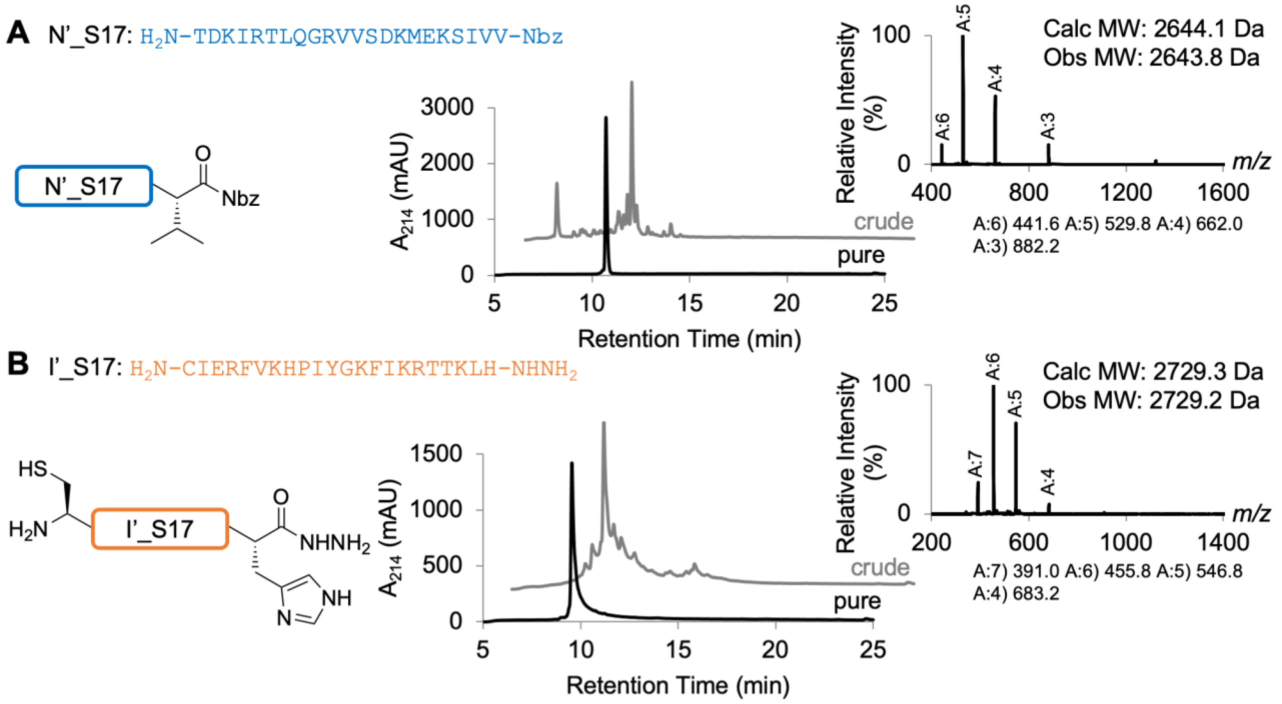

Figure S43. Peptides for the intermolecular NCL of the S17 N-terminal half. (A) Peptide N'_S17-Nbz (**17** in main text) was synthesized at a 25 µmol scale, cleaved under standard 25 µmol scale conditions, and purified using purification method L with an isolated yield of 22% (14.5 mg). Purity of peptide N'_S17-Nbz was assessed by analytical RP-HPLC L and LC-MS analysis. (B) I'_S17-$NHNH_2$ (**18** in main text) was synthesized at a 25 µmol scale, cleaved under 25 µmol scale conditions with EDT, and purified using purification method P with an isolated yield of 18% (12.3 mg). Purity of peptide I'_S17-$NHNH_2$ was assessed by analytical RP-HPLC and LC-MS analysis. Analytical RP-HPLC method A and LC-MS method B were used for the analyses of pure and crude peptides. MS of the pure peptides taken from the entire LC-MS chromatograms are reported. Nle was substituted for Met in N'_S17-Nbz.

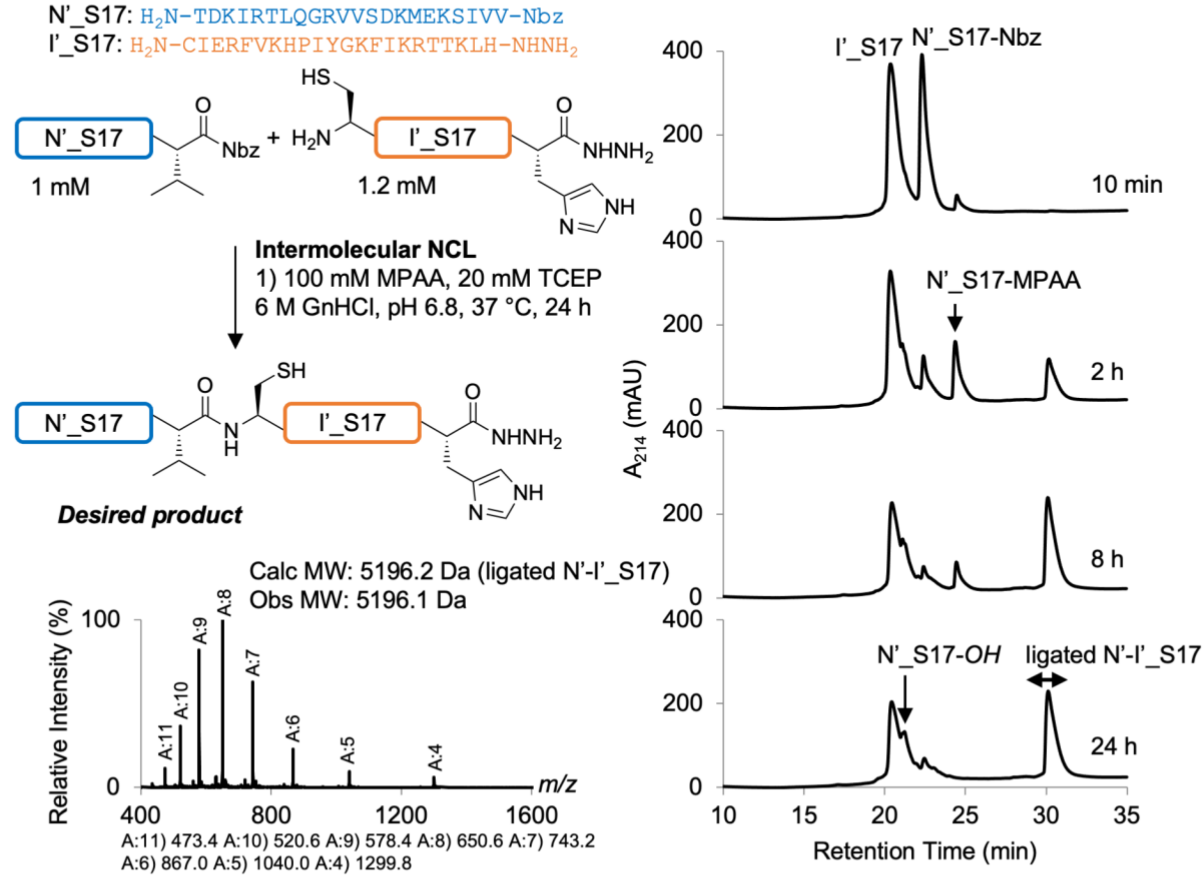

Figure S44. Intermolecular NCL of the S17 N-terminal half. Peptide N'_S17-Nbz (**17** in main text, Fig S43A) was ligated to I'_S17-$NHNH_2$ (**18** in main text, Fig S43B) via peptide-Nbz activation with 100 mM MPAA. The reaction was initiated by adjusting the pH to

6.8 after which TCEP was added. Depletion of the N'_S17-MPAA thioester (**17a** in main text) was observed at 24 h due to hydrolysis (**17b** in main text) resulting in subpar conversion to ligated N'-I'_S17 product (**19** in main text). The analytical RP-HPLC chromatograms are also shown in Fig 5A. Analytical RP-HPLC method I and LC-MS method E were used for the analyses. MS of the individually labeled product peak from the analytical RP-HPLC chromatogram is reported. Nle was substituted for Met in N'_S17-Nbz. Italicized -*OH* indicates thioester hydrolysis.

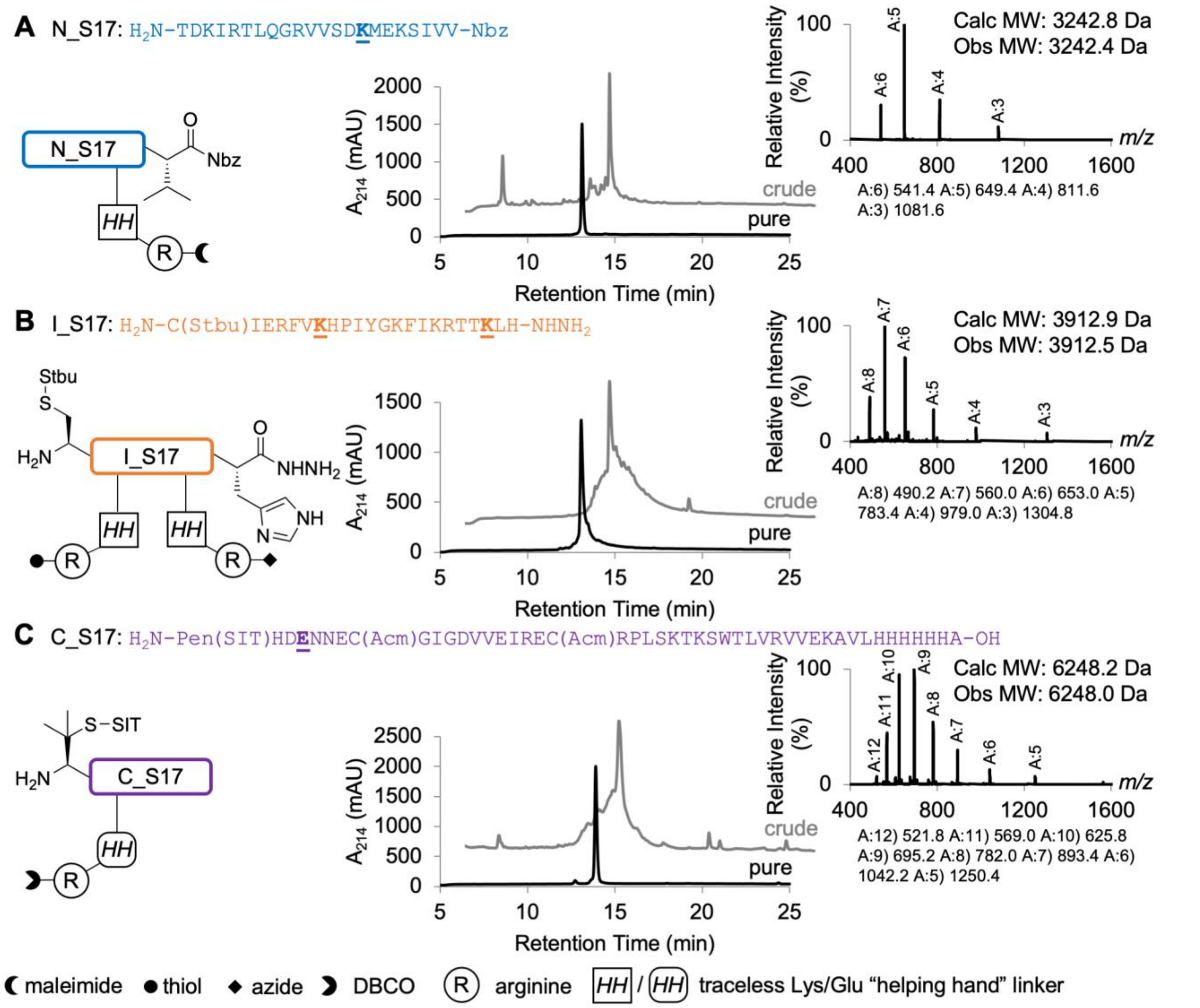

Figure S45. Peptides for the CAPTN synthesis of S17. (A) Peptide N_S17-Nbz (**20** in main text) was functionalized with an maleimide-containing HH linker. The sequence matches peptide N'_S17-Nbz (Fig S43A). This peptide was synthesized at a 50 µmol scale, cleaved under 50 µmol scale conditions, and purified using purification method Q with an isolated yield of 23% (37.3 mg). Purity of peptide N_S17-Nbz was assessed by analytical RP-HPLC and LC-MS analysis. (B) Peptide I_S17-$NHNH_2$ (**21** in main text) was functionalized with a thiol-containing HH linker near its N-terminus and an azide HH linker near its C-terminus. This peptide was synthesized at a 50 µmol scale following steps described in Fig S5, cleaved under standard 50 µmol scale conditions, and purified using purification method R with an isolated yield of 21% (41.1 mg). The sequence matches peptide I'_S17-$NHNH_2$ (Fig S43B). (C) Peptide C_S17 (**22** in main text) was synthesized at a 50 µmol scale with Pen(SIT), functionalized with a DBCO-containing HH linker, cleaved under standard 50 µmol scale conditions with Cu protection, and purified using purification method S with an isolated yield of 9.4% (29.4 mg). Native Cys residues were protected with Acm. Purity of peptide C_S17 was assessed by analytical RP-HPLC and LC-MS analysis. Analytical RP-HPLC method A and LC-MS method B were used for the analyses of pure and crude peptides. MS of the pure peptides taken from the entire LC-MS chromatograms are reported. Underlined and bolded Lys (**K**) and Glu (**E**) indicate placements of HH linkers. Nle was substituted for Met in N_S17-Nbz.

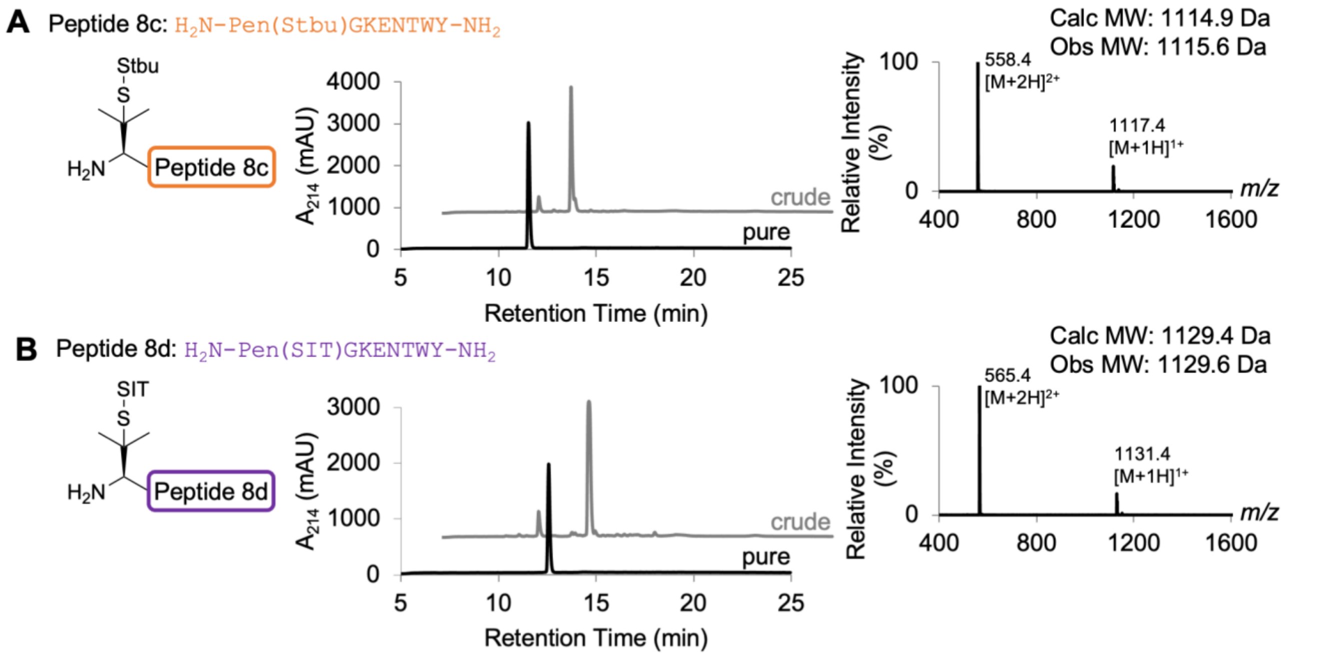

Figure S46. Peptides for assessing Pen protecting groups. (A) Peptide 8c was synthesized at 25 µmol scale with Pen(Stbu), cleaved under standard 25 µmol scale conditions, and purified using purification method T with an isolated yield of 28% (7.8 mg). (B) Peptide 8d was synthesized at 25 µmol scale with Pen(SIT), cleaved under standard 25 µmol scale conditions, and purified using purification method U with an isolated yield of 25% (7.1 mg). Analytical RP-HPLC method A and LC-MS method A were used for the analyses of pure and crude peptides. MS of the pure peptides taken from the entire LC-MS chromatograms are reported.

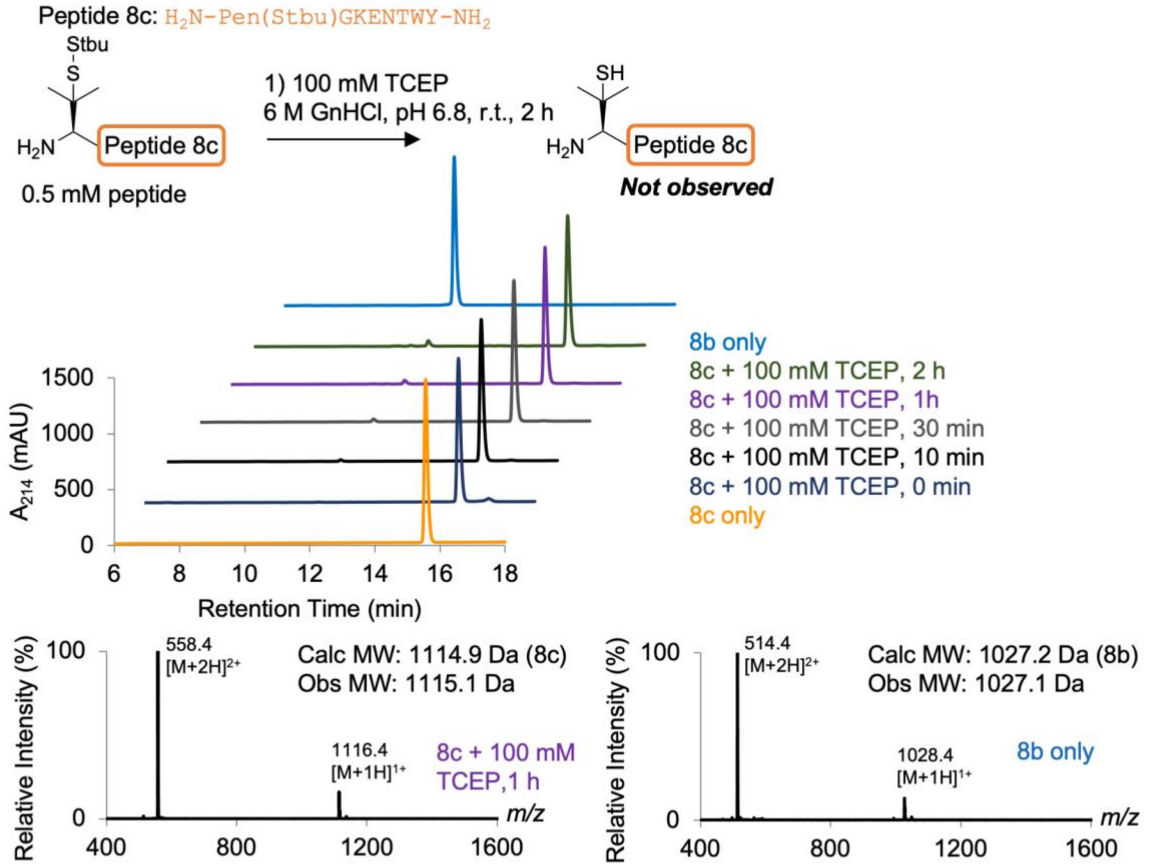

Figure S47. TCEP-mediated Stbu removal on Pen. To assess the TCEP-mediated removal of Stbu on Pen, peptide 8c (Fig S46A) was dissolved in 100 mM TCEP, 6 M GnHCl, at pH 6.8 and the Stbu removal was monitored by analytical RP-HPLC and LC-MS. Minimal Stbu removal was observed over 2 h. Peptide 8b (Fig S38B) has the same sequence as peptide 8c, but with an unprotected Pen, and was used as a reference (light blue trace). Analytical RP-HPLC method G and LC-MS method A were used for the analysis. MS from the entire LC-MS chromatograms are reported.

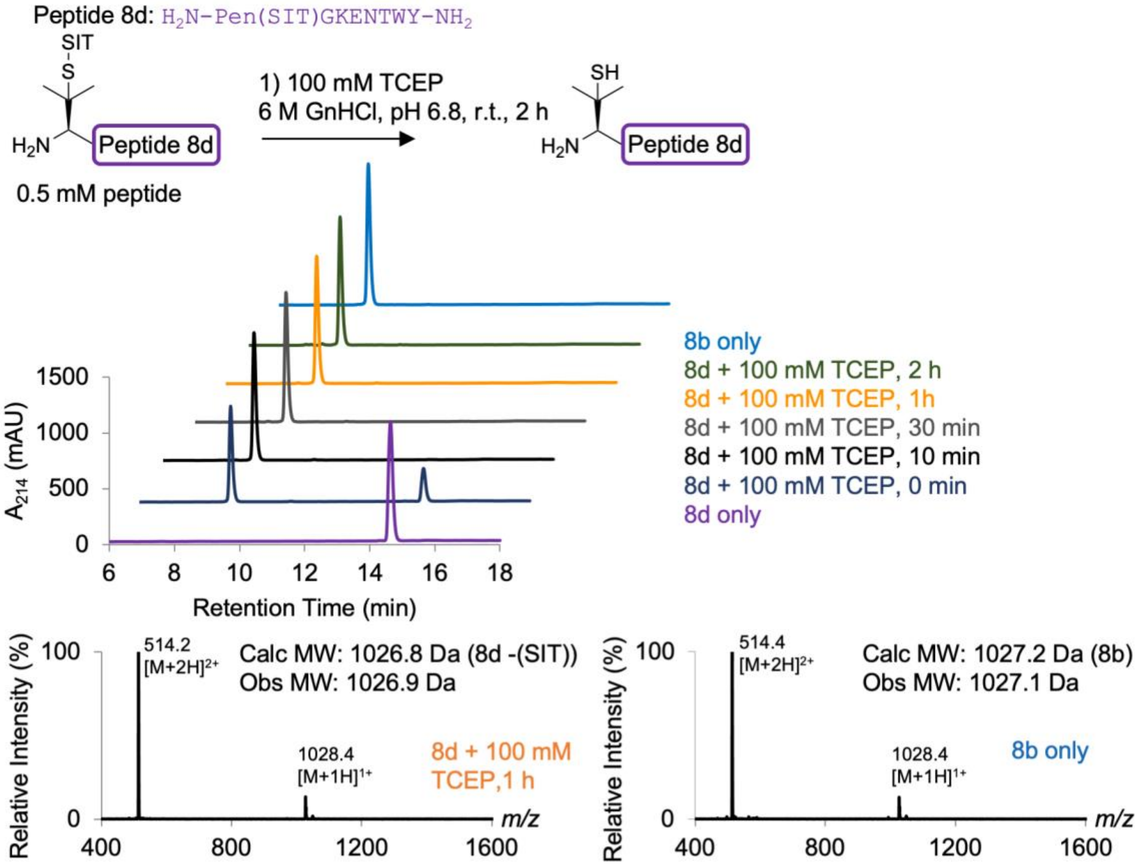

Figure S48. TCEP-mediated SIT removal on Pen. To assess the TCEP-mediated removal of SIT on Pen, peptide 8d (Fig S46b) was dissolved in 100 mM TCEP, 6 M GnHCl, at pH 6.8 and the SIT removal was monitored by analytical RP-HPLC and LC-MS. Complete SIT removal was observed at 10 min. Peptide 8b (Fig S38B) has the same sequence as peptide 8d, but with an unprotected Pen, and was used as a reference (light blue trace). Analytical RP-HPLC method F and LC-MS method A were used for the analysis. MS from the entire LC-MS chromatograms are reported.

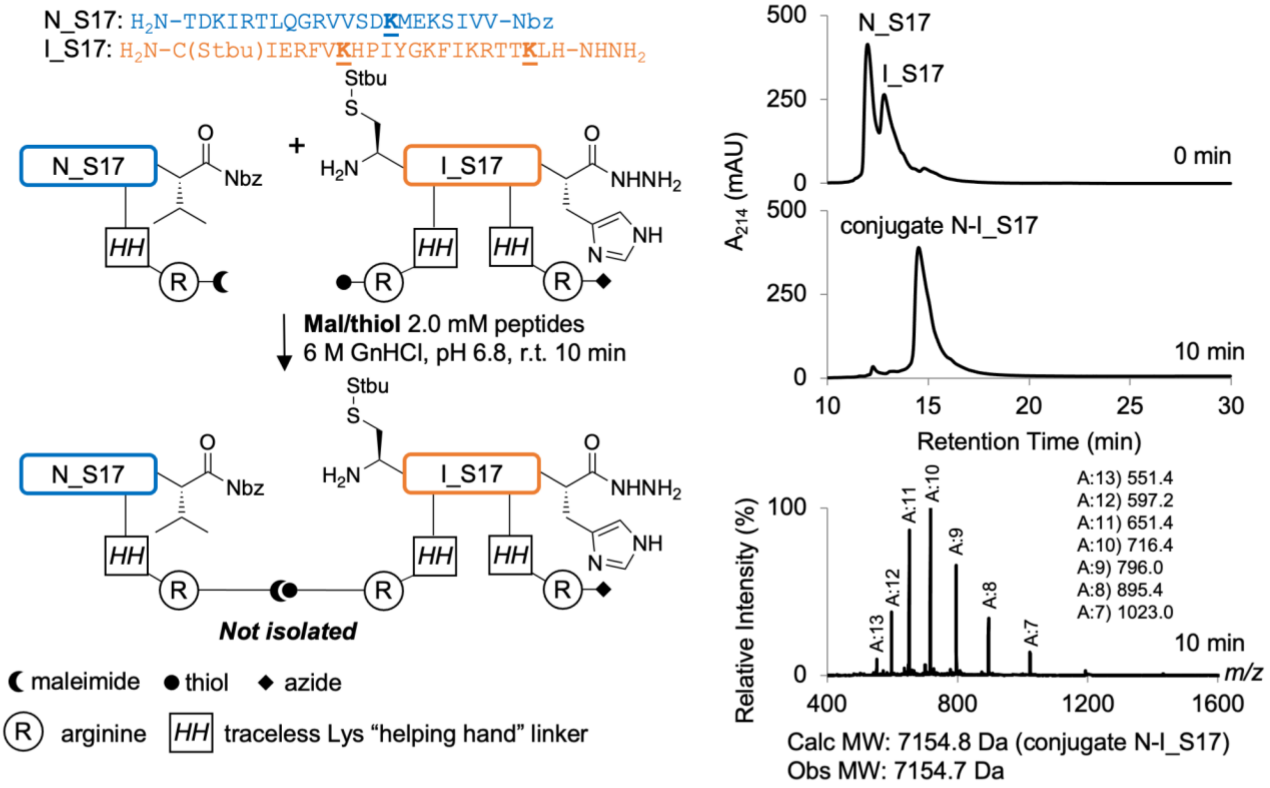

Figure S49. Maleimide/thiol conjugation reaction for the CAPTN synthesis of S17. Pure peptide N_S17-Nbz (**20** in main text, Fig S45A) was conjugated to pure peptide I_S17-NHNH₂ (**21** in main text, Fig S45B). The reaction was monitored by analytical RP-HPLC and LC-MS. Conversion to product conjugate N-I_S17 (**23** in main text) was achieved in 10 min. Note that the 0 min time point was conducted at pH 3 to catch the individual peptides. The pH was adjusted to 6.8 to initiate the conjugation. The analytical RP-HPLC chromatogram of the 10 min time point is also shown in Fig 5B. Analytical RP-HPLC method J and LC-MS method B were used for the analyses. MS from the entire LC-MS chromatogram is reported. Underlined and bolded Lys (**K**) indicate placements of HH linkers. Nle was substituted for Met in N_S17-Nbz.

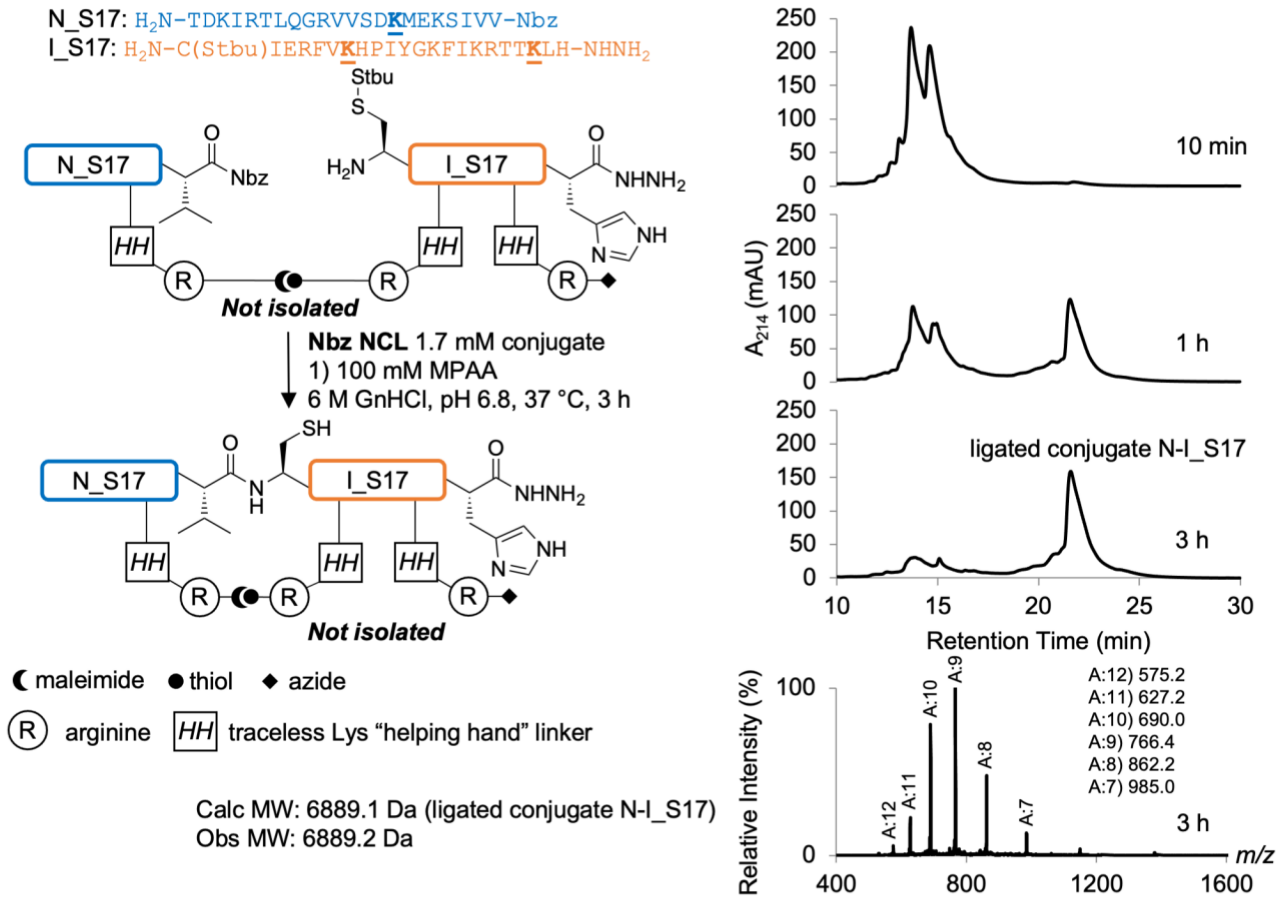

Figure S50. Peptide-Nbz ligation for the CAPTN synthesis of S17. Conjugate N-I_S17 (**23** in main text, Fig S49) was ligated in one-pot following conjugation by treatment with 100 mM MPAA at pH 6.8 to convert peptide N_S17-Nbz to the thioester while maintaining peptide I_S17-NHNH₂ as a peptide-NHNH₂. Time points of the ligation were taken over the course of 3 h and analyzed by analytical RP-HPLC and LC-MS. Conversion to the ligated conjugate N-I_S17 (**24** in main text) was observed at the 3 h time point. The analytical RP-HPLC chromatogram of the 3 h time point is also shown in Fig 5B. Analytical RP-HPLC method J and LC-MS method B were used for the analyses. MS from the entire LC-MS chromatogram is reported. Underlined and bolded Lys (**K**) indicate placements of HH linkers. Nle was substituted for Met in N_S17-Nbz.

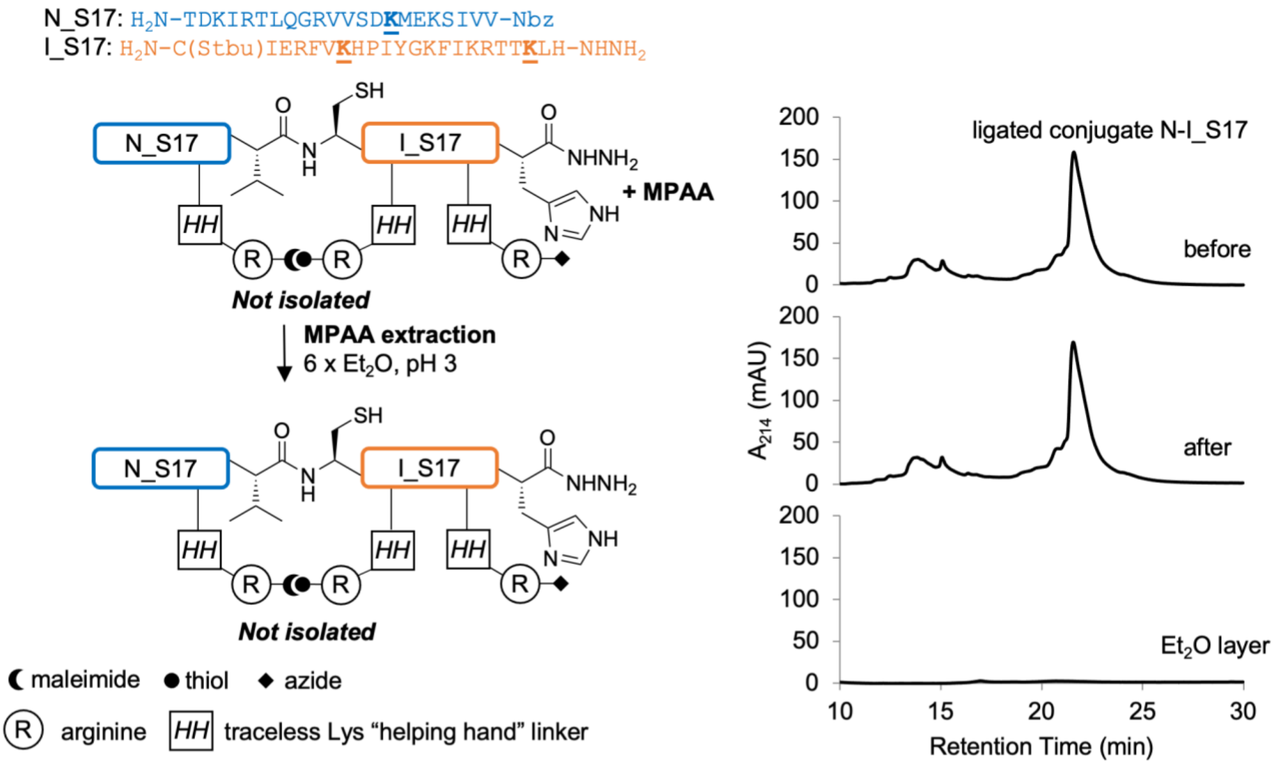

Figure S51. The first MPAA extraction with Et_2O prior to SPAAC for the CAPTN synthesis of S17. After the peptide-Nbz ligation (Fig S50), MPAA removal was achieved with 6 Et_2O extractions at pH 3 (see method section for more detail). The Et_2O layers were combined, the Et_2O was evaporated, and the remaining content was dissolved in RP-HPLC buffer to assess peptide loss. No loss of ligated conjugate N-I_S17 (**24** in main text) was observed. Analytical RP-HPLC method J was used for the analysis. Underlined and bolded Lys (**K**) indicate placements of HH linkers. Nle was substituted for Met in N_S17-Nbz.

N_S17: H_2N-TDKIRTLQGRVVSD**K**MEKSIVV-Nbz
I_S17: H_2N-C(Stbu)IERFV**K**HPIYGKFIKRTT**K**LH-$NHNH_2$
C_S17: H_2N-Pen(SIT)HD**E**NNEC(Acm)GIGDVVEIREC(Acm)RPLSKTKSWTLVRVVEKAVLHHHHHHA-OH

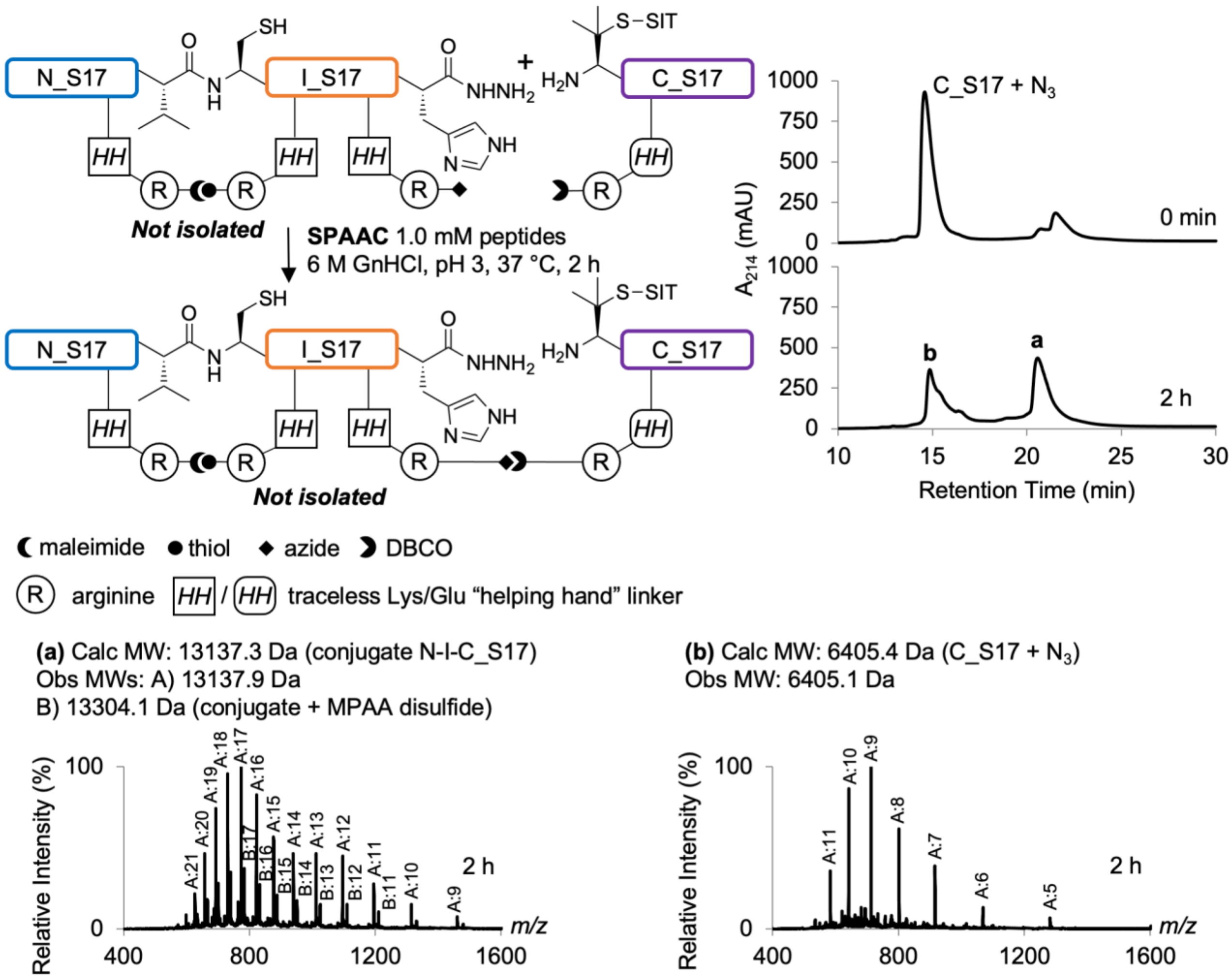

A:21) 626.6 A:20) 658.0 A:19) 692.6 A:18) 731.0 A:17) 773.8 A:16) 822.2 A:15) 876.8 A:14) 939.4 A:13) 1011.6 A:12) 1095.8 A:11) 1195.2 A:10) 1314.6 A:9) 1460.6 B:17) 783.6 B:16) 832.6 B:15) 888.0 B:14) 951.2 B:13) 1024.4 B:12) 1109.6 B:11) 1210.4

A:11) 583.4 A:10) 641.6 A:9) 712.6 A:8) 801.6 A:7) 916.0 A:6) 1068.4 A:5) 1281.8

Figure S52. SPAAC reaction for the CAPTN synthesis of S17. Ligated conjugate N-I_S17 (**24** in main text, Fig S51) was clicked to pure peptide C_S17 (**22** in main text, Fig S45C) by SPAAC. Reaction progression was monitored by analytical RP-HPLC and LC-MS. The time points were quenched with excess 6-azidohexanoic acid (N_3) to consume unreacted peptide C_S17-DBCO HH linker. After 2 h, the major product is the expected conjugate N-I-C_S17 with unreacted C_S17. Analytical RP-HPLC method J and LC-MS method F were used for the analyses. MS of the individually labeled peaks from the analytical RP-HPLC chromatogram are reported. Underlined and bolded Lys (**K**) and Glu (**E**) indicate placements of HH linkers. Nle was substituted for Met in N_S17-Nbz.

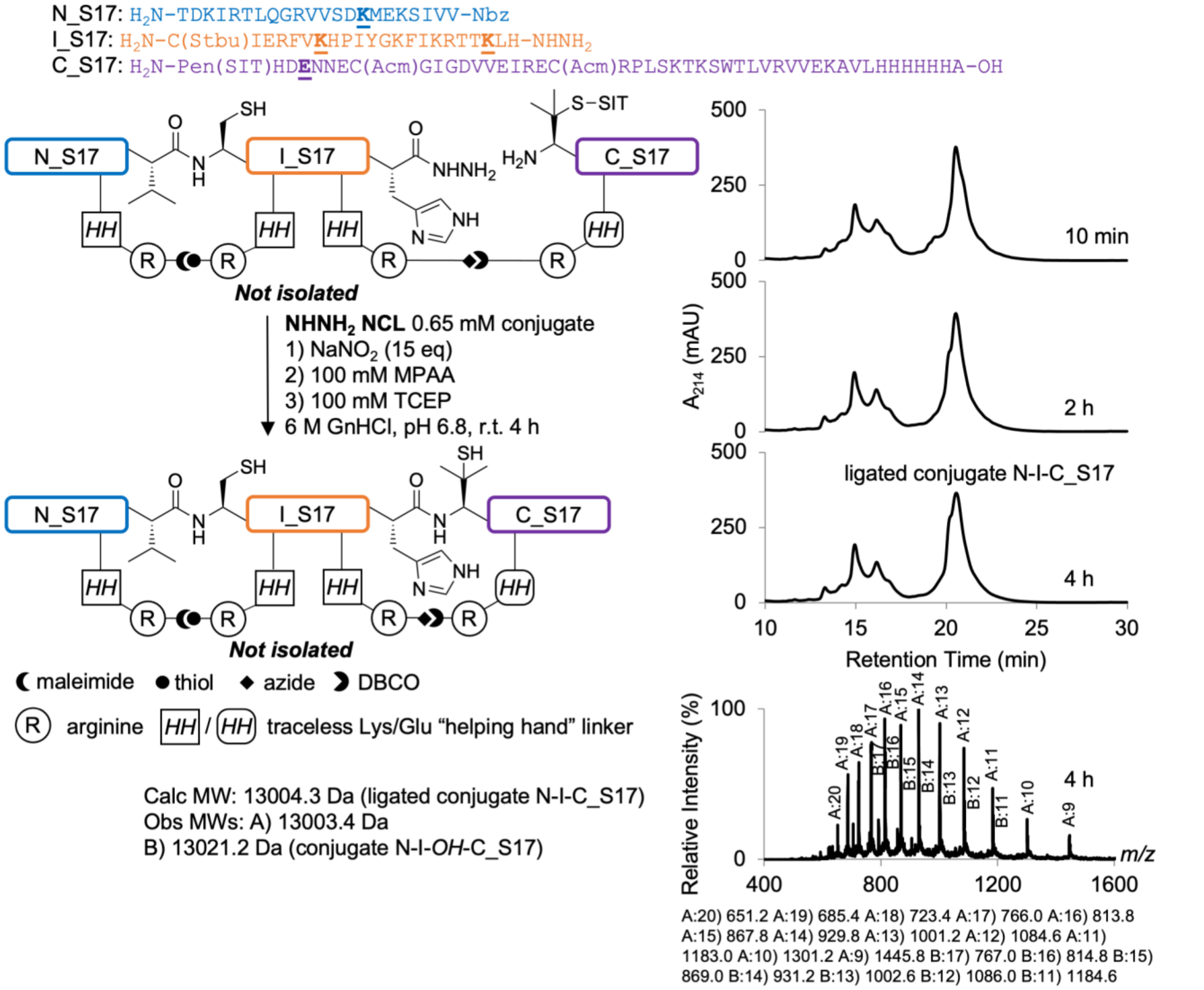

Figure S53. Peptide-NHNH2 ligation for the CAPTN synthesis of S17. Peptide I_S17-NHNH2 and peptide C_S17 from the N-I-C_S17 conjugate (Fig S52) were ligated in one-pot following SPAAC by treatment with $NaNO_2$ at pH 3 to convert peptide I_S17-NHNH2 to a thioester via MPAA thiolysis. The reaction was initiated by raising the pH to 6.8. TCEP was then added to remove the SIT group on peptide C_S17's N-terminal Pen. Time points of the ligation were taken over the course of 4 h and analyzed by analytical RP-HPLC and LC-MS. While the major product is the expected ligated conjugate N-I-C_S17, some hydrolysis of the thioester was observed resulting in the N-I-*OH*-C_S17 hydrolyzed conjugate. Analytical RP-HPLC method J and LC-MS method F were used for the analyses. MS of the individually labeled product peak from the analytical RP-HPLC chromatogram is reported. Underlined and bolded Lys (**K**) and Glu (**E**) indicate placements of HH linkers. Nle was substituted for Met in N_S17-Nbz. Italicized -*OH* indicates thioester hydrolysis.

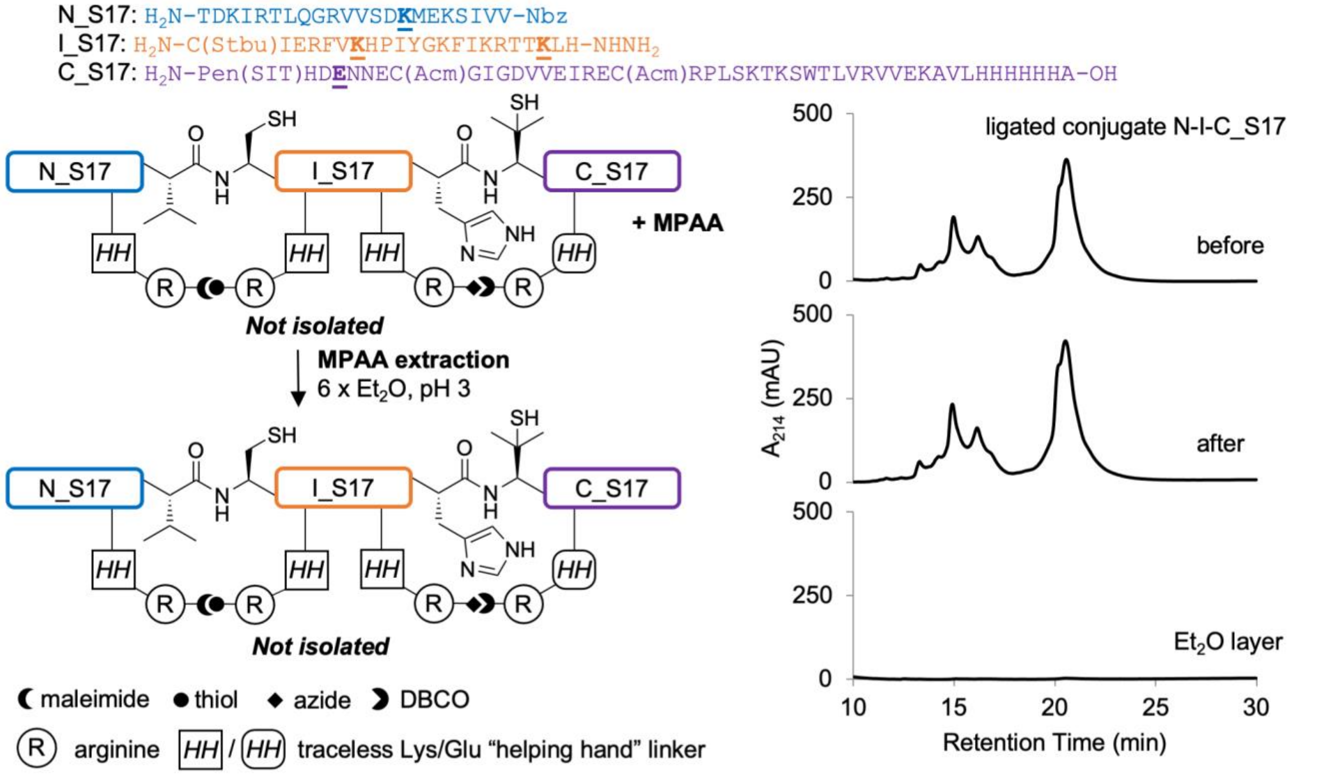

Figure S54. The second MPAA extraction with Et_2O prior to desulfurization for the CAPTN synthesis of S17. After the peptide-$NHNH_2$ ligation (Fig S53), MPAA removal was achieved with 6 Et_2O extractions at pH 3 (see method section for more detail). The Et_2O layers were combined, the Et_2O was evaporated, and the remaining content was dissolved in RP-HPLC buffer to assess peptide loss. No loss of ligated conjugate N-I-C_S17 was observed. Analytical RP-HPLC method J was used for the analysis. Underlined and bolded Lys (**K**) and Glu (**E**) indicate placements of HH linkers. Nle was substituted for Met in N_S17-Nbz.

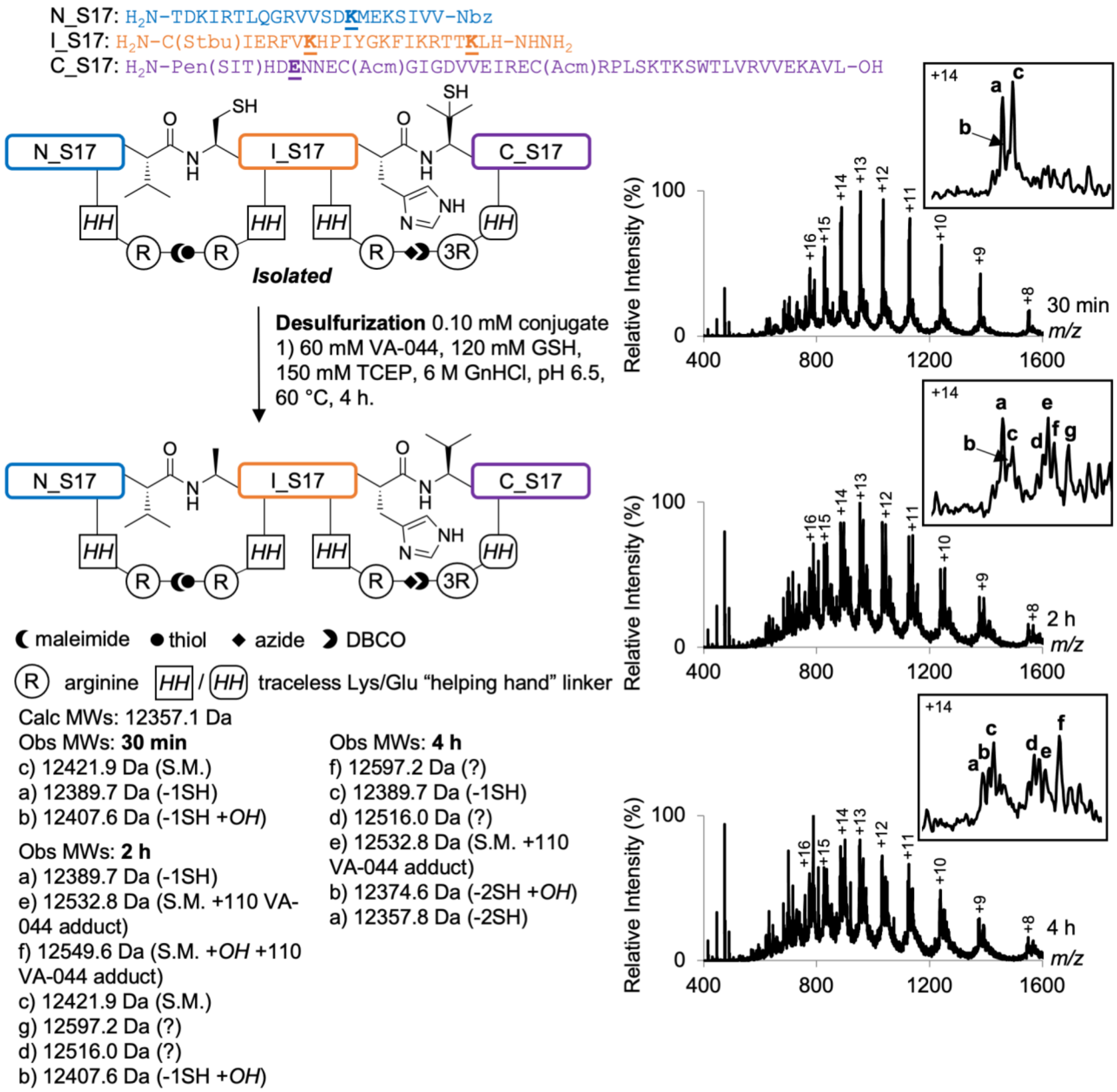

Figure S55. Early desulfurization attempts for the CAPTN synthesis of S17. Desulfurization of purified S17 conjugate was attempted with 60 mM VA-044, 120 mM GSH, and 150 mM TCEP. LC-MS analysis revealed poor conversion to the desired product due to the formation of many unidentified side products (?) and VA-044 adducts (+110 Da). LC-MS method F was used for the analyses. Underlined and bolded Lys (**K**) and Glu (**E**) indicate placements of HH linkers. Nle was substituted for Met in N_S17-Nbz. Italicized *-OH* indicates thioester hydrolysis.

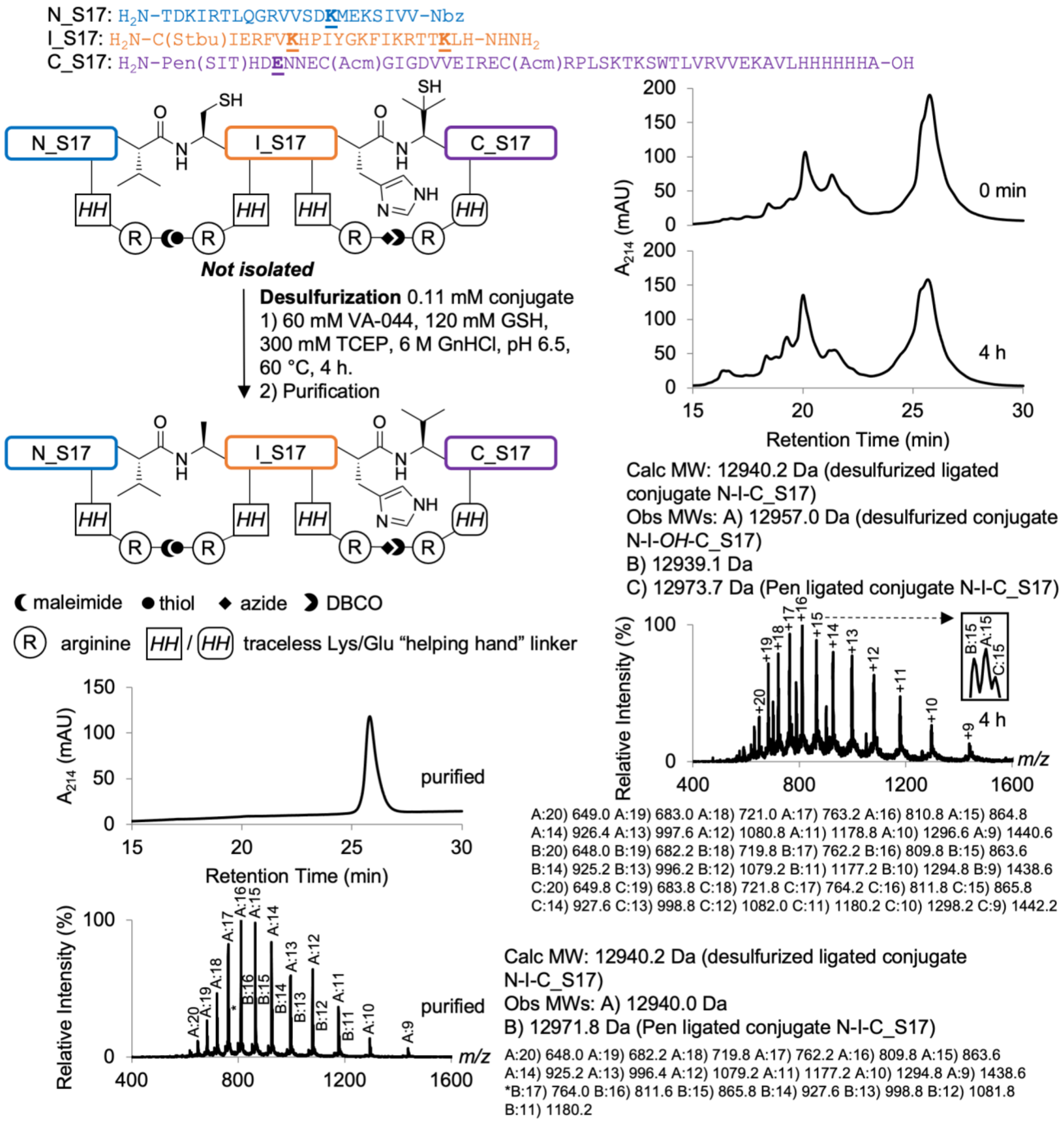

Figure S56. Optimized desulfurization for the CAPTN synthesis of S17. Desulfurization of ligated conjugate N-I-C_S17 post MPAA extraction (Fig S54). The reaction was initiated with 60 mM VA-044 and 120 mM GSH. 300 mM TCEP was added to the reaction which helped minimized VA-044 adducts seen previously (Fig S55). Although incomplete desulfurization was observed, desulfurized ligated conjugate (**25** in main text) was purified by semi-preparative RP-HPLC using purification method V with a 15% isolated yield over five reactions done in one pot. The analytical RP-HPLC chromatogram of the purified desulfurized ligated conjugate N-I-C_S17 is also shown in Fig 5B. Analytical RP-HPLC method K and LC-MS method F were used for the analyses. MS of the product peak from the analytical RP-HPLC chromatogram is reported for the reaction time point. The MS from the entire LC-MS chromatogram is reported for the purified desulfurized ligated conjugate N-I-C_S17. Underlined and bolded Lys (**K**) and Glu (**E**) indicate placements of HH linkers. Nle was substituted for Met in N_S17-Nbz. Italicized -*OH* indicates thioester hydrolysis.

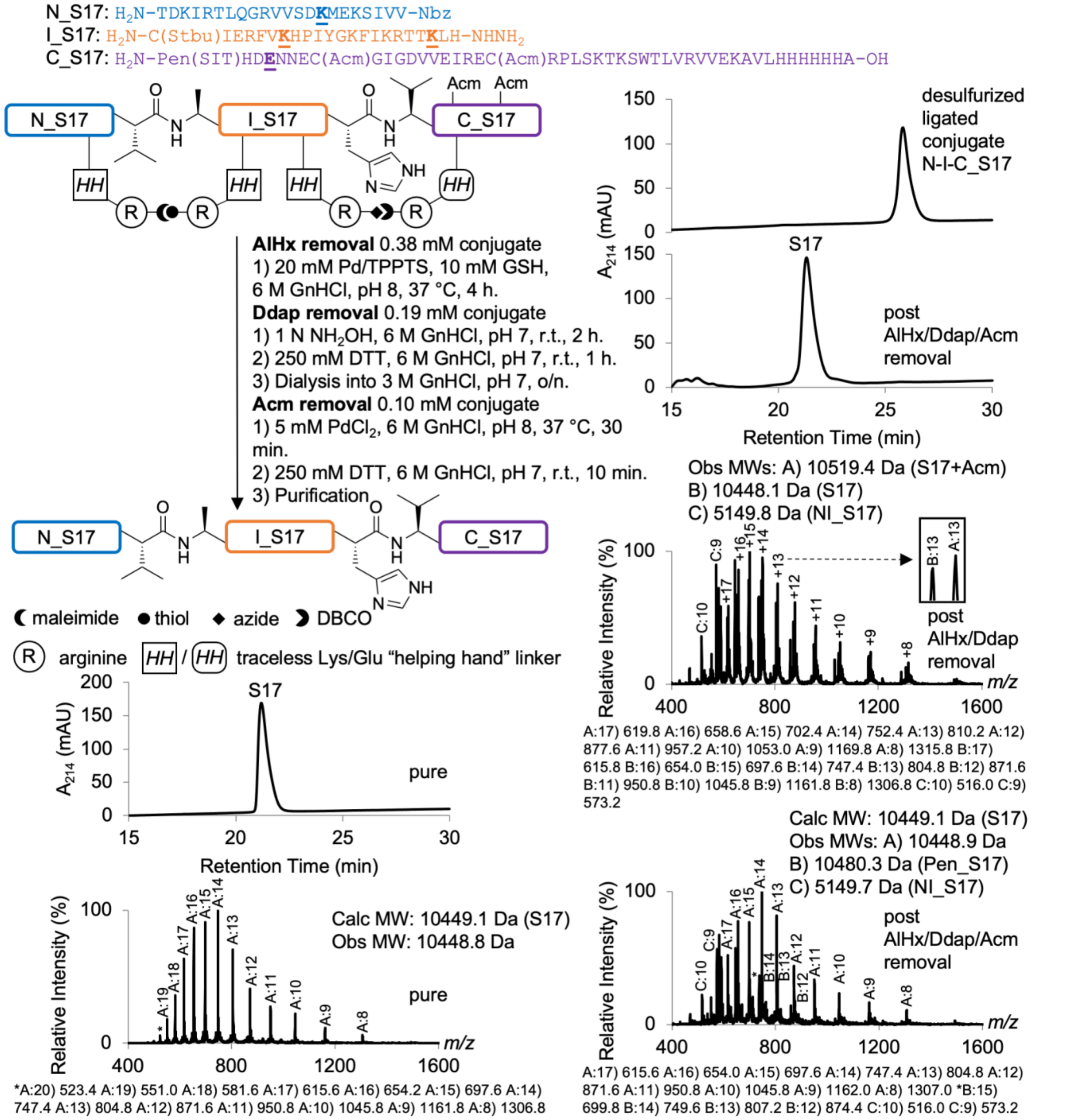

Figure S57. AlHx, Ddap, and Acm removals for the CAPTN synthesis of S17. AlHx removal of desulfurized ligated conjugate N-I-C_S17 (**25** in main text, Fig S56) was performed with 20 mM Pd/TPPTS and 10 mM GSH in 4 h. Ddap removal was then done in 2 h by adding 1 N NH_2OH. The Pd was then quenched with 250 mM DTT after which incomplete Acm removal was observed. To push the reaction to completion, the reaction was dialyzed and then treated with 5 mM $PdCl_2$ for 30 min. The Pd was quenched with 250 mM DTT and the reaction was purified by semi-preparative RP-HPLC using purification method W to isolate S17 (**26** in main text) with 27% isolated yield over three reactions done in one pot. The analytical RP-HPLC chromatogram and MS of pure S17 are also shown in Fig 5B. Analytical RP-HPLC method K and LC-MS method F were used for the analyses. MS of the product peak from the analytical RP-HPLC chromatogram is reported for the reaction time point. The MS from the entire LC-MS chromatogram is reported for pure S17. Underlined and bolded Lys (**K**) and Glu (**E**) indicate placements of HH linkers. Nle was substituted for Met in N_S17-Nbz.

SECTION 5: References

[1] M. T. Jacobsen, P. Spaltenstein, R. J. Giesler, D. H. Chou, M. S. Kay, *Methods Mol. Biol.* **2022**, *2530*, 81-107.
[2] Y. C. Huang, C. C. Chen, S. J. Li, S. Gao, J. Shi, Y. M. Li, *Tetrahedron* **2014**, *70*, 2951-2955.

[3] P. S. Chelushkin, K. V. Polyanichko, M. V. Leko, M. Y. Dorosh, T. Bruckdorfer, S. V. Burov, *Tetrahedron Lett.* **2015**, *56*, 619-622.
[4] J. B. Blanco-Canosa, B. Nardone, F. Albericio, P. E. Dawson, *J. Am. Chem. Soc.* **2015**, *137*, 7197-7209.
[5] P. W. Erickson, J. M. Fulcher, P. Spaltenstein, M. S. Kay, *Bioconjugate Chem.* **2021**, *32*, 2233-2244.
[6] J. M. Fulcher, M. E. Petersen, R. J. Giesler, Z. S. Cruz, D. M. Eckert, J. N. Francis, E. M. Kawamoto, M. T. Jacobsen, M. S. Kay, *Org. Biomol. Chem.* **2019**, *17*, 10237-10244.
[7] R. J. Giesler, P. Spaltenstein, M. T. Jacobsen, W. Xu, M. Maqueda, M. S. Kay, *Org. Biomol. Chem.* **2021**, *19*, 8821-8829.
[8] E. Kaiser, R. L. Colescott, C. D. Bossinger, P. I. Cook, *Anal. Biochem.* **1970**, *34*, 595-598.
[9] J. B. Blanco-Canosa, P. E. Dawson, *Angew. Chem. Int. Ed.* **2008**, *47*, 6851-6855.
[10] A. Chakraborty, A. Sharma, F. Albericio, B. G. de la Torre, *Org. Lett.* **2020**, *22*, 9644-9647.
[11] S. K. Maity, M. Jbara, S. Laps, A. Brik, *Angew. Chem. Int. Ed.* **2016**, *55*, 8108-8112.
